# Prenylation of aromatic amino acids and plant phenolics by an aromatic prenyltransferase from *Rasamsonia emersonii*

**DOI:** 10.1007/s00253-024-13254-8

**Published:** 2024-07-18

**Authors:** Pimvisuth Chunkrua, Kai P. Leschonski, Alejandro A. Gran‐Scheuch, Gijs J. C. Vreeke, Jean-Paul Vincken, Marco W. Fraaije, Willem J. H. van Berkel, Wouter J. C. de Bruijn, Mirjam A. Kabel

**Affiliations:** 1 Laboratory of Food Chemistry, Wageningen University, Bornse Weilanden 9, 6708 WG Wageningen, The Netherlands; 2 Molecular Enzymology Group, University of Groningen, Nijenborgh 4, 9747 AG Groningen, The Netherlands

**Keywords:** DMATS, Fungal PT, aPT, Biocatalysis, Alkylation, Tryptophan

## Abstract

**Abstract:**

Dimethylallyl tryptophan synthases (DMATSs) are aromatic prenyltransferases that catalyze the transfer of a prenyl moiety from a donor to an aromatic acceptor during the biosynthesis of microbial secondary metabolites. Due to their broad substrate scope, DMATSs are anticipated as biotechnological tools for producing bioactive prenylated aromatic compounds. Our study explored the substrate scope and product profile of a recombinant RePT, a novel DMATS from the thermophilic fungus *Rasamsonia emersonii.* Among a variety of aromatic substrates, RePT showed the highest substrate conversion for l-tryptophan and l-tyrosine (> 90%), yielding two mono-prenylated products in both cases. Nine phenolics from diverse phenolic subclasses were notably converted (> 10%), of which the stilbenes oxyresveratrol, piceatannol, pinostilbene, and resveratrol were the best acceptors (37–55% conversion). The position of prenylation was determined using NMR spectroscopy or annotated using MS^2^ fragmentation patterns, demonstrating that RePT mainly catalyzed mono-*O*-prenylation on the hydroxylated aromatic substrates. On l-tryptophan, a non-hydroxylated substrate, it preferentially catalyzed *C*7 prenylation with reverse *N*1 prenylation as a secondary reaction. Moreover, RePT also possessed substrate-dependent organic solvent tolerance in the presence of 20% (*v/v*) methanol or DMSO, where a significant conversion (> 90%) was maintained. Our study demonstrates the potential of RePT as a biocatalyst for the production of bioactive prenylated aromatic amino acids, stilbenes, and various phenolic compounds.

**Key points:**

• *RePT catalyzes prenylation of diverse aromatic substrates.*

• *RePT enables O-prenylation of phenolics, especially stilbenes.*

• *The novel RePT remains active in 20% methanol or DMSO.*

**Graphical abstract:**

**Figure:**
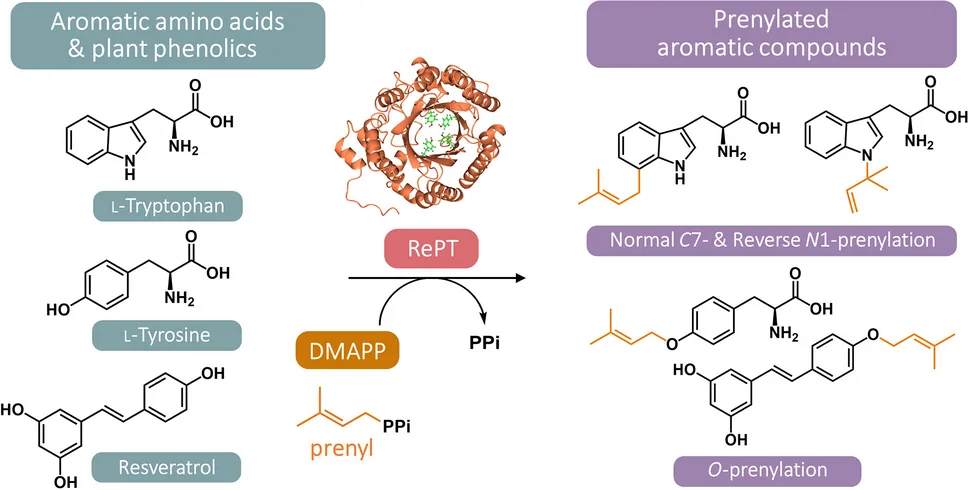

**Supplementary Information:**

The online version contains supplementary material available at 10.1007/s00253-024-13254-8.

## Introduction

Prenylation contributes to the structural diversification of natural products in fungi, bacteria, and plants. In particular, the prenylation of aromatic compounds is a key molecular modification in the biosynthesis of many bioactive secondary metabolites (Winkelblech et al. 2015). This modification is catalyzed by aromatic prenyltransferases (aPTs), which transfer an isoprenoid moiety from a prenyl donor to an aromatic acceptor. The common prenyl donors are dimethylallyl pyrophosphate (DMAPP) and geranyl pyrophosphate (GPP), which are C5- and C10-isoprenoids, respectively (Chen et al. 2017; Eaton et al. 2022). The addition of an isoprenoid moiety increases the lipophilicity of the molecule, consequently increasing its interaction with biological membranes, and likely contributing to enhanced biological activity (Jeong et al. 2018). For example, prenylated (iso)flavonoids and stilbenes from plants from the Fabaceae family (legumes) possess enhanced antimicrobial activity compared to their non-prenylated counterparts and play an important role in plant defense mechanisms against biological threats (Veitch 2007; Ahuja et al. 2012; Araya-Cloutier et al. 2018; de Bruijn et al. 2018).

Due to their potential in pharmaceutical applications, aPTs have gained attention as tools to upscale production or modify drug-related aromatic compounds (de Bruijn et al. 2020; Eggbauer et al. 2022). Non-membrane bound aPTs from the dimethylallyl tryptophan synthase (DMATS) family are appealing due to their broad substrate scope and regioselectivity, rendering them suitable for producing a wide range of compounds for drug discovery and other applications (Chen et al. 2017). Besides, DMATSs differ from other aPT families as their catalytic activity is not dependent on divalent cations. Nevertheless, cations can enhance substrate conversion by these enzymes (Ding et al. 2010; Mundt and Li 2013).

The majority of characterized DMATSs accept l-tryptophan or l-tyrosine, or derivatives thereof, as their natural substrates (Ding et al. 2010; Zou et al. 2011; Pockrandt et al. 2012; Chooi et al. 2012). These aPTs can perform either normal or reverse prenylation on *C*-, *O*-, *N*-, or *S*-positions (Rudolf and Poulter 2013; Kelly et al. 2022).

In the last two decades, the substrate scope of DMATSs has primarily been explored with their natural substrates and structurally related compounds. More recently, studies have shown that a few DMATSs are promiscuous and could be a potent tool for prenylation of biologically-relevant phenolic compounds. For example, AtaPT from *Aspergillus terreus* is known for multi-*C*-prenylation or multi-*C*-geranylation of structurally diverse plant phenolics such as (iso)flavonoids and chalcones (Chen et al. 2017; Li et al. 2022); 7-DMATS_Afu_ from *Aspergillus fumigatus* mainly mono-*C*-prenylates (iso)flavonoids (Yu and Li 2011); AcaPT from *Antrodia camphorata* mainly *O*-prenylates similar substrates as reported for AtaPT (He et al. 2020); and CdpC3PT from *Neosartorya fischeri O*-prenylates anthocyanins (Bao et al. 2021).

We aimed to expand this limited set of promiscuous aPTs. Therefore, in this study, we identified and characterized a new fungal DMATS candidate from the thermophilic fungus *Rasamsonia emersonii*, hereby dubbed RePT*.* We explored its substrate specificity towards aromatic amino acids and structurally diverse plant phenolics. Product profiles were investigated using UHPLC-PDA-MS^2^, and NMR was used to confirm the structure of several major products. The tolerance of the enzyme to organic solvents was also explored as a relevant property for its potential future application in the upscaled production of prenylated compounds.

## Materials and methods

### Materials

Compounds tested for the substrate scope of RePT were purchased from various suppliers (Table S1). DMAPP-ammonium salt and GPP-lithium salt used in the biochemical characterization of RePT were purchased from Sigma–Aldrich (St. Louis, MO, USA). DMAPP-triammonium salt used in the upscaled production of prenylated products was synthesized using a method as described in Supplementary Information. Other chemicals were purchased from Sigma–Aldrich and Fisher Scientific (Landsmeer, The Netherlands). UHPLC-MS grade solvents used in UHPLC-PDA-MS analyses were purchased from Biosolve (Valkenswaard, The Netherlands). Water for all purposes other than UHPLC-PDA-MS was prepared using a Merck Millipore Milli-Q water purification system (Billerica, MA, USA).

### Genome mining

To identify putative DMATSs, an NCBI protein BLAST search was performed using AtaPT (GenBank accession number: AMB20850.1) as a template. Among the sequences with the highest sequence identity, the ones from thermotolerant fungi were pre-selected. The putative 7-DMATS from *Rasamsonia emersonii* CBS 393.64 (NCBI accession number: XP_013322739.1), with a 30.2% sequence identity with AtaPT, was finally selected for further characterization, since no DMATS from this genus had been characterized previously, while this ascomycete has been reported to produce a wide range of heat-resistant enzymes (Murray et al. 2004; Houbraken et al. 2012). Multiple sequence alignment of RePT and 19 DMATSs was performed using Mega7 software, to confirm that the sequence of RePT contained all expected conserved amino acids.

### Gene cloning, enzyme expression, and enzyme purification

The putative 7-DMATS-encoding DNA sequence was synthesized by Twist Bioscience after being codon-optimized for *E. coli* using their bioinformatic tool (South San Francisco, CA, USA) (Table S2). The obtained gene was cloned into a pBAD vector encoding an N-terminal His-tag linked with SUMO fusion protein (pBAD-His-SUMO) using Golden Gate Assembly. The recombinant plasmid was then transformed into chemically competent *E. coli* NEB 10-beta cells (New England Biolabs, Ipswich, MA) using heat shock. After transformation, the plasmid was purified by using a QIAprep Spin Miniprep Kit (QIAGEN, Venlo, The Netherlands). The plasmid was sent to Eurofins Scientific for sequencing, and the sequence was verified by using Geneious Prime (version 2019.2). The protein, RePT, was produced in an 8 mL preculture containing 8 mL of lysogeny broth (10 g/L tryptone soya broth, 5 g/L yeast extract, 10 g/L NaCl) and 0.05 mg/mL ampicillin. The preculture was incubated (37 °C, 16 h, 200 rpm) and later transferred to an 800 mL culture containing 800 mL of Terrific broth (12 g/L tryptone soya broth, 24 g/L yeast extract, 4.78 g/L glycerol, 2.3 g/L KH_2_PO_4_, 16.4 g/L K_2_HPO_4_ 3H_2_O) and ampicillin to a final concentration of 0.05 mg/mL. The culture was incubated at 37 °C with shaking until the OD_600_ reached 0.6–0.8. Gene expression was then induced by adding l-arabinose to a final concentration of 0.2 mg/mL. Subsequently, the induced culture was incubated for 40 h (24 °C, 200 rpm). Cells were harvested by centrifugation (6000 × *g*, 4 °C, 20 min). The pelleted cells were resuspended in 40 mL lysis buffer (50 mM Tris/HCl pH 7.5, 500 mM NaCl, 10% (*w/v*) glycerol, 1 g/L lysozyme, 1 mM DTT). Cells were lysed by sonication (55% amplitude, 5 s pulse on, 10 s pulse off for 10 min) and centrifuged (16,000 × *g*, 4 °C, 1 h). The supernatant was filtered with a 0.45 µm filter, and the cell-free extract was collected for further purification.

Imidazole was added to the cell-free extract to a final concentration of 25 mM. The extract was then loaded onto a 5 mL HisTrap FF column (Cytiva, Uppsala, Sweden) connected to an ÄKTA Pure M150 system (Marlborough, MA, USA). The flow rates were 10 mL/min and 5 mL/min for sample loading and elution, respectively. Eluents used were as follows: (A) 50 mM Tris/HCl pH 7.5, 500 mM NaCl, 10% (*w/v*) glycerol, 25 mM imidazole; and (B) 50 mM Tris/HCl pH 7.5, 500 mM NaCl, 10% (*w/v*) glycerol, 500 mM imidazole. RePT elution was performed with a 5-column volume of a linear gradient from 0 to 100%B. After purification, the pooled RePT fractions were desalted by using Amicon ultra-centrifugal filters with 30 kDa molecular weight cut-off (Merck Millipore, Billerica, MA, USA) and desalting buffer (50 mM Tris/HCl pH 7.5, 10% (*w/v*) glycerol) with several rounds of centrifugation (4000 × *g*, 4 °C, 15 min each). The apparent subunit molecular mass and purity of RePT were then analyzed by using sodium dodecyl sulfate–polyacrylamide gel electrophoresis (SDS-PAGE) with polyacrylamide gels (Any kD Mini-PROTEAN TGX Precast Protein Gels, Bio-Rad Laboratories, Hercules, CA, USA) and a protein marker (Precision Plus Protein, Bio-Rad Laboratories). The protein content was determined with a BCA protein assay kit, and bovine serum albumin was used for calibration (Pierce BCA Protein Assay Kit, Thermo Scientific, Rockford, IL, USA). SDS-PAGE and protein content determination were performed according to the supplier’s manual.

### Enzyme purity and molecular mass determination, and sequence confirmation with UPLC-PDA-MS

To determine the exact molecular mass and purity of RePT, the purified enzyme solution was analyzed with an Acquity Premier ultra-high performance liquid chromatography (UHPLC) equipped with a photodiode array detector (PDA) and the Select Series Cyclic IMS ion mobility mass spectrometer (Waters, Milford, MA, USA). The preparation and conditions used for the UPLC-PDA-MS were similar as described previously (Vreeke et al. 2023). The purity of RePT was determined based on UV 214 nm peak area. The molecular mass determination was performed with mass deconvolution on the MS spectrum using the MaxEnt function in MassLynx software. Additionally, the sequence of RePT was confirmed using the Vreeke method (Vreeke et al. 2022). First, RePT was hydrolyzed by porcine trypsin at 37 °C or by *Bacillus licheniformis* protease (BLP) at 40 °C, both at protease to substrate ratio of 1:25. The obtained hydrolysates were mixed 1:1 (*v:v*) with a 100 mM Tris/HCl buffer pH 8.0 containing 20 mM DTT and then incubated for 2 h. Afterwards, the hydrolysates were diluted and acidified to pH 2, and subsequently centrifuged for 10 min at 14,000 × *g*. 4 µL of the supernatant was injected on the UPLC-PDA-MS. The mass spectra were annotated to peptide sequences using UNIFI software version 1.8 according to the guidelines and criteria for MS/MS fragments as previously described by Vreeke et al. (2022). The UV peak area at 214 nm and the predicted molar extinction coefficient were used to quantify each peptide.

### Substrate specificity of RePT

Prenyltransferase activity was assessed on a wide range of potential substrates in 100 µL of a 50 mM Tris/HCl buffer (pH 7.5) mixture containing a final concentration of 0.4 mM aromatic acceptor, 0.8 mM prenyl donor, and 100 µg RePT. To standardize the semi-quantification of substrates and products, 3,4,5-trimethoxycinnamic acid (TMCA) was used as an internal standard in all enzymatic reactions and was added to a final concentration of 0.16 mM. TMCA was confirmed to not be a substrate of RePT or detrimental to RePT’s activity and was stable over 48 h in the reaction condition used (Figs. S1–S3). Some reaction mixtures contained up to 4% (*v/v*) methanol or DMSO, or 0.4 mM NaOH depending on the solvent in which the substrate was dissolved. In the experiment on the effects of divalent cations, either 5 mM CaCl_2_ or 5 mM MgCl_2_ was added to the reaction. The reaction mixture was stirred at 37 °C in an incubation shaker for 24 h at 700 rpm. The reaction was then terminated by adding 200 µL of methanol and centrifuging with a table-top centrifuge (4 °C, 18,000 × *g*, 15 min). The supernatant was collected for further analysis with RP-UHPLC-PDA-ESI-IT-MS^n^ (see below).

### Substrate conversion and product profile analysis by RP-UHPLC-PDA-ESI-IT-MS^n^

Substrates and their prenylated derivatives were analyzed with a Thermo Vanquish UHPLC system (Thermo Scientific, San Jose, CA, USA) coupled to a PDA detector. The supernatants obtained after enzyme incubation (1 μL) were loaded onto an Acquity UPLC BEH C18 column (150 mm × 2.1 mm, 1.7 μm) coupled with a VanGuard pre-column of the same material (5 mm × 2.1 mm, 1.7 μm) (Waters, Milford, MA, USA). The column temperature was set at 45 °C with post-column cooling to 40 °C. The PDA detector was set to record a wavelength range of 200–600 nm. The flow rate used was 400 µL/min with the following eluents: (A) water containing 0.1% (*v/v*) formic acid and (B) acetonitrile containing 0.1% (*v/v*) formic acid. The elution gradient was as follows: 1 min isocratic at 1%B; 28 min linear gradient from 1 to 80%B; 1 min linear gradient from 80 to 100%B; 5.5 min isocratic 100%B; 1 min linear gradient from 100 to 1%B; and 5.5 min isocratic at 1%B.

Mass spectrometric analysis was performed by in-line coupling of the Thermo Vanquish UHPLC system to an LTQ Velos Pro ion trap mass spectrometer (IT-MS) (Thermo Scientific, San Jose, CA, USA) equipped with a heated electron spray ionization (ESI) probe. Nitrogen was used as sheath gas (50 arbitrary units), auxiliary gas (13 arbitrary units), and sweep gas (1 arbitrary unit). Ion transfer tube temperature of 263 °C, source heater temperature of 425 °C, and source voltages of 2.5 kV (negative ionization) and 3.5 kV (positive ionization) were used. Full scan MS data was acquired in positive ionization mode (PI) and negative ionization mode (NI) in the range of *m/z* 100–1000 or 200–1000 depending on the molecular mass of the substrates. To obtain MS^2^ spectra of (non-)prenylated compounds, data-dependent collision-induced dissociation (CID) fragmentation was performed on the most abundant MS^1^ ion with a normalized collision energy of 35%. Dynamic mass exclusion was used to also obtain MS^2^ spectra of less intense ions present in MS^1^. The most intense ion was fragmented 3 times with a repeat duration of 5 s and then excluded from fragmentation for 5 s. Data processing was performed with Xcalibur (version 4.4, Thermo Scientific).

Substrate recovery (%), substrate conversion (%), and relative product formation (%) were calculated using the peak area from UV 280 nm, otherwise specified, with the following equations:

1 $$\text{Substrate recovery}\;\left(\%\right)=\frac{{\mathrm{Area}}_\mathrm{fbl}-{\mathrm{Area}}_\mathrm{bl}}{{\mathrm{Area}}_\mathrm{fbl}}\times100$$

2 $$\text{Substrate conversion}\;\left(\%\right)=\frac{{\mathrm{Area}}_\mathrm{bl}-{\mathrm{Area}}_\mathrm{r}}{{\mathrm{Area}}_\mathrm{bl}}\times100$$

3 $$\text{Relative product formed}\;\left(\%\right)=\text{substrate conversion }\times\frac{{\mathrm{Area}}_\mathrm{pr}}{{\mathrm{Area}}_\mathrm{totpr}}\times100$$

The abbreviations are as follows: Area_fbl_ = peak area of the substrate in a blank (without RePT and prenyl donor) without an incubation; Area_bl_ = peak area of the substrate in a blank incubated at the same reaction condition as the reaction mixture; Area_r_ = peak area of the substrate in the reaction mixture; Area_pr_ = peak area of a prenylated product; and Area_totpr_ = total peak area of prenylated products.

### Upscaled production of prenylated products

Prior to the larger production of prenylated products for NMR analysis, different concentrations of l-tryptophan (**1**) were tested to find an optimal substrate concentration for the upscaled reaction. Detailed information of the upscaling production of prenylated products can be found in Supplementary Information.

### Structure elucidation of prenylated products with NMR spectroscopy

An amount of 1.5 mg of purified prenylated product was dissolved in 570 µL of 70% (*v/v*) methanol-d_4_ in D_2_O (Sigma–Aldrich, St. Louis, MO, USA) or 100% DMSO-d_6_ (Eurisotop, Saint–Aubin, France). NMR spectra were recorded using a Bruker Avance-III-600 spectrometer with a cryoprobe (Bruker, Billerica, MA, USA) located at the Magnetic Resonance Research Facility of Wageningen University (MAGNEFY, Wageningen, The Netherlands). The probe temperature used was 300 K. Data acquisition was performed for 1D ^1^H and ^13^C, and 2D HMBC and HSQC spectra. Data processing was performed using TopSpin (version 4.1.4, Bruker).

### Accurate mass analysis by RP-UHPLC-PDA-ESI-FT-MS

The purified prenylated products were solubilized in a range of 16–20 mM in suitable solvents (DMSO or 20 mM NaOH in water in case of prenylated l-tyrosine) and further diluted to a final concentration of 0.01 mM in methanol. To determine the accurate mass, the solution (1 µL) was then loaded onto the Vanquish RP-UHPLC system (Thermo Scientific, San Jose, CA, USA) coupled to a PDA detector and a high-resolution Orbitrap mass spectrometer (FT-MS). The column used was identical to the column used for IT-MS^n^ analysis. The column temperature was set at 35 °C. The flow rate used was 400 µL/min with the following eluents: (A) water containing 1% (*v/v*) formic acid and (B) acetonitrile containing 1% (*v/v*) formic acid. The elution program was as follows: 2.1 min isocratic at 5%B; 18.2 min linear gradient from 5 to 55%B; 1.1 min linear gradient from 55 to 100%B; 5.5 min isocratic 100%B; 1.1 min linear gradient from 100 to 5%B; and 5.5 min isocratic at 5%B.

The accurate mass of the selected prenylated products was determined using in-line coupling of the Thermo Vanquish UHPLC system to a Thermo Q Exactive Focus Hybrid Quadrupole-Orbitrap mass spectrometer (Thermo Scientific, San Jose, CA, USA) equipped with a heated ESI probe. Nitrogen was used as sheath gas (48 arbitrary units) and auxiliary gas (11 arbitrary units). An ion transfer tube temperature of 256 °C, probe heater temperature of 413 °C, spray voltage of 3.5 kV, and S-Lens RF level of 50% were used. Full scan MS data was acquired in the range of *m/z* 200–1000 in PI mode. Mass calibration was performed using Pierce LTQ ESI positive ion calibration solution (Thermo Scientific). Full MS was recorded at 70,000 resolution. Data processing was performed with Xcalibur (version 4.4, Thermo Scientific).

### Structural modeling of RePT

The three-dimensional structure of RePT was modeled using AlphaFold2 (Jumper et al. 2021) using the CASP14 settings. The model was generated and provided by Dr. Hein Wijma, University of Groningen.

## Results

### Identification, sequence analysis, and structural modeling of RePT from *Rasamsonia emersonii*

A multiple sequence alignment was performed to compare the amino acid sequence of RePT with 19 characterized aPTs from the DMATS family (Fig. S4). Two strictly conserved key regions were defined, which were (i) the four residues involved in prenyl-donor binding and (ii) the four tyrosine residues proposed to be required for stabilizing the dimethylallyl carbocation intermediate (Jost et al. 2010; Chen et al. 2017; Eaton et al. 2022). RePT indeed contained all four positively charged residues for binding the pyrophosphate moiety of the prenyl donor (i.e., R133, K214, R284, K286), as well as the four tyrosine residues (i.e., Y216, Y288, Y374, Y445). The strictly conserved glutamate (E120) (Metzger et al. 2009; Jost et al. 2010; Eaton et al. 2022) was also detected in the amino acid sequence of RePT. This residue is suggested to be involved in binding the indole nitrogen of l-tryptophan (a prenyl acceptor) via hydrogen bonding at the enzyme’s functional pH (Metzger et al. 2009; Eaton et al. 2022). From Fig. S4, it was also concluded that RePT showed the highest sequence identity with 7-DMATS_Afu_ (44%).

The structural model of RePT generated using AlphaFold2 clearly indicated an ABBA-fold, which is characterized by the repeating α-β-β-α units with antiparallel strands, a common architectural feature of DMATSs (Fig. S5). Therefore, based on both the sequence alignment and predicted structure, the putative 7-DMATS was expected to catalyze prenylation of l-tryptophan and possibly other aromatic compounds. The rather long N-terminal extension (about 30 residues) of RePT was predicted with a low pLDDT confidence score, indicating a possible disordered region.

Comparison with the promiscuous AtaPT revealed that the four conserved tyrosine residues (i.e., Y216, Y288, Y374, Y445) located in the catalytic chamber of RePT were oriented in a similar position (Fig. S6). The electrostatic surface potential of both RePT and AtaPT showed a highly positively charged region composing the four key residues (i.e., R133, K214, R284, and K286) and other residues required to bind the pyrophosphate moiety of the prenyl donor. The aromatic acceptor binding pocket of RePT has relatively more charge than that of AtaPT, which contains a larger hydrophobic region. The active site architecture and possible key residues for the prenylation reaction of RePT are presented in Fig. S7, and the comparison with other DMATSs is presented in Fig. S8.

### RePT production, protein purity, and confirmation of the amino acid sequence

RePT was successfully expressed and produced in *E. coli* NEB10-beta and purified via nickel affinity chromatography, yielding 12 mg protein per liter of culture. Based on SDS-PAGE, the apparent subunit molecular mass of His-SUMO-RePT was 66 kDa (Fig. S9). The precise molecular mass was 66.40 kDa, according to mass spectrometric analysis of the intact enzyme. The experimentally determined mass was similar to the theoretical molecular mass of 66.41 kDa (Fig. S10). The purity of RePT was determined based on the UV 214 nm peak area in UPLC-PDA-MS analysis to be 99.1%. To confirm the amino acid sequence, His-SUMO-RePT was digested and the peptides were subsequently analyzed with UPLC-PDA-MS, according to the procedure adapted from Vreeke et al. (2022). Protein digestion with porcine trypsin and BLP yielded 131 and 174 unique peptides, respectively (Fig. S11). The amino acid sequence coverages of His-SUMO-RePT were 98.1% (porcine trypsin) and 99.3% (BLP) (Fig. S12). The non-covered sequences did not overlap between the two hydrolysates. Therefore, by combining the data, we achieved 100% coverage of the sequence starting from the His-tag. The confirmed amino acid sequence is shown in Table S2.

### Biochemical characterization of RePT

To find the optimum pH for catalysis, RePT was tested at pH 6.5, pH 7.5, and pH 7.9. These pH values were selected based on the pH values at which AcaPT, another DMAT, yielded at least 80% relative activity (He et al. 2020). Two substrates were used: l-tryptophan (**1**) and resveratrol (**8**). For both substrates, conversion was optimal at pH 7.5 (Figs. S13–S14). Thus, the substrate specificity was further assessed at pH 7.5. The conversion of **1** remained the same (100%) with or without adding 5 mM EDTA to the reaction mixture, indicating that RePT did not require cations for its reaction (data not shown). However, as also demonstrated for other DMATSs (Yin et al. 2013; Mundt and Li 2013), conversion by RePT was increased by adding divalent cations, where the conversion of **8** was 2.5 or 2 times higher in the presence of 5 mM CaCl_2_ or 5 mM MgCl_2_, respectively (Fig. S15b–c). For upscaling, the incubation was tested with different concentrations of **1** (0.1–20 mM), a constant ratio of DMAPP to **1** (2:1), and an RePT concentration of 0.1 mg/mL (Fig. S16). At acceptor substrate concentrations of 1 mM and below, conversion was 98–100%. However, at higher substrate concentrations of **1**, the relative conversion by RePT decreased, likely due to substrate inhibition.

### Substrate-dependent organic solvent tolerance

**Fig. 1:**
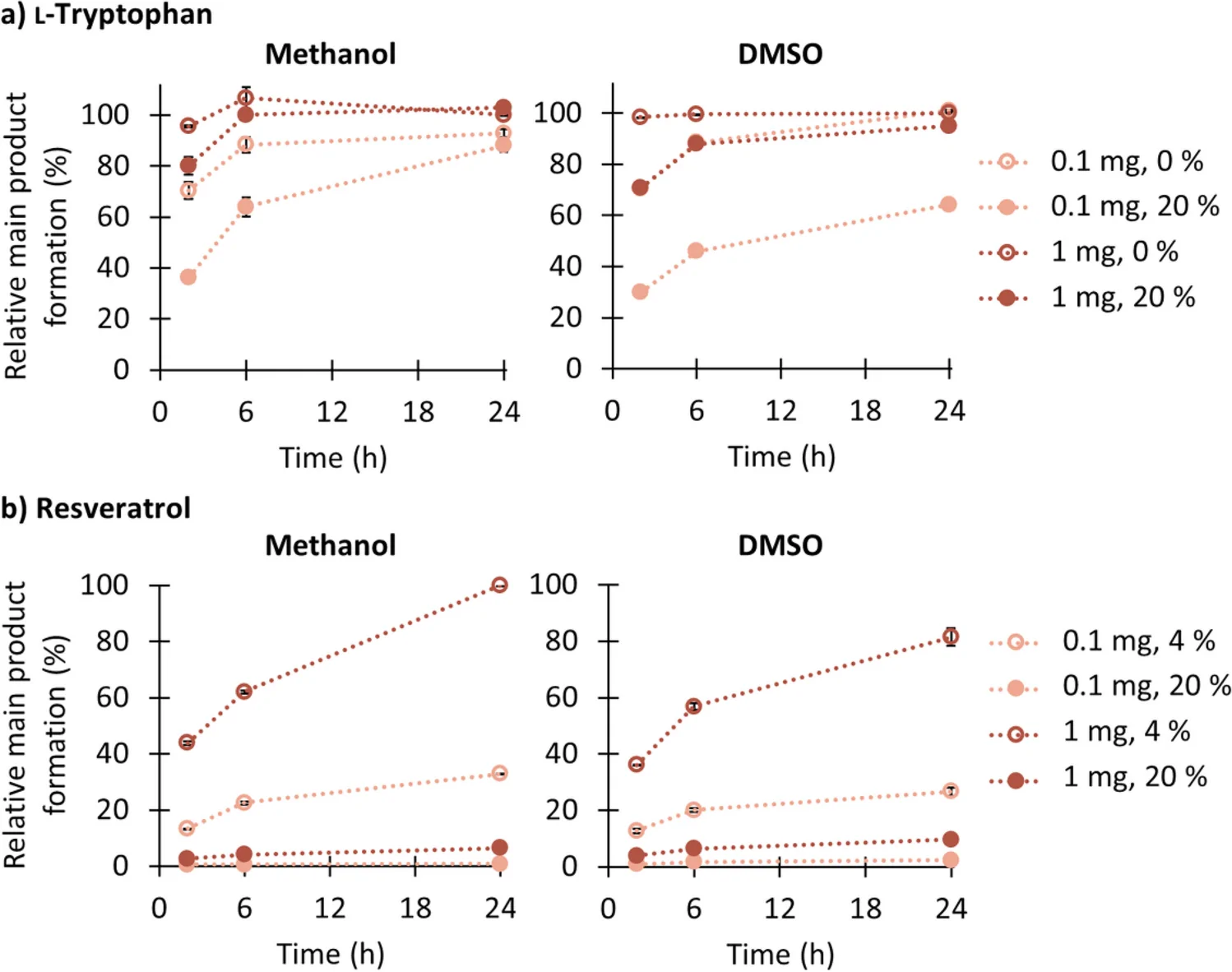
Time-dependent formation of the main prenylated product from an incubation with two RePT concentrations (0.1 and 1.0 mg/mL) in the presence of DMAPP with l-tryptophan (**1**) with 0% or 20% methanol or DMSO (**a**) and resveratrol (**8**) with 4% or 20% methanol or DMSO (**b**)

As most of the aromatic acceptors are relatively poorly water-soluble, we incubated **1** (water-soluble, best substrate) and **8** (poorly water-soluble substrate) with an additional 20% (*v/v*) methanol or DMSO to investigate whether these cosolvents are beneficial for the conversion (Fig. 1). RePT exhibited slightly higher methanol tolerance compared to DMSO for both substrates, although both solvents had a similar impact on product formation by RePT. For substrate **1**, in the presence of 20% (*v/v*) of methanol, RePT still retained > 90% of its relative product formation (Fig. 1a). Although the solvents slowed down the product formation over time, they did not completely inhibit the enzyme activity after 2 and 6 h. This effect was more explicit when a lower enzyme concentration (0.1 mg/mL) was used. However, when **8** was used as a substrate, adding 20% (*v/v*) of either organic solvent limited the relative product formation to less than 10% after 24 h (Fig. 1b). As the addition of organic solvents did not help improve substrate conversion by RePT at the substrate concentration tested, a maximum of % (*v/v*) organic solvent was used in further experiments for poorly water-soluble substrates. Substrate-dependent tolerance up to 25% (*v/v*) DMSO was previously reported for another DMATS, DmaW (Eggbauer et al. 2022).

### Substrate and prenyl donor specificity of RePT

**Fig. 2:**
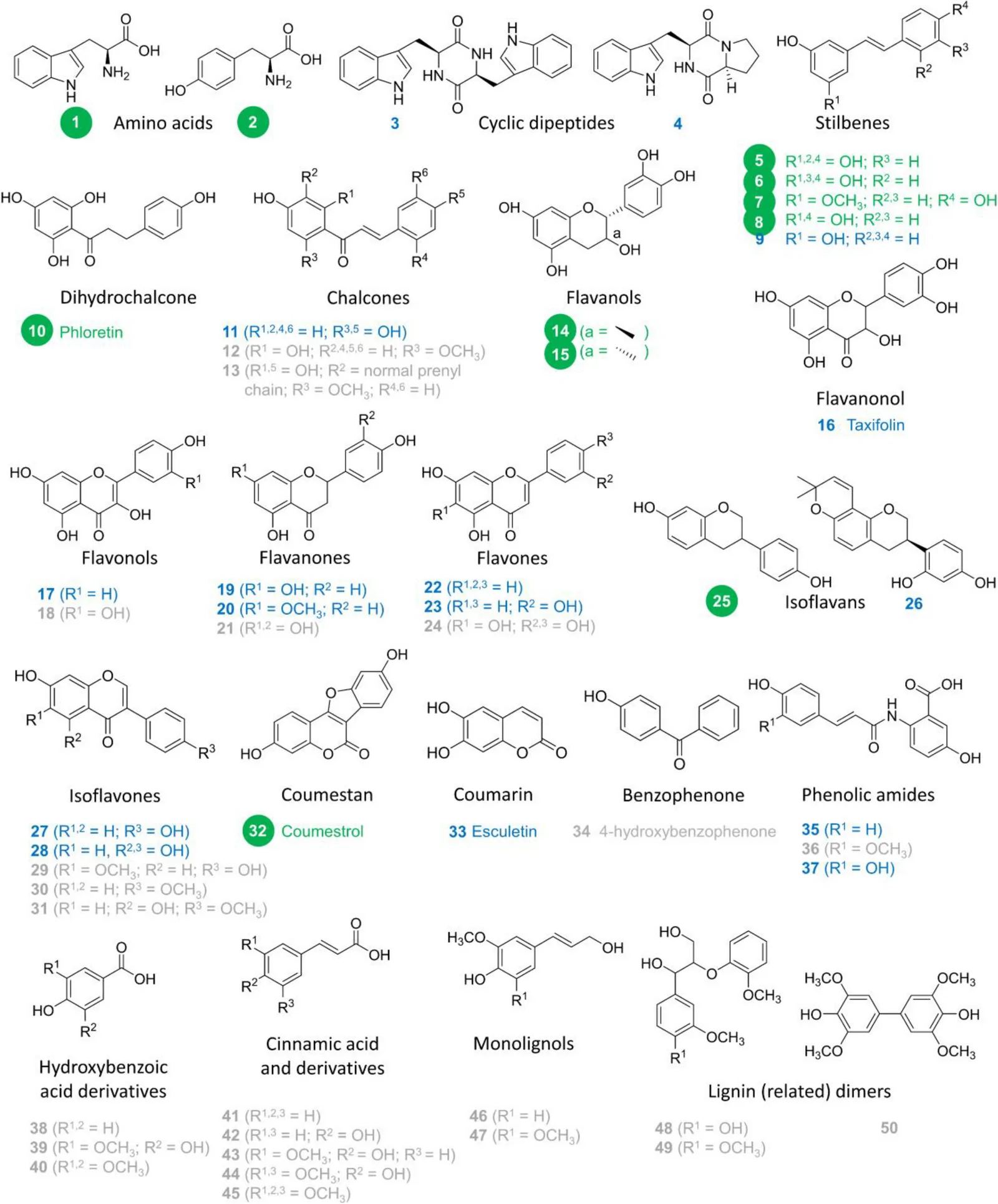
Aromatic compounds tested as acceptor substrates for RePT. Compounds accepted with more than 10% conversion, between 1 and 10%, and less than 1% are shown in green, blue, and gray, respectively. For detailed information of the 50 aromatic compounds tested, see Table S1

**Fig. 3:**
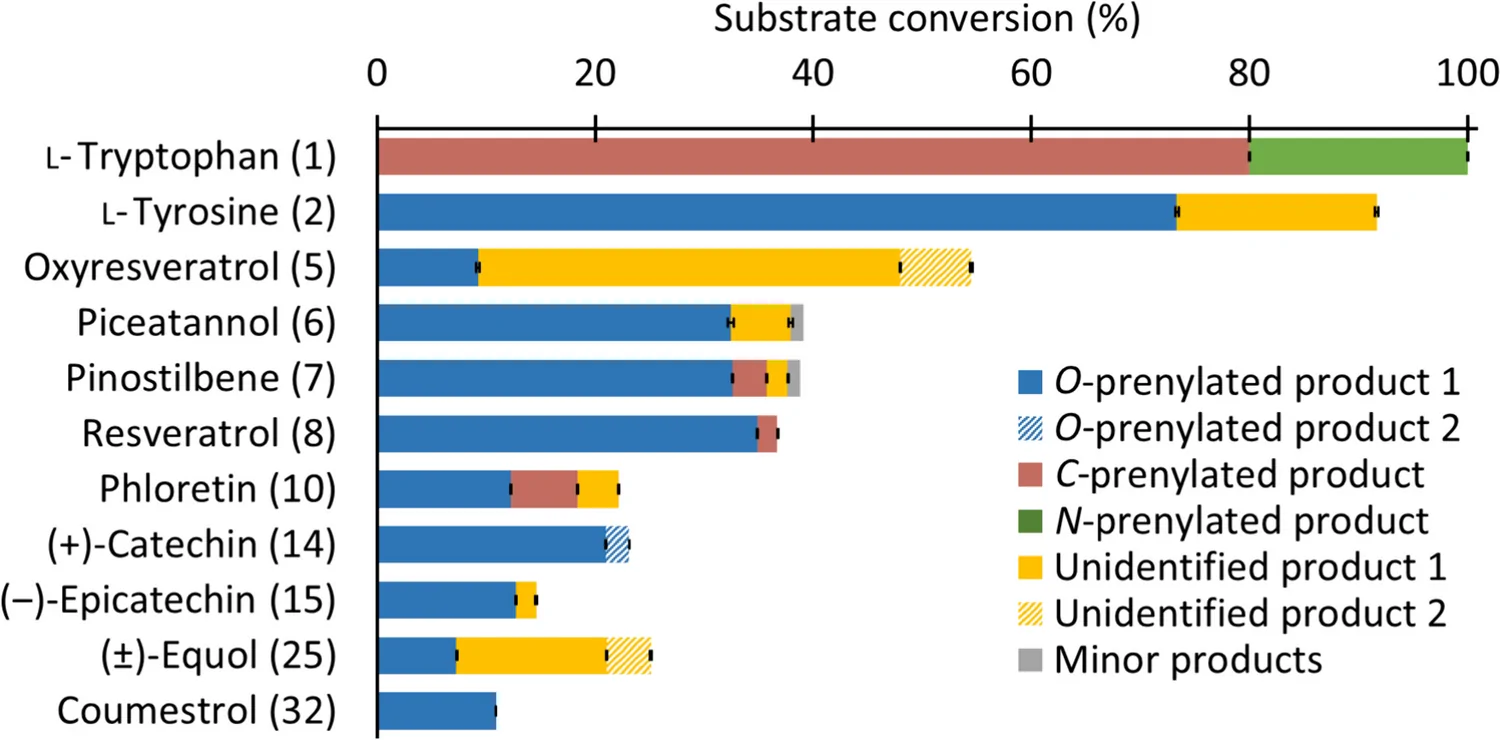
Substrate conversion (%) and relative amount of each product formed in relation to substrate conversion (%). The standard deviations for substrate conversion are shown in Table S3. Prenylation positions were confirmed by NMR spectroscopy (two products of **1**, and the main products of **2** and of **8**) or annotated using UHPLC-PDA-MS^2^. Minor products are prenylated products with a UV peak area less than 5% of the total product UV peak area

In this study, we explored the prenyl acceptor substrate scope of RePT using structurally diverse aromatic compounds. Tested substrates included typical DMATS substrates such as aromatic amino acids and derivatives, and phenolics from diverse subclasses such as (iso)flavonoids, stilbenes, coumarins, hydroxybenzoic acid derivatives, and hydroxycinnamic acid derivatives. The compounds were first tested with DMAPP, and subsequently, a few compounds were selected for further testing with GPP. An overview of the substrate conversion (%) observed with a wide variety of acceptor substrates is shown in Fig. 2. The exact conversion (%) of the accepted substrates, i.e., those with > 10% conversion by RePT in the presence of DMAPP, are shown in Fig. 3. The UHPLC-PDA-MS^n^ chromatograms and data of the enzymatic reactions are presented in Fig. S17 and Table S3. Substrate recovery (%) of the eleven well-accepted substrates can be found in Table S4 and was taken into account in the calculation of substrate conversion.

RePT showed the highest conversion for the two aromatic amino acids l-tryptophan (**1**) (100.0 ± 0.0%) and l-tyrosine (**2**) (91.7 ± 0.3%), whereas the conversion with two cyclic dipeptides (CDPs), cyclo(l-Trp- l-Trp) (**3**) and cyclo(l-Trp-l-Pro) (**4**) was less than 10%. Among the 46 phenolics tested, 10 phenolics from diverse subclasses were accepted with noticeable conversion (> 10%). The highest conversion was found for four stilbenes: oxyresveratrol (**5**), piceatannol (**6**), pinostilbene (**7**), and resveratrol (**8**), with 54.5 ± 0.4%, 39.1 ± 1.6%, 38.8 ± 0.5%, and 36.7 ± 0.5% conversion, respectively. Conversions in the range of 11 to 25% were observed for prenyl acceptors from several other phenolic subclasses namely phloretin (**10**), a dihydrochalcone; ( +)-catechin (**14**) and (‒)-epicatechin (**15**), flavan-3-ols; ( ±)-equol (**25**), an isoflavan; and coumestrol (**32**), a coumestan.

Fourteen phenolics were accepted with low conversion (< 10%). These included a stilbene (**9**), a chalcone (**11**), a flavanonol (**16**), a flavonol (**17**), two flavanones (**19**–**20**), two flavones (**22**–**23**), a ring-prenylated isoflavan (**26**), two isoflavones (**27**–**28**), a coumarin (**33**), and two phenolic amides (**35**, **37**). Hydroxybenzoic derivatives, cinnamic acid derivatives, and monolignols (**38**–**47**), or other molecules with at least two methoxy groups (**48**–**50**) were very poorly converted by RePT (< 1%).

Our findings suggest that RePT exhibits a preference for relatively small and flexible substrates, particularly aromatic amino acids and stilbenes. Additional trends in substrate preference were also identified. The occurrence of two hydroxyl groups adjacent to one another (*ortho*-dihydroxy) seems to have a negative effect on conversion, as illustrated by the lower conversion of the *ortho*-dihydroxy substituted **5** compared to that of the *meta*-dihydroxy substituted **6**. Although methoxylated molecules in general seem to be poorly accepted, the effect of methoxylation on the conversion remains unclear. A comparable conversion of **7** (methoxylated) and **8** (non-methoxylated) might indicate that a methoxy group in the *meta* position to the hydroxyl group does not interfere with binding. Conversely, in the case of dihydroxy molecules, a methoxy group at the *ortho* position to the hydroxyl groups reduces conversion, as seen when comparing **27** and **29**. Several ligand-bound aPT crystal structures show that three or four hydrogen bonding sites are involved in the binding of preferred substrates (Metzger et al. 2009; Chen et al. 2017; Eaton et al. 2022). Thus, the decreased conversion of **42**, which lacks the α-amino group compared to **2**, would likely relate to the reduced hydrogen bonding capacity. It has previously been shown that the α-amino group of the aromatic amino acid substrate forms hydrogen bonds with one or two carbonyl side chains of the residues in the catalytic chamber of DMATSs (Metzger et al. 2009; Eaton et al. 2022). Even a different configuration at one chiral center already affects the conversion as seen by comparison of **14** (23.3 ± 0.3%) and **15** (14.6 ± 0.7%).

To determine prenyl donor specificity, we selected **1** and **8** to further test with GPP. However, conversions in the presence of GPP were low, namely 4.1 ± 0.2% for **1** and 7.0 ± 0.6% for **8** (data not shown). Therefore, we did not further investigate the acceptance of GPP or larger prenyl donors.

### Prenylated product profile and regioselectivity

RePT catalyzed mostly mono-prenylation on aromatics, with an exception of daidzein (**27**), an isoflavone, for which a small amount of diprenylated product was observed (data not shown). For most compounds, RePT generated one main prenylated product, i.e., one product with ≥ 70% relative abundance (RA), and minor amounts of other products. Exceptions were **32** which yielded only one product, and **10** and **25** which yielded no distinct main product, but instead, three products with 15–55% RA.

#### Structure elucidation of selected prenylated products

**Fig. 4:**
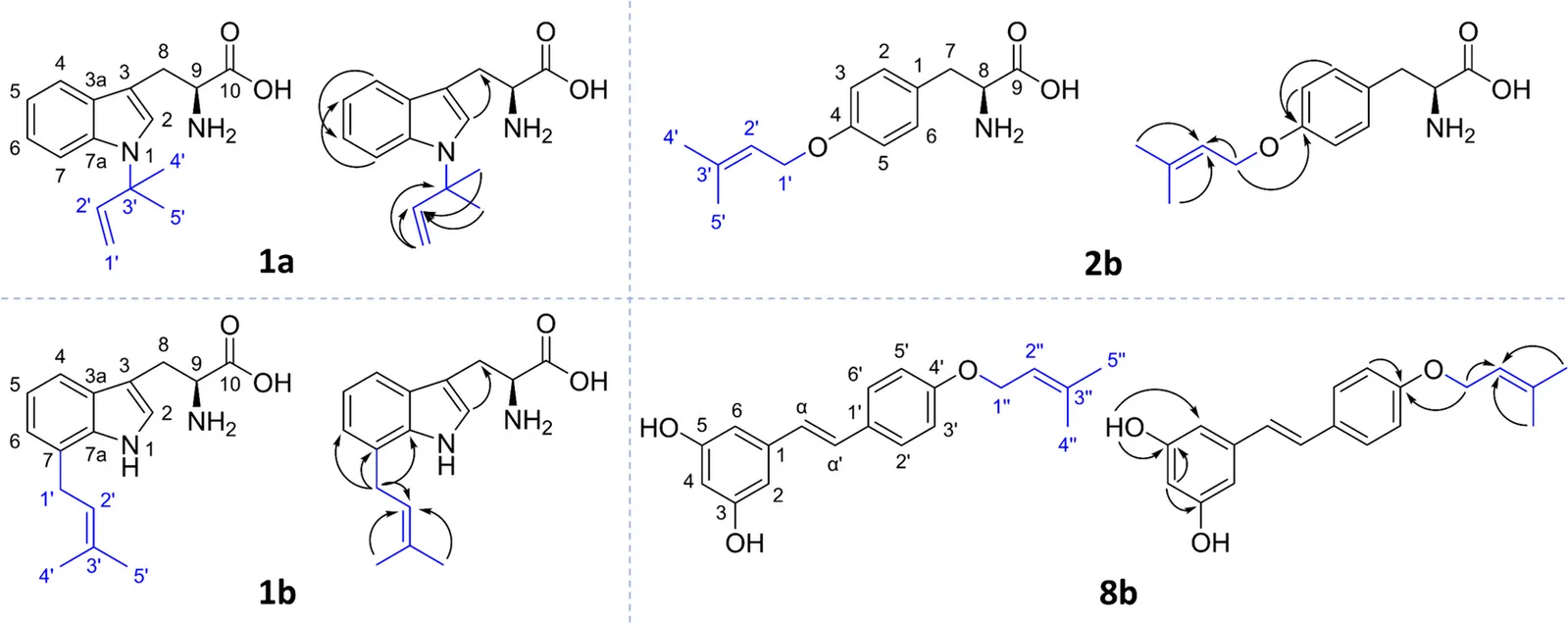
Chemical structures of selected prenylated products produced by RePT. Prenyl groups are shown in blue. Arrows indicate key HMBC correlations

^1^H-NMR, ^13^C-NMR, and two-dimensional heteronuclear multiple bond correlation (HMBC) and heteronuclear single quantum coherence (HSQC) NMR were used to determine the prenylation position and prenyl configuration of several main products generated by RePT (**1a**, **1b**, **2b**, and **8b**). The structures of these products and the key HMBC correlations used for their elucidation are shown in Fig. 4. The ^1^H and ^13^C NMR spectral data and all spectra are shown in Table S5 and Figs. S18–S29. The accurate mass of each product determined with FT-MS is shown in Table S6.

##### *C*7-prenyl-l-tryptophan (1b)

The main product from **1** (**1b**, 80% RA) showed only four peaks in ^1^H-NMR in the aromatic region (δ_H_ = 6.5–7.5 ppm), indicating that a prenyl moiety replaced an existing aromatic proton, thus hinting at *C*4–*C*7 or *C*2 prenylation. The four peaks consisted of a singlet, two doublets, and a triplet. The singlet was likely the *H*2 (CH, 7.43 ppm), as there were no neighboring protons and this proton had HMBC correlations with *C*3, *C*3a, *C*7a, and *C*8. This suggested that prenylation occurred at the aromatic ring of the indole moiety (*C*4–*C*7). Among the remaining unidentified three peaks, the doublet at δ_H_ = 7.57 ppm showed an HMBC correlation with *C*3, which is characteristic and exclusive for *H*4. The triplet was then assigned to *H*5, as it showed *ortho* coupling (*J* = 7.3 Hz) with the two adjacent protons, i.e., *H*4 and *H*6. Consequently, this suggested *C*7-prenylation which was further confirmed by HMBC correlations of *H*1′ with *C*6, *C*7, and *C*7a. The fact that the two protons at *C*1′ were in the same environment and showed HMBC correlations with *C*6 and *C*7a confirmed that the prenyl group was attached via the *C*1′ position (normal prenylation). Thus, we concluded that product **1b** was normal *C*7-prenyl-l-tryptophan.

##### *N*1-prenyl-l-tryptophan (1a)

The minor product from **1** (**1a**, 20% RA) was analyzed in a mixture with **1b** since a pure compound could not be obtained with the current purification method. Nevertheless, the spectral data was clear to confirm the prenylation at *N*1 position. Unlike **1b**, **1a** showed five protons in the aromatic region indicating either *N*1-prenylation or *C*3-prenylation. Different from the normal prenyl configuration in **1b** that had one doublet of the two protons of *H*1′, the spectrum of **1a** showed two doublets of *H*1′ (δ_H_ = 5.16 and 5.21 ppm) indicating that the two *H*1′ protons were in a different environment, which confirmed the reverse prenyl configuration. Although we could not observe HMBC correlations over *N*1, such as between *C*3′ and *H*2 or to other aromatic protons, all chemical shifts of ^1^H and ^13^C were in accordance with those of *N*1-prenyl-l-tryptophan reported by Burkhardt et al. (2019), which shows that *H*2 has an HMBC correlation with *C*3′, and by Rudolf and Poulter (2013). Therefore, it was concluded that **1a** was reverse *N*1-prenyl-l-tryptophan.

##### *O*4-prenyl-l-tyrosine (2b)

The ^1^H spectrum of **2b** showed four aromatic protons, suggesting that prenylation did not occur at aromatic carbons. Additionally, we observed a proton peak pattern and HMBC/HSQC correlations that are typical for normal prenyl configuration. Importantly, an HMBC correlation between *H*1′ and *C*4 was present. Thus, it was concluded that **2b** is *O*4-prenyl-l-tyrosine.

##### *O*4′-prenyl-resveratrol (8b)

A singlet of two hydroxyl protons (2H, δ_H_ = 9.20 ppm) was assigned to the hydrogen atom at the hydroxyl groups attached to *C*3 and *C*5. This consequently indicated that the *O*-prenylation expected based on our MS^2^ data should be at the only remaining hydroxyl group attached to *C*4′. This was further supported by disappearance of the peak of a hydroxyl proton attached to *C*4′ compared to the NMR data of non-prenylated resveratrol (δ_H_ = 9.52 ppm, data not shown). Furthermore, the key HMBC correlation between *H*1″ and *C*4′ was observed and therefore confirmed **8b** to be *O*4′-prenyl-resveratrol. All the chemical shifts of ^1^H and ^13^C were in accordance with *O*4′-prenyl-resveratrol reported by He et al. (2020), except for the peaks assigned to Hα and Hα′. From our spectrum, δ_H_ of Hα (6.89 ppm) was slightly smaller than Hα′ (6.97 ppm), which was in accordance with other sources reporting ^1^H spectra of resveratrol derivatives (Chen et al. 2017; de Bruijn et al. 2018).

#### Prenylation of aromatic amino acids

RePT showed a similar product pattern with **1** and **2**, with a main product at 80% RA and a minor product at 20% RA. This ability to catalyze prenylation at more than one site on **1** or **2** is not typically observed for most other characterized DMATSs, as they usually exhibit a strict regioselectivity. A few other DMATSs that do show similar behavior include SirD, which also catalyzes *C*7- and *N*1-prenylation of **1**, but forms only one product of **2** (i.e., **2b**), and CtrpPT which catalyzes *C*7- and *N*1-prenylation of cyclo-l-Trp-l-Trp, but does not accept **1** as substrate (Zou et al. 2010; Rudolf and Poulter 2013). The RA of **1a** and **1b** produced by RePT remained the same at different incubation times (2, 6, and 24 h) and enzyme concentrations (0.1 and 1 mg/mL) (Fig. S15a), suggesting the simultaneous prenylation at each of these two sites, as also reported for CtrpPT.

#### Prenylation of (plant) phenolics

For most of the substrates incubated with RePT, at least two products were formed. This behavior is similar to other DMATSs such as AcaPT, 7-DMATS_Afu_, and AnaPT that yield one major mono-prenylated product, and up to two minor products for a few substrates (Yu and Li 2011; Zhou et al. 2015; He et al. 2020). The occurrence of multiple product formation could originate from one binding orientation or from possible multiple binding sites or orientations within the active site of the enzyme. This possibility is supported by findings from AtaPT (Chen et al. 2017) where different residues were found involved in the binding of different (non-natural) substrates. An exception to the behavior of RePT to produce multiple products was substrate **32**, for which the incubation with RePT yielded just one product. Possibly the rigidity of this substrate allowed only one binding orientation in the active site of RePT.

#### RePT prefers *O*-prenylation of plant phenolics

To determine whether RePT performed *C*- or *O*-prenylation, we used the MS^2^ fragmentation patterns to tentatively identify the products. In short, *C*-prenylation yielded a major fragment corresponding to a neutral loss (NL) of 55 or 56 u from fragmentation between the prenyl *C*1′ and *C*2′ bond, whereas *O*-prenylation yields a major fragment corresponding to an NL of 68 u from the fragmentation of the ether bond between prenyl C1′ and an oxygen atom (Haagen et al. 2007; Simons et al. 2009; de Bruijn et al. 2018; van Dinteren et al. 2021). In this study, we used NLs of 68 or 69 u to tentatively identify *O*-prenylated products and NLs of 55 u (in case of stilbenes), 56 or 57 u for *C*-prenylated products (Table S3). Additionally, *O*-prenylated products eluted later in RP-UHPLC compared to *C*-prenylated products of the same substrate, which is in accordance with their more hydrophobic nature. Note that this guideline was not applicable to identify prenylated amino acids, as no main fragments with the indicated NLs (i.e., 56/57 u, 68/69 u) were observed in MS^2^. For **5** and **25**, the type of prenylation of the main product could not be confidently identified, but at least one minor product was *O*-prenylated. Still, using this approach, we determined that the main prenylated plant phenolics produced by RePT were *O*-prenylated (substrate **6**–**8**, **10**, **14**–**15**, and **32**) (Fig. 3).

#### Product profile steered by divalent cations and enzyme concentration

Interestingly, we observed a product profile change influenced by divalent cations and enzyme concentration, which to the best of our knowledge, has not been reported before for other DMATSs. A lower ratio of enzyme to substrate led to higher regioselectivity with **2**, yielding one product instead of two (Fig. S15c). The addition of divalent cations also reduced the formation of the minor product of **2**, when using 1 mg/mL RePT, from 13% (without divalent cations) to 7% (with Ca^2+^) and 0% (with Mg^2+^). However, the product profile did not change for substrate **1** with a different ratio of enzyme to substrate or for **8** with divalent cations (Fig. S15a–b). Although the effect of enzyme-to-substrate ratio and the addition of divalent cations on the product profile was not consistent among substrates thus far, these results hint at an opportunity to steer the prenylated product profile by adjusting the reaction condition.

## Discussion

In the last two decades, over 30 DMATSs have been reported to catalyze prenylation of indole derivatives and other aromatic compounds. In this study, we report a novel DMATS RePT from *Rasamsoni emersonii*, which utilizes DMAPP to mono-prenylate aromatic amino acids at two positions, or to mainly mono-*O*-prenylate stilbenes and selected other phenolics. Similar to other DMATSs that often show a distinct preference for either aromatic amino acids or cyclic dipeptides (CDPs) (Grundmann and Li 2005; Kremer et al. 2007; Steffan and Li 2009; Mundt and Li 2013), RePT showed a clear preference for aromatic amino acids. Previous studies suggest that this preference could either be attributed to the arginine residue located at two positions downstream of the strictly conserved RXKXY motif, which is conserved in amino acid-converting DMATSs but absent in CDP-converting DMATSs, as suggested by Burkhardt et al. (2019), or to another arginine that is located in a similar position in the active site (Fan and Li 2016). This arginine likely contributes to amino acid substrate binding, by forming a hydrogen bond with the carboxylic side chain of the amino acids as evident in DMATS1_Ff_ (Eaton et al. 2022), an amino acid-converting DMATS. This arginine residue is also found in RePT (R290), further supporting arginine’s proposed role in steering the substrate scope towards amino acids rather than CDPs, and illustrating the use of this residue for predicting the DMATS substrate scope.

Although RePT preferred small flexible substrates like aromatic amino acids and stilbenes, some bulkier and more rigid molecules, such as tricyclic (iso)flavonoids and tetracyclic coumestan, were still converted to a certain extent. Conformational changes in the active site to accommodate bulky substrates might underly this promiscuity of RePT, similar to what was speculated from modeling and experimental evidence with NotF (Kelly et al. 2022). Furthermore, its distinct preference for stilbenes over other bulkier phenolic substrates distinguished RePT from other promiscuous DMATSs, such as AtaPT and AcaPT, which accept various bulkier phenolic backbones. The higher acceptance of bulkier phenolic substrates by AtaPT likely comes from the ability of AtaPT to form multiple hydrophobic interactions between the substrates and the enzyme, as observed by Chen et al. (2017) (Fig. S8). On the other hand, the prenyl acceptor binding region of RePT is less hydrophobic and more charged (Fig. S6), which may explain the preference of RePT for less bulky phenolic substrates.

Regarding its product profile, RePT prenylates l-tryptophan (**1**) at both *C*7 and *N*1, which is uncommon among DMATSs, which typically yield only one product from aromatic amino acids. Burkhardt et al. (2019) suggested that an orientation of DMAPP in parallel to **1** can facilitate both a normal *C*7-prenylation and a reverse *N*1-prenylation (in case of SirD). This is due to the close proximity of *C*1 in DMAPP to *C*7 in **1**, and *C*3 in DMAPP to *N*1 in **1**. A docking study of MpnD, a bacterium aPT catalyzing indolactam V, in complex with GPP and indolactam V showed a similar distance between *C*5 of the acceptor and *C*1 of GPP and between *C*7 of the acceptor and *C*3 of GPP, explaining its normal *C*5- and reverse *C*7-prenylation (Mori et al. 2016). Therefore, prenylation of aromatic amino acids at two different positions by RePT might originate from the same substrate binding mode.

From a structural perspective, the active site architecture of RePT in our model shows rather similar substrate-binding residues (i.e., F111, M112, E120) to those of DMATS1_Ff_, which is an *N*1 tryptophan-prenylating enzyme (Fig. S8). This indicates that the substrate binding of RePT, particularly towards l-tryptophan and l-tyrosine, may adopt almost identical interactions. The latter is substantiated by the shown formation of *O*4-prenyl-l-tyrosine by both enzymes. For l-tryptophan, however, RePT showed a preferential formation of *C*7 over *N*1, while DMATS1_Ff_ mainly resulted in *N*1-prenylation, which hint at a slightly different orientation of l-tryptophan in these enzymes. The factors driving RePT’s preference for *C*7-prenylation over its minor reaction, *N*1-prenylation, remain unclear.

Most DMATSs have a preference towards either *C*- or *O*- prenylation on phenolics. It can be hypothesized that for the *O*-prenylation, the mechanism might be similar to the one suggested for *N*-prenylation of indole substrate. The crystal structure of DMATS1_Ff_ shows that the conserved residue E90 coordinates *N*1 of **1** and *O*4 of **2**, corresponding to the enzyme’s actual prenylation position (Eaton et al. 2022). This is in accordance with another study, which proposed that this coordination may increase *N*1 nucleophilicity, making it more readily available to form a bond with carbocation intermediates, before getting deprotonated by the same residue (Burkhardt et al. 2019). It seems plausible that the enzymes that catalyze *O*-prenylation utilize the similar mechanism towards hydroxyl groups of phenolic substrates. Still, published indole *N*1-prenylating enzymes are not strictly *O*-prenylating phenolics: AstPT (reverse *N*1- and *C*2-prenylating DMATS) strictly *O*-prenylates hydroxyxanthones, whereas CdpNPT (*N*-prenylating DMATS) prefers *C*-prenylation of hydroxynapthalenes (Yu et al. 2011; Tarcz et al. 2014). The observation that AnaPT (reverse *C*3-prenylating DMATS) produced mixed products with a higher ratio of *O*-prenylated products compared to CdpNPT on the same substrates within the same study (Yu et al. 2011) further elaborates the discrepancy. 7-DMATS_Afu_, the closest DMATS to RePT in terms of sequence identity (44%), produces mainly *C*7-prenyl-**1**, and catalyzes *C*-prenylation of (iso)flavonoids and a dihydrochalcone **10** (Yu and Li 2011; Zhou et al. 2015). Hence, not only the prenylation mechanism but likely also substrate binding mode will play a role in determining the preference of RePT and other DMATSs for *O*- versus *C*-prenylation.

RePT demonstrates the potential to modulate product profiles through varying reaction conditions, such as enzyme concentrations and cation introduction, though only observed with **2**. The introduction of MgCl_2_ enhanced the regioselectivity of RePT towards **2b** (Fig. S12c). Although not conclusive, this result may hint at the cation’s influence on the substrate’s orientation in the active site, which has been previously reported for AmbP1, a cyanobacterial aPT (Awakawa et al. 2018). AmbP1 mainly catalyzes *C*2-geranylation of *cis*-indolyl vinyl isonitrile but in the presence of Mg^2+^, it mainly catalyzes *C*3-geranylation instead (Liu et al. 2016). Awakawa et al. (2018) show that this change was due to conformational changes of the aromatic acceptor-binding residues upon cation binding.

In conclusion, this study describes a novel DMATS-type fungal aromatic prenyltransferase, dubbed RePT. RePT is a valuable addition to the DMATS family as a tool to prenylate tryptophan, tyrosine, and diverse phenolic substrates. In particular, its ability to perform *O*-prenylation of phenolics complements the limited ability of previously reported DMATSs to perform *O*-prenylation of phenolic substrates. Finally, our results hint that l-tryptophan, which was the best substrate among those tested, could be RePT’s natural substrate. The efficient conversion of the two aromatic amino acids that play a central role in the biosynthesis of fungal natural products encourages a future investigation on prenylation of their closely related derivatives, which could open possibilities to create building blocks for synthesizing diverse biologically active compounds.

## Supplementary Information

Below is the link to the electronic supplementary material.

Supplementary file1 (PDF 5.27 MB)

## Data Availability

Data will be made available on request.
