# Supplementary material for: Prenylation of aromatic amino acids and plant phenolics by an aromatic prenyltransferase from Rasamsonia emersonii
Source: Appl Microbiol Biotechnol. 2024 Jul 18;108:421. doi: 10.1007/s00253-024-13254-8 (PMC11258057; doi:10.1007/s00253-024-13254-8)
Supplement: Supplementary file 1 [file 253_2024_13254_MOESM1_ESM.pdf]

Supplementary information

Prenylation of aromatic amino acids and plant phenolics by an aromatic prenyltransferase from *Rasamsonia emersonii*

Pimvisuth Chunkrua[1], Kai P. Leschonski[1], Alejandro A. Gran-Scheuch[2], Gijs J. C. Vreeke[1], Jean-Paul Vincken[1], Marco W. Fraaije[2], Willem J. H. van Berkel[1], Wouter J. C. de Bruijn[1], Mirjam A. Kabel[1]*

[1] Laboratory of Food Chemistry, Wageningen University, Bornse Weilanden 9, 6708 WG Wageningen, The Netherlands
[2] Molecular Enzymology Group, University of Groningen, Nijenborgh 4, 9747 AG Groningen, The Netherlands

* Corresponding author: Mirjam A. Kabel
Telephone: +31 (0)317 483209
Email: mirjam.kabel@wur.nl

Supplementary tables

**Table S1**	Aromatic substrates used in the substrate screening	4
**Table S2**	Nucleotide and amino acid sequences of His-SUMO-RePT, and primer sequences used in gene sequencing	6
**Table S3**	Substrate conversion, UV and MS data, and the tentative identification of enzymatic products from RePT reactions in the presence of DMAPP and well-accepted substrates	20
**Table S4**	Substrate recovery (%) of aromatic substrates well-accepted by RePT	25
**Table S5**	NMR data of **1a** and **1b**, **2b**, and **8b**	26
**Table S6**	ESI-FT-MS data of the purified prenylated products produced using RePT	39

Supplementary figures

**Fig. S1-3**	The results of using 3,4,5-trimethoxycinnamic acid (TMCA) as an internal standard for RePT reaction	7
**Fig. S4**	Nucleotide and amino acid sequences of His-SUMO-RePT, and primer sequences used in gene sequencing	8
**Fig. S5**	A structural model of untagged RePT acquired using AlphaFold2 colored according to pLDDT score and the order of amino acids	10
**Fig. S6**	Structural models of an N-terminal truncated AtaPT in an apo state and the untagged RePT acquired using AlphaFold2	11
**Fig. S7**	An expanded view of the active site architecture of RePT	12
**Fig. S8**	An expanded view of the active site architecture of four DMATSs	13
**Fig. S9**	SDS-PAGE analysis of the purified His-SUMO-RePT	14
**Fig. S10**	MS spectrum of intact His-SUMO-RePT	14
**Fig. S11**	Quantitative coverage of amino acids after peptide analysis of His-SUMO-RePT digested with porcine trypsin and *Bacillus licheniformis* protease	15
**Fig. S12**	Peptides released upon digestion of RePT hydrolyzed by porcine trypsin and BLP	15
**Fig. S13**	Substrate conversion of **1** and resveratrol **8** by RePT in the presence of DMAPP at three pHs	16
**Fig. S14**	Substrate conversion of **1** by RePT in the presence of DMAPP throughout 24-hours incubation at different reaction conditions: two different enzyme concentrations/methanol content/buffers/pHs	16
**Fig. S15**	Substrate conversion (%) and relative abundance of prenylated products (%) by RePT in the presence of DMAPP and **1**, **2**, or **8** with different reaction conditions (enzyme concentration, additives, time points)	17
**Fig. S16**	Substrate conversion of **1** converted by RePT when using different concentrations of **1** (0.1-20 mM)	17
**Fig. S17**	UHPLC-UV chromatograms of enzymatic reactions of RePT towards well-accepted substrates in the presence of DMAPP	18
**Fig. S18 – S20**	NMR spectra of **1a**	27
**Fig. S21 - S23**	NMR spectra of **1b**	30
**Fig. S24 - S26**	NMR spectra of **2b**	33
**Fig. S27 - S29**	NMR spectra of **8b**	36
**Fig. S30**	Experimental data involved in DMAPP synthesis	40

Supplementary methods

Synthesis of dimethylallyl pyrophosphate tri-ammonium salt (DMAPP)	39
Upscaled production of prenylated compounds	40

Table S1 Aromatic substrates used in substrate screening for RePT. Compounds accepted with > 10 % conversion, 1-10 % conversion, and < 1% conversion are shown in green, blue, and grey, respectively.

No.	Group	Compound	CAS no.	Purity (%)	Supplier [a]
1	Amino acids	L-Tryptophan	73-22-3	≥98	Sigma-Aldrich
2		L-Tyrosine	60-18-4	≥98	Sigma-Aldrich
3	Cyclic dipeptides	Cyclo(L-Trp-L-Trp)	20829-55-4	≥98	Cayman Chemical
4		Cyclo(L-Trp-L-Pro),	38136-70-8	≥98	Cayman Chemical
5	Stilbenes	Oxyresveratrol	29700-22-9	≥98	Cayman Chemical
6		Piceatannol	10083-24-6	>98	Cayman Chemical
7		Pinostilbene	42438-89-1	>97	Tokyo Chemical
8		Resveratrol	501-36-0	≥99	Sigma-Aldrich
9		Pinosylvin	22139-77-1	≥97	Sigma-Aldrich
10	Dihydrochalcones	Phloretin	60-82-2	≥99	Sigma-Aldrich
11	Flavonoids: Chalcones	Isoliquiritigenin	961-29-5	>98	Tokyo Chemical
12		Cardamonin	19309-14-9	≥98	Sigma-Aldrich
13		Xanthohumol	6754-58-1	≥99	Carl Roth
14	Flavonoids: Flavanols	(+)-Catechin	225937-10-0	>97	Tokyo Chemical
15		(-)-Epicatechin	490-46-0	>97	Tokyo Chemical
16	Flavonoids: Flavanonols	Taxifolin	480-18-2	99	Selleckchem
17	Flavonoids: Flavonols	Kaempferol	520-18-3	≥99	Extrasynthese
18		Quercetin	849061-97-8	95	Thermo Scientific
19	Flavonoids: Flavanones	Naringenin	67604-48-2	>93	Tokyo Chemical
20		Sakuranetin	520-29-6	≥95	Sigma-Aldrich
21		Eriodictyol	4049-38-1	≥99	Extrasynthese
22	Flavonoids: Flavones	Apigenin	520-36-5	≥99	INDOFINE Chemical
23		Luteolin	491-70-3	≥98	Extrasynthese
24		Baicalein	491-67-8	98	Sigma-Aldrich
25	Isoflavonoids: Isoflavans	(±)-Equol	94105-90-5	≥98	Cayman Chemical
26		Glabridin	59870-68-7	≥98	Sigma-Aldrich
27	Isoflavonoids: Isoflavones	Daidzein	486-66-8	≥95	Cayman Chemical
28		Genistein	446-72-0	≥98	Cayman Chemical
29		Glycitein	40957-83-3	≥98	FUJIFILM Wako Chemicals Europe
30		Formononetin	485-72-3	≥99	Sigma-Aldrich
31		Biochanin A	491-80-5	≥95	Sigma-Aldrich
32	Isoflavonoids: Coumestans	Coumestrol	479-13-0	≥95	Sigma-Aldrich
33	Benzopyrones: Coumarins	Esculetin	305-01-1	≥95	Sigma-Aldrich
34	Benzophenones	4-hydroxybenzophenone	1137-42-4	98	Sigma-Aldrich
35	Phenolic amides	Avenanthramide A	108605-70-5	≥98	Sigma-Aldrich
36		Avenanthramide B	108605-69-2	≥98	Sigma-Aldrich
37		Avenanthramide C	116764-15-9	≥98	Sigma-Aldrich
38	Hydroxybenzoic acid and methoxylated derivatives	4-Hydroxybenzoic acid	99-96-7	99	Sigma-Aldrich
39		Vanillic acid	121-34-6	97	Sigma-Aldrich
40		Syringic acid	530-57-4	≥95	Sigma-Aldrich
41	Cinnamic acids and aldehyde derivatives	*trans*-Cinnamic acid	140-10-3	≥99	Sigma-Aldrich
42		*p*-Coumaric acid	501-98-4	≥98	Sigma-Aldrich
43		*trans*-Ferulic acid	537-98-4	≥99	Sigma-Aldrich

No.	Group	Compound	CAS no.	Purity (%)	Supplier [a]
44		Sinapic acid	530-59-6	≥98	Sigma-Aldrich
45		3,4,5-Trimethoxycinnamic acid	90-50-6	>98	Tokyo Chemical
46	Monolignols	Coniferyl alcohol	458-35-5	98	Sigma-Aldrich
47		Sinapyl alcohol	537-33-7	80	Sigma-Aldrich
48	Lignin (related) dimers	Guaiacylglycerol-β-guaiacyl	7382-59-4	>97	Tokyo Chemical
49		Veratrylglycerol-β-guaiacyl	10535-17-8	97	abcr Gute Chemie
50		Hydrocoerulignone	25496-72-4	95	MP Biomedicals

[a] abcr Gute Chemie (Karlsruhe Germany), Carl Roth (Karlsruhe, Germany), Cayman Chemical (Ann Arbor, MI, USA), Extrasynthese (Genay, France), FUJIFILM Wako Chemicals Europe (Neuss, Germany), INDOFINE Chemical (Hillsborough, NJ, USA), MP Biomedicals (Irvine, CA, USA), Selleckchem (Houston, TX, USA), Sigma-Aldrich (St. Louis, MO, USA), Thermo Scientific Chemicals (Waltham, MA, USA), and Tokyo Chemical Industry (TCI Europe) (Zwijndrecht, Belgium).

Table S2 Nucleotide and amino acid sequences of His-SUMO-RePT, and primer sequences used in gene sequencing. The sequence of the SUMO-tag is represented as **bold text**, and the sequence of RePT is represented as underlined text.

	**Sequence**
Codon-optimized nucleotide sequence	ATGGGCAGCAGCCATCATCATCATCATCACGGCAGCGGCCTGGTGCCGCGCGGCAGC<br>GCTAGC**ATGTCGGACTCAGAAGTCAATCAAGAAGCTAAGCCAGAGGTCAAGCCA**<br>**GAAGTCAAGCCTGAGACTCACATCAATTTAAAGGTGTCCGATGGATCTTCAGAG**<br>**ATCTTCTTCAAGATCAAAAAGACCACTCCTTTAAGAAGGCTGATGGAAGCGTTC**<br>**GCTAAAAGACAGGGTAAGGAAATGGACTCCTTAAGATTCTTGTACGACGGTATT**<br>**AGAATTCAAGCTGATCAGACCCCTGAAGATTTGGACATGGAGGATAACGATATT**<br>**ATTGAGGCTCACAGAGAACAGATTGGTGGT**ATGGCACCTGGCAGTCATTCCACAG<br>CGAACTTGACTCATGCGACCGACAGCGCATCCCTGTTGCCGCCTGTTGCAGCCCGCC<br>CTGCCACTTCCTTAGACAATAGCGTGTATCATTCACTCACCTCAGTTCTGGAGTTCGC<br>CGATCAAGATCAACTTTTCTGGTGGACCGAGACAGCCTCGATTGTTGCGAAACTGAT<br>GGAAAGCGCCGGTTACGACGTGGAGTCACAACGTCGCTATCTGCTGCTTTACCACGC<br>ACACATCCTGGCCGCATTAGGTCCCAAGCAGACGCCGTGTCCGTCCGACCGCTCCGC<br>CAGTTCGCCATGGAAGTCTTTTATGACAGACGACCATACTCCCATTGAGATCAGCTG<br>GAATCTTGGGGCCAATCGCTCTGTGGTGCGCCTGTCGATTGAGCCTATTGGTCCTTTC<br>GCTGGTACCAGCTTTGATCCGTTCAACCAGCGCCCCGCGCTTGAGCTTCTCCAACAGT<br>TGCCAGGTATCGACCTGCAACTGTTTTACTACTTCCGTGACTGGTTCTTCATTGATGA<br>CGACGACGTCGACGACGTACTTAAGCGCCGTCCTGCTGGTGAGCATAGCTCGCAACT<br>CTTTGTAGCTTTCGACTTTGATGGTGGCAAGGTTACGACCAAGGCGTACATTTTCCCG<br>CTCTTAAAGGCACTTGAAACCGGAATTCCGGTCTTGGACTTGGTTAGCACGGCGATC<br>CGCAATTTAGATGAGCCAGCGTTATCCGTCAGCCCTGGCTGGAACGTACTCGAGGAC<br>TTCATTCGCAGTTGCCCGATTGCGTCACGTCCTAAGCTGGAATTTATCGCGATCGACT<br>GTGTCGCGCCTGAGAAGTCCCGCATCAAAATCTACGTACGCACACCCCATACGGCTT<br>TAGAGAAAGTTAAGGATGTATTTACTCTTGGTGGCCGCTTAAATGACCAAACCATCC<br>AAACCGCATTGGGCATGCTCGAGGAGCTTTGGCGCCTCGTCCTCGACCTGCCCGACG<br>GGTTACGCGATAGTGATGAGTTGCACCCTCGCGACGAGAATTCAGCGGGGCATCGTA<br>CGAGCGGAGTATTGTTTAATTTCGAGATCAAGCCAGGCGCCGCGCTGCCGGAACCTA<br>AATTATACATCCCAGTCCGTCATTACGCGCGTAGTGACCTCGATATTGCACGCGGCTT<br>GACCGCGTTCTTCCGTCGTCGTGGTTGGACATCCCTGGCGGAGACTTATACTGACAC<br>CTTAAAAGAGACGTTCCCGCACCATCCTCTGGCGGAGTCTACCAGTACTCACACGTA<br>TATCGTCTTTGCCTTTAAGAAGACCACAGGGGTGTATTTAACTGCGTATTATAATCCT<br>CAGGTGTATGCGCGTCCCAAGCCGAGTGAGAAGTTGGAGTGGACGAAGGACCGTCT<br>GCGCGGACATCGTCTTTGA
Amino acid sequence confirmed by peptide analysis	GSSHHHHHHGSGLVPRGSAS**MSDSEVNQEAKPEVKPEVKPETHINLKVSDGSSEIFFK**<br>**IKKTTPLRRLMEAFAKRQGKEMDSLRFLYDGIRIQADQTPEDLDMEDNDIIEAHRE**<br>**QIGG**MAPGSHSTANLTHATDSASLLPPVAARPATSLDNSVYHSLTSVLEFADQDQLFW<br>WTETASIVAKLMESAGYDVESQRRYLLLYHAHILAALGPKQTPCPSDRSASSPWKSFMT<br>DDHTPIEISWNLGANRSVVRLSIEPIGPFAGTSFDPFNQRPALELLQQLPGIDLQLFYYFRD<br>WFFIDDDDVDDVLKRRPAGEHSSQLFVAFDFDGGKVTTKAYIFPLLKALETGIPVLDLVS<br>TAIRNLDEPALSVSPGWNVLEDFIRSCPIASRPKLEFIAIDCVAPEKSRIKIYVRTPHTALEK<br>VKDVFTLGGRLNDQTIQTALGMLEELWRLVLDLPDGLRDSDELHPRDENSAGHRTSGV<br>LFNFEIKPGAALPEPKLYIPVRHYARSDLDIARGLTAFFRRRGWTSLAETYTDTLKETFPH<br>HPLAESTSTHTYIVFAFKKTTGVYLTAYYNPQVYARPKPSEKLEWTKDRLRGHRL
Primer (5'-3')	ATGCCATAGCATTTTTATCC
Primer (3'-5')	GGACTATGTCTAATTTAG

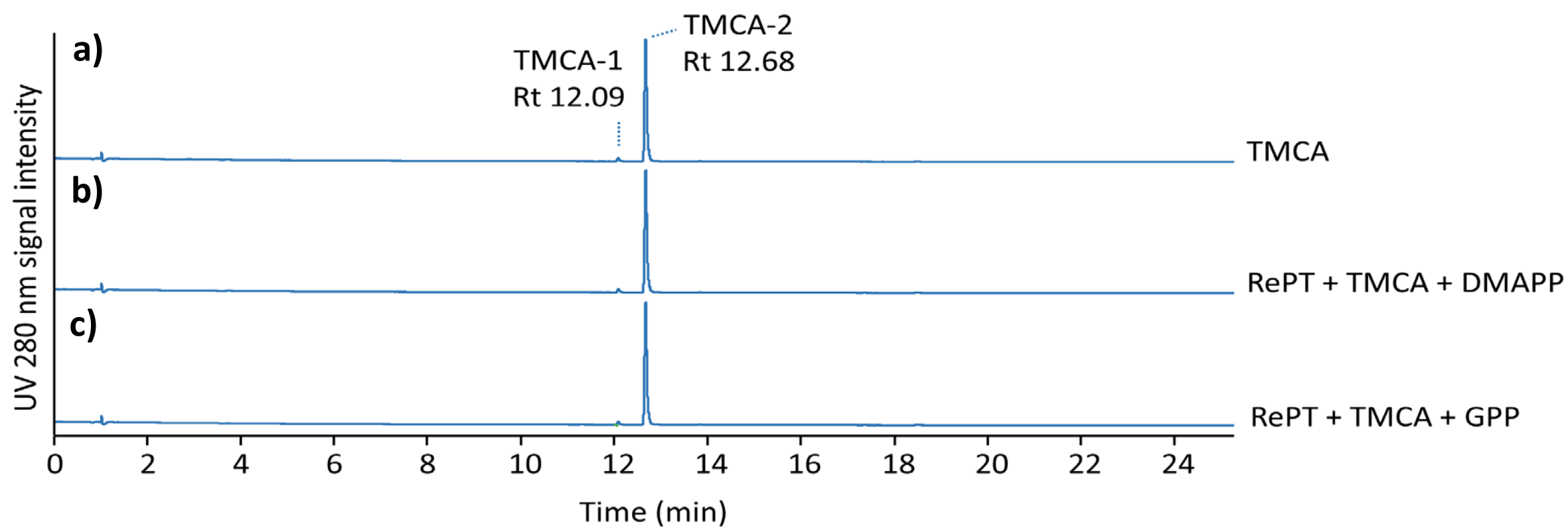

Fig. S1 UHPLC-UV chromatograms of **a)** 3,4,5-trimethoxycinnamic acid (TMCA) isomers (TMCA-1 is *cis*-TMCA and TMCA-2, is *trans*-TMCA). The peak area ratio TMCA-2:TMCA-1 is 39:1. **b)** Incubation of RePT with TMCA in the presence of DMAPP and **c)** of GPP. No product formation was observed in both reactions.

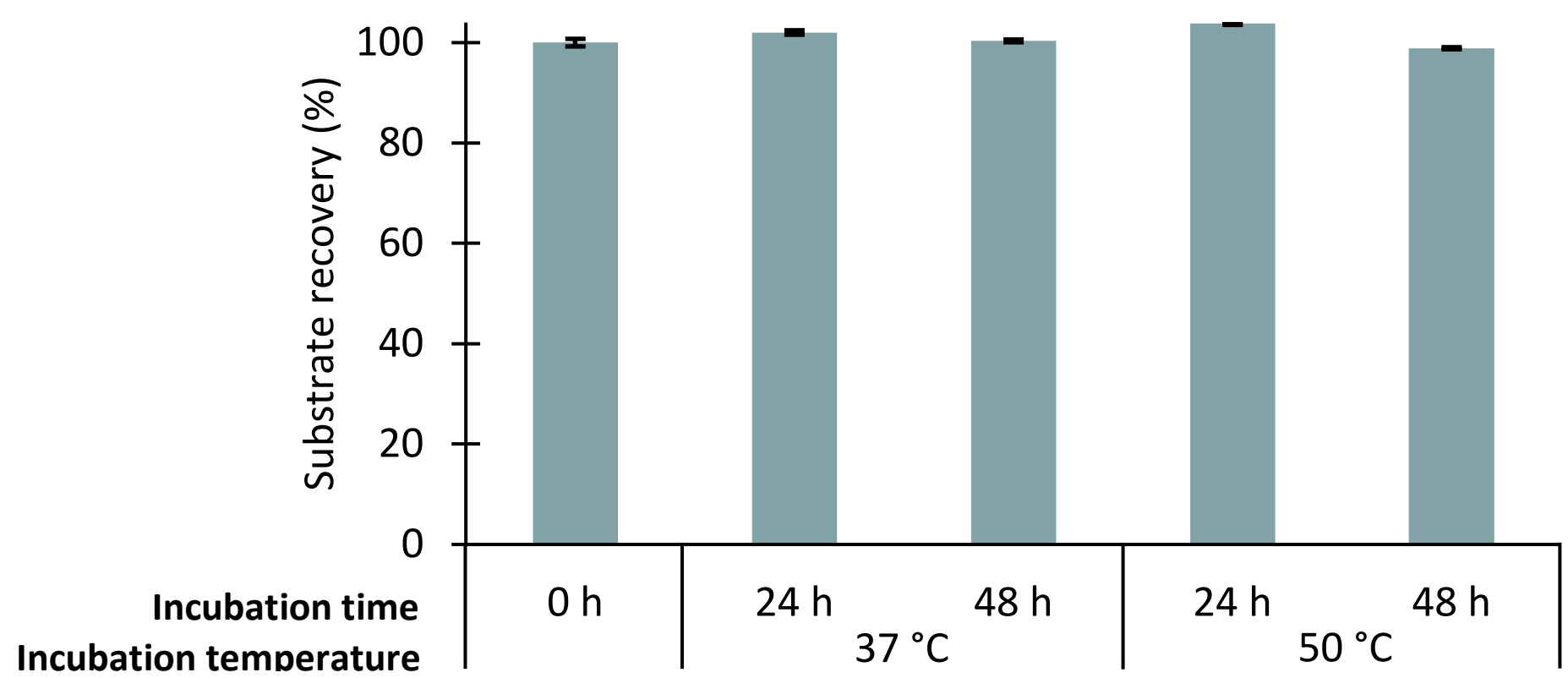

Fig. S2 Recovery of an aromatic reference compound TMCA-2 (*trans*-TMCA) tested at two temperatures (37 °C and 50 °C), up to 48 incubation hours. The recovery was calculated and reported in relative to a UV280 nm peak area of TMCA without incubation. The peak area of TMCA-1 was significantly smaller than TMCA-2, with less than 2.5% of the sum of the peak area of both isomers in all conditions. Therefore, the recovery was shown based on TMCA-2 only.

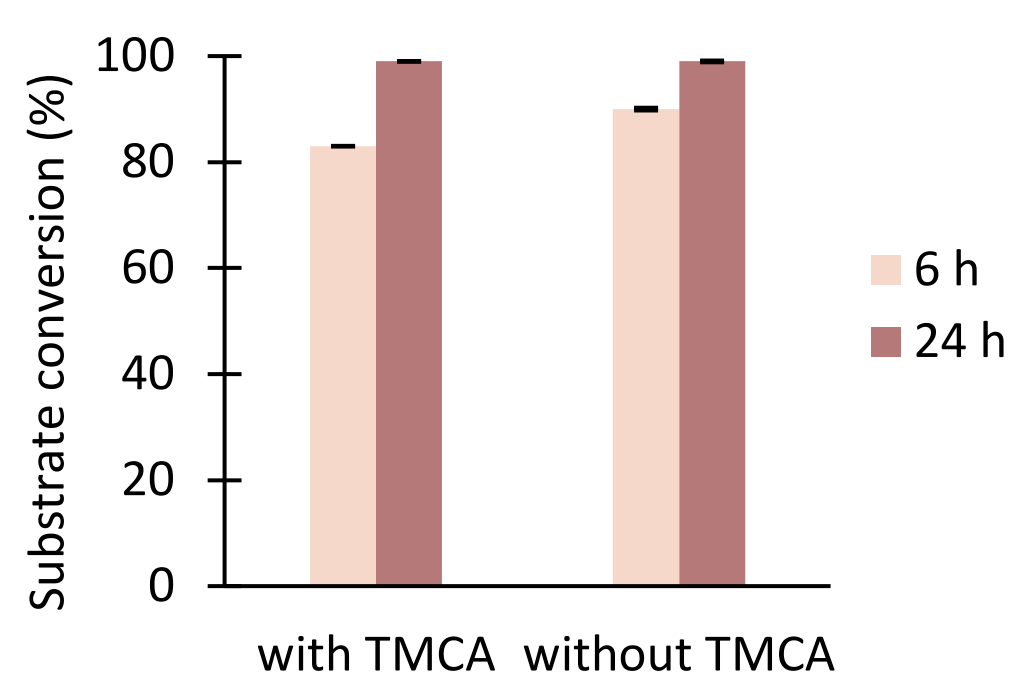

Fig. S3 Comparison of the conversion (%) of **1** by RePT in the presence of DMAPP, with or without 0.16 mM TMCA, after incubation for 6 and 24 hours at 37 °C. The concentration of RePT used in this experiment was 0.1 mg/mL, ten times lower than the concentration used to characterize the substrate scope of RePT.

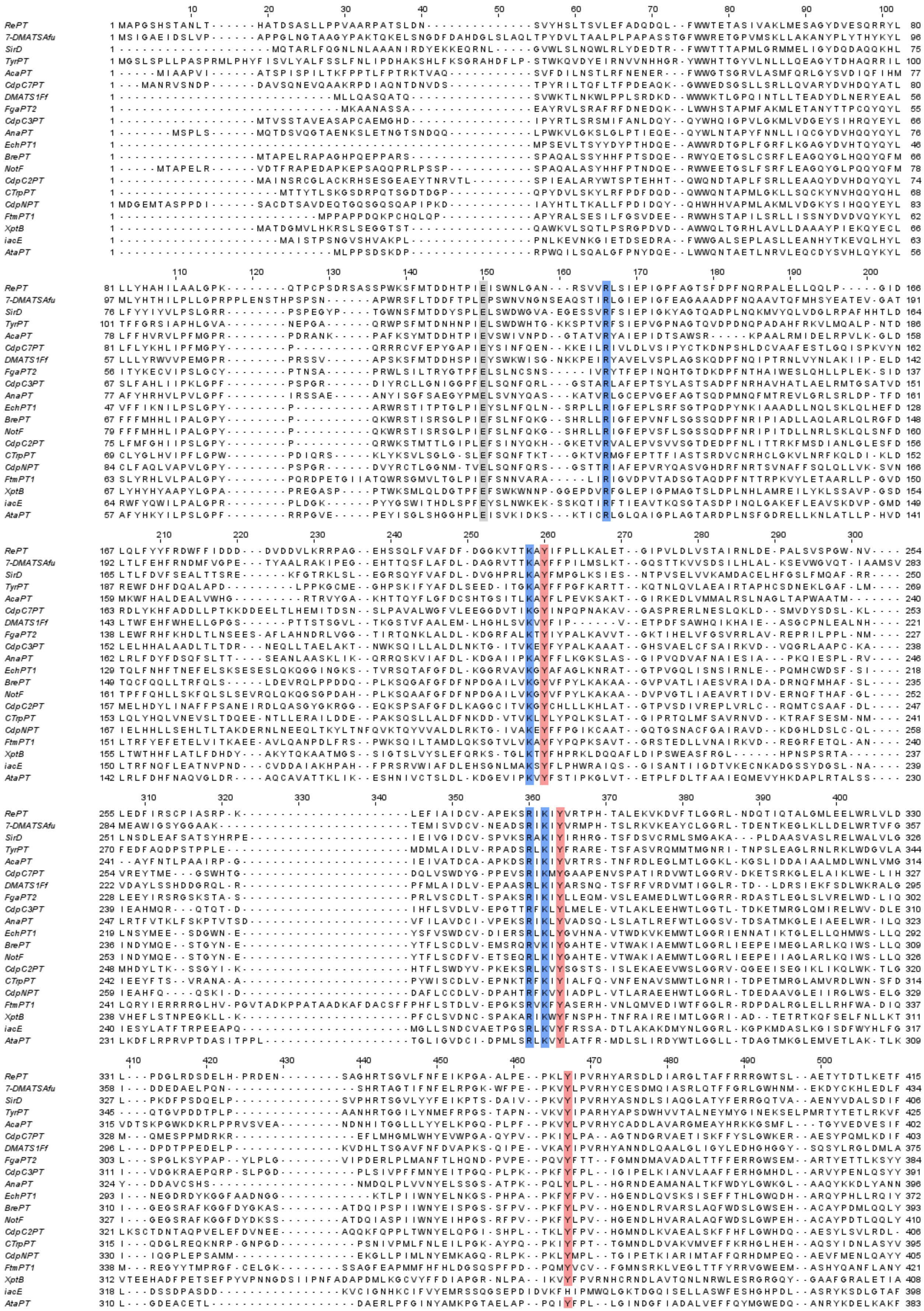
RePT
7-DMATSAfu
SirD
TyrPT
AcaPT
CdpC7PT
DMATS1Ff
FgaPT2
CdpC3PT
AnaPT
EchPT1
BrePT
NotF
CdpC2PT
CTrpPT
CdpNPT
FtmPT1
XptB
iacE
AtaPT

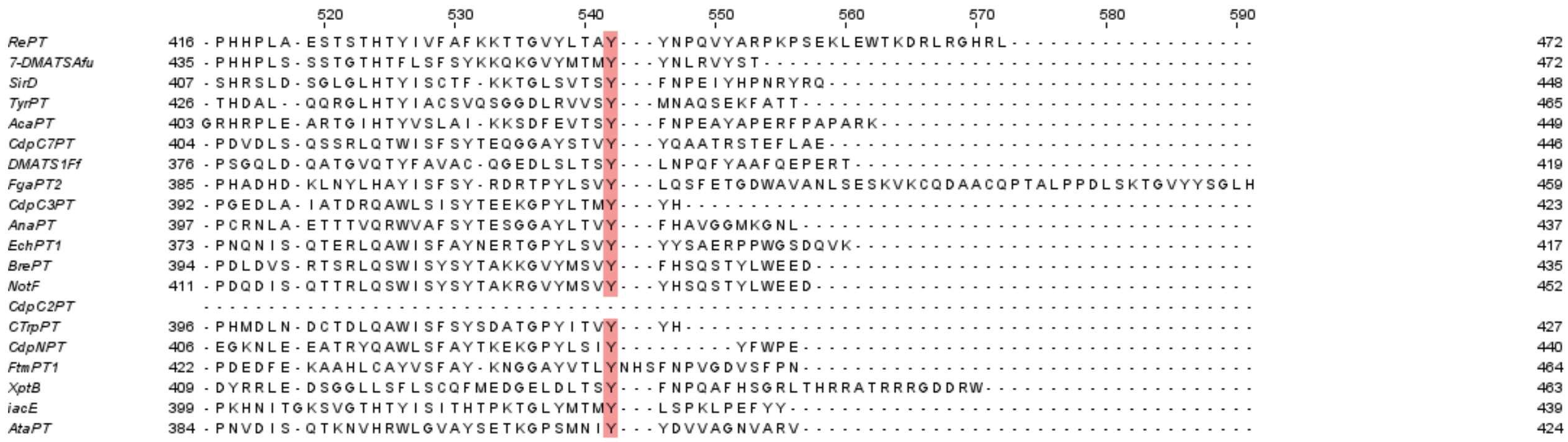

Fig. S4 Multiple sequence alignment analysis of RePT and 19 characterized DMATSs. The conserved key residues for prenyl donor binding are highlighted in blue, the conserved tyrosine residues for stabilizing the dimethylallyl carbocation intermediates in pink, and a key glutamate residue for binding the acceptor substrate in grey. Accession numbers of 20 DMATSs are as follow: RePT (XP_013322739.1), 7-DMATS$_{Afu}$ (XP_754328.2), SirD (XP_003842411.1), TyrPT (XP_001396322.1), AcaPT (QHD39928.1), CdpC7PT (XP_001213396.1), DMATS1$_{Ff}$ (XP_023436063.1), FgaPT2 (AAX08549.1), CdpC3PT (XP_001259405.1), AnaPT (XP_001258078.1), EchPT1 (ATP76206.1), BrePT (AFM09725.1), NotF (ADM34132.1), CdpC2PT (XP_001267443.1), CTrpPT (ADI60056.1), CdpNPT (ABR14712.1), FtmPT1 (AAX56314.1), XptB (P0DP82.1), iacE (A0A1J0HSL6.1), and AtaPT (AMB20850.1).

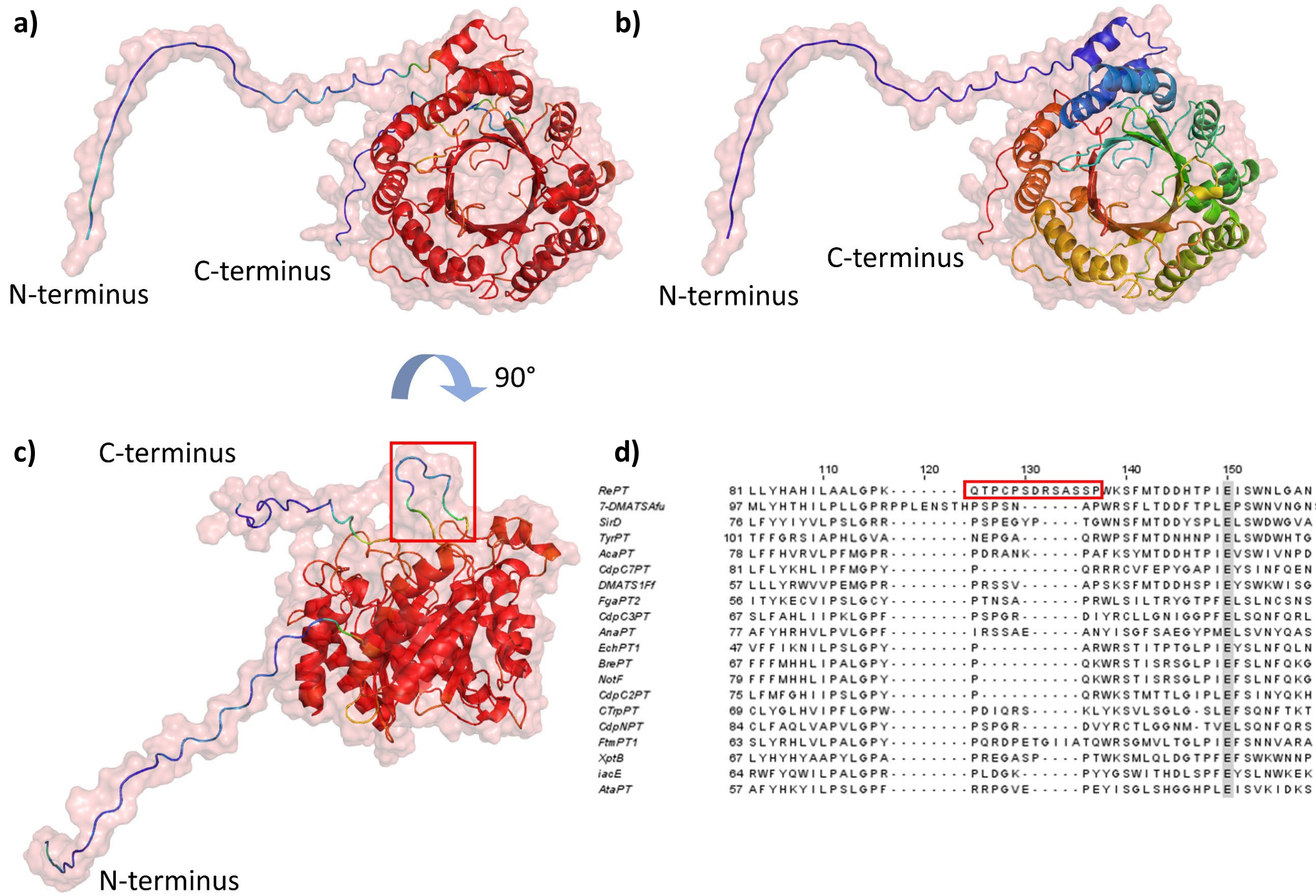

Fig. S5 A structural model of untagged RePT acquired using AlphaFold2. The model is colored according to **a)** the predicted local distance difference test (pLDDT) score of the AlphaFold2 model, with high and low confidence scores shown in red and blue, respectively, and **b)** the order of amino acids in which the *N*-terminus is colored blue and C-terminus is colored red. **c)** the view of **a)** with 90° rotation on X-axis to display the area with low pLDDT score (in the red rectangle). **d)** According to the multiple sequence alignment, the sequences in this area vary in amino acid composition and length among DMATSs. The structural models presented here were generated in PyMOL.

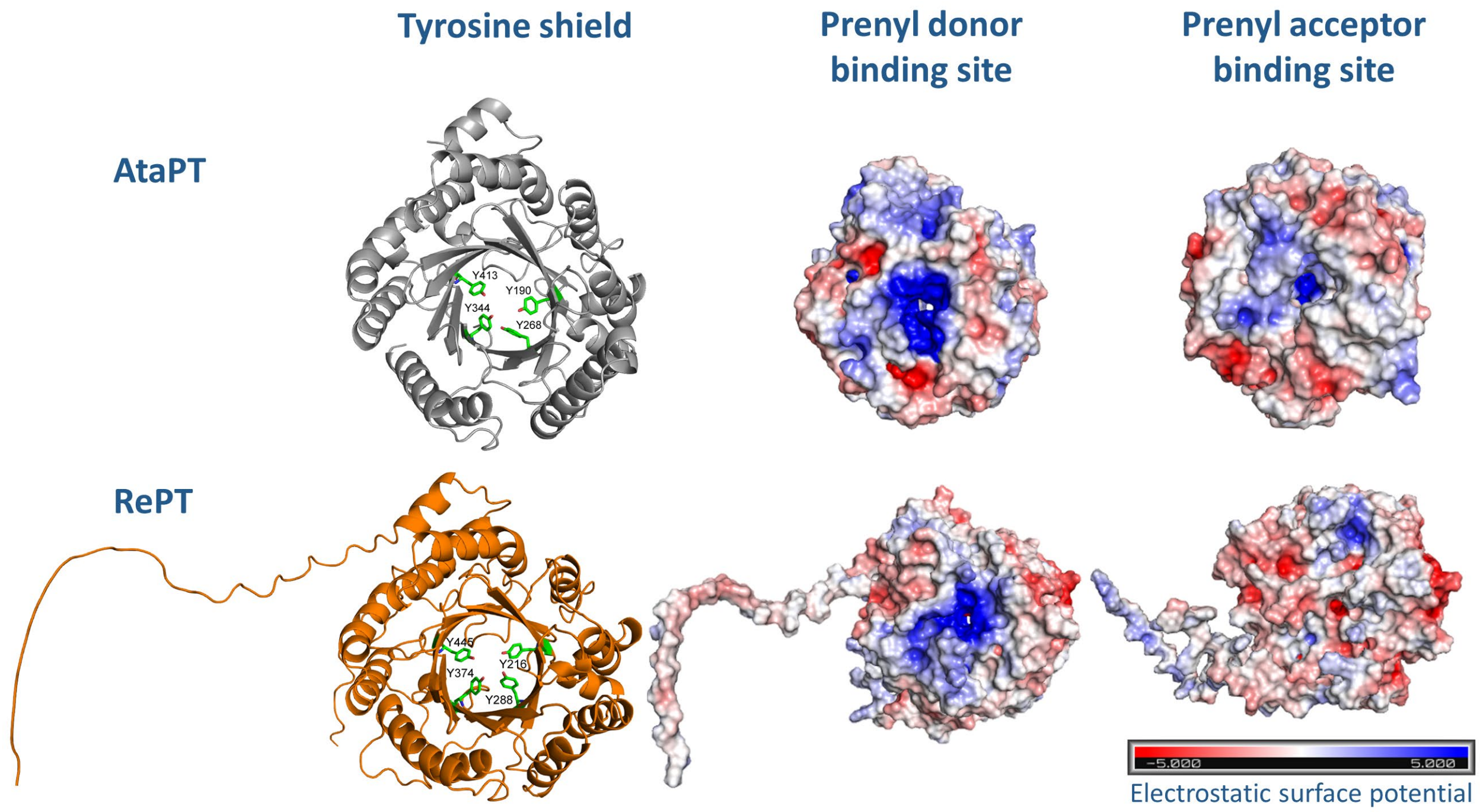

Fig. S6 Comparison of the structural models of *N*-terminal truncated AtaPT in the apo state (PDB: 5KCG) and the untagged RePT acquired using AlphaFold2. Residues in green represent the four highly conserved tyrosine residues (also known as the tyrosine shield). The electrostatic surface (positively charged in blue, negatively charged in red) of AtaPT displays a prenyl donor binding site (highly positively charged region) and an aromatic acceptor binding pocket (a more hydrophobic region) as reported by Chen et al. (2017).

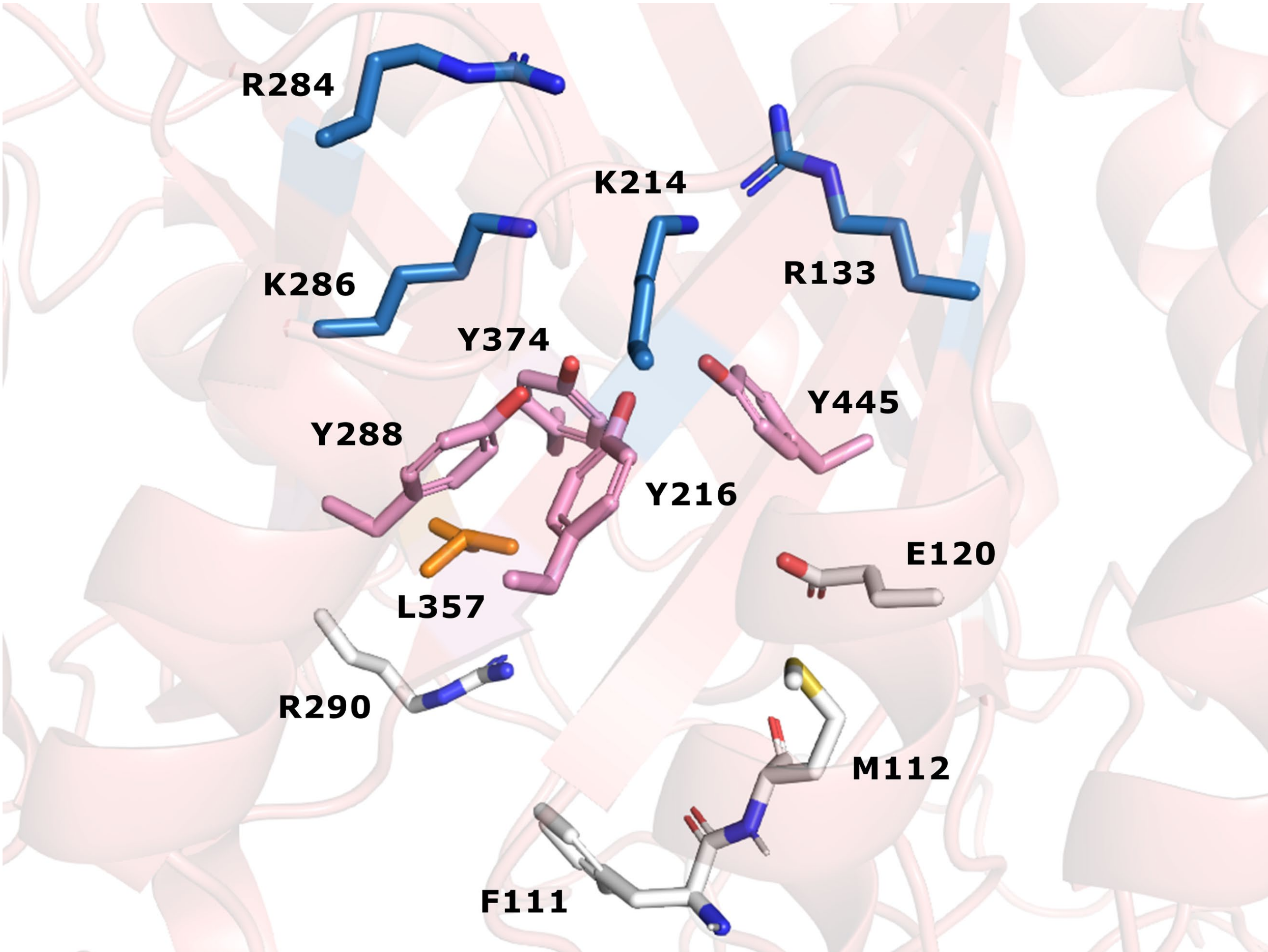

Fig. S7 An expanded view of the active site architecture of RePT using a model acquired with AlphaFold2. Possible key residues are highlighted: prenyl-donor binding (blue), carbocation-intermediate stabilizer (pink), acceptor-binding (white), and a possible determinant for prenyl donor specificity (orange). The proposed roles of the residues are based on Metzger et al. (2009), Chen et al. (2017), Burkhardt et al. (2019), and Eaton et al. (2022).

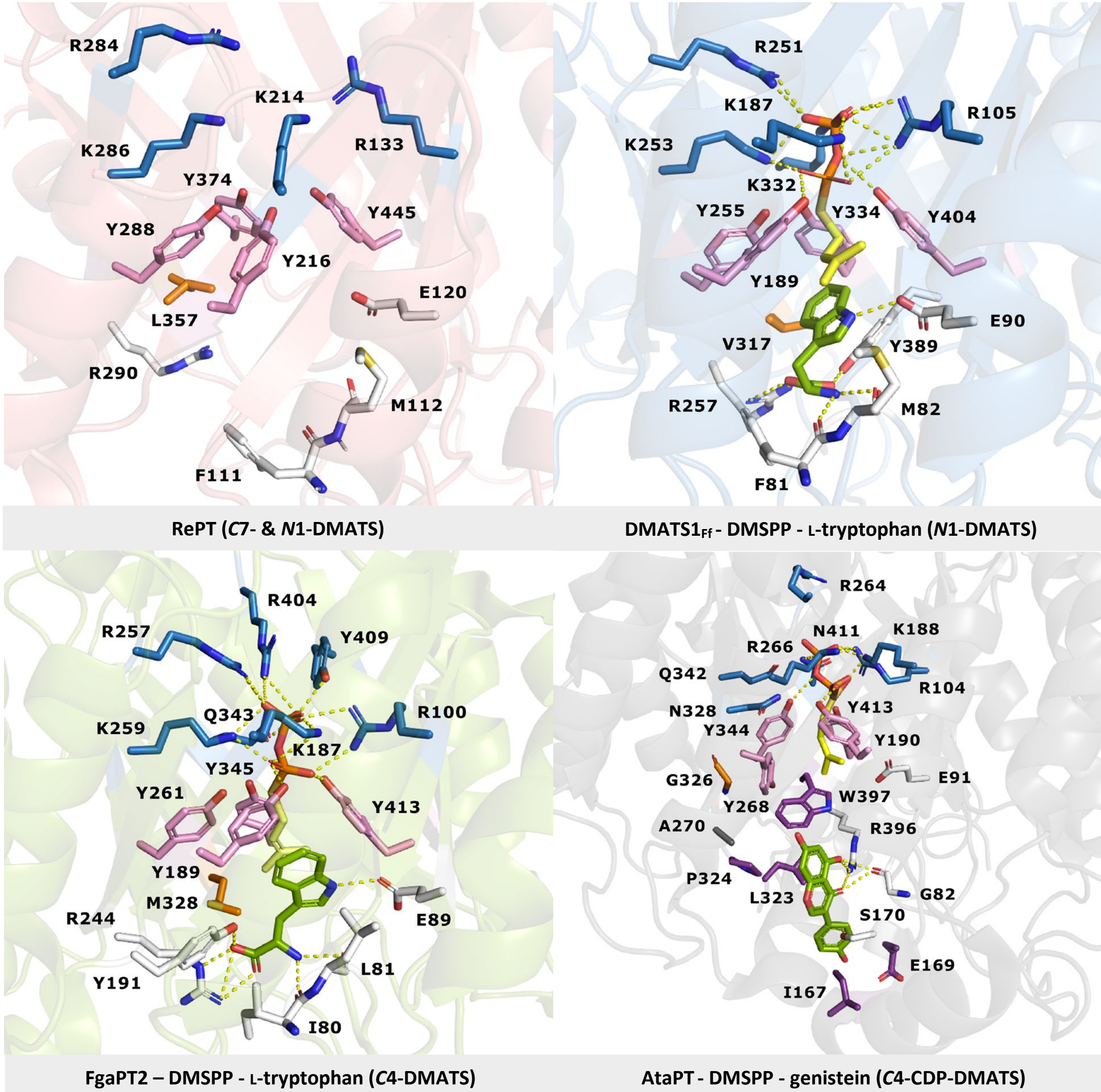

Fig. S8 An expanded view of the active site architecture of four DMATSs based on an AlphaFold2 model of RePT and crystal structures of DMATS1$_{Ff}$ (PDB: 8DB0), FgaPT2 (PDB: 3I4X), and AtaPT (PDB: 5KDA) bound with a prenyl donor analogue (DMSPP) (in yellow) and an aromatic acceptor (in green). Key residues and analogues of the key residues identified from other relevant DMATSs are highlighted: prenyl-donor binding (blue); carbocation-intermediate stabilizer (pink); acceptor-binding (white); non-ligand residues involved in hydrophobic interaction (purple); an analogue of amino-acid converting DMATSs' arginine two positions downstream of the conserved RXKXY motif (R290 in RePT, R257 in DMATS1$_{Ff}$, R244 in FgaPT2), proposed to determine the preference of the enzyme between aromatic amino acid and cyclic dipeptide (CDP) (gray); a possible determinant for prenyl donor specificity (orange). Yellow dashed lines represent possible hydrogen bonds. The proposed roles of the residues in the crystal structures are based on Metzger et al. (2009), Fan and Li (2016), Chen et al. (2017), Burkhardt et al. (2019), and Eaton et al. (2022).

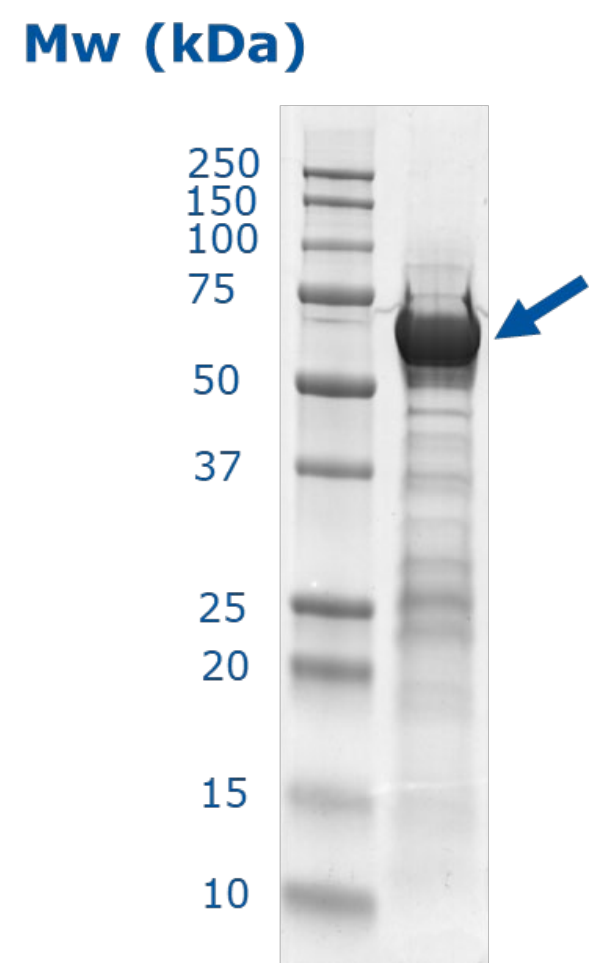

Fig. S9 SDS-PAGE analysis of the purified His-SUMO-RePT.

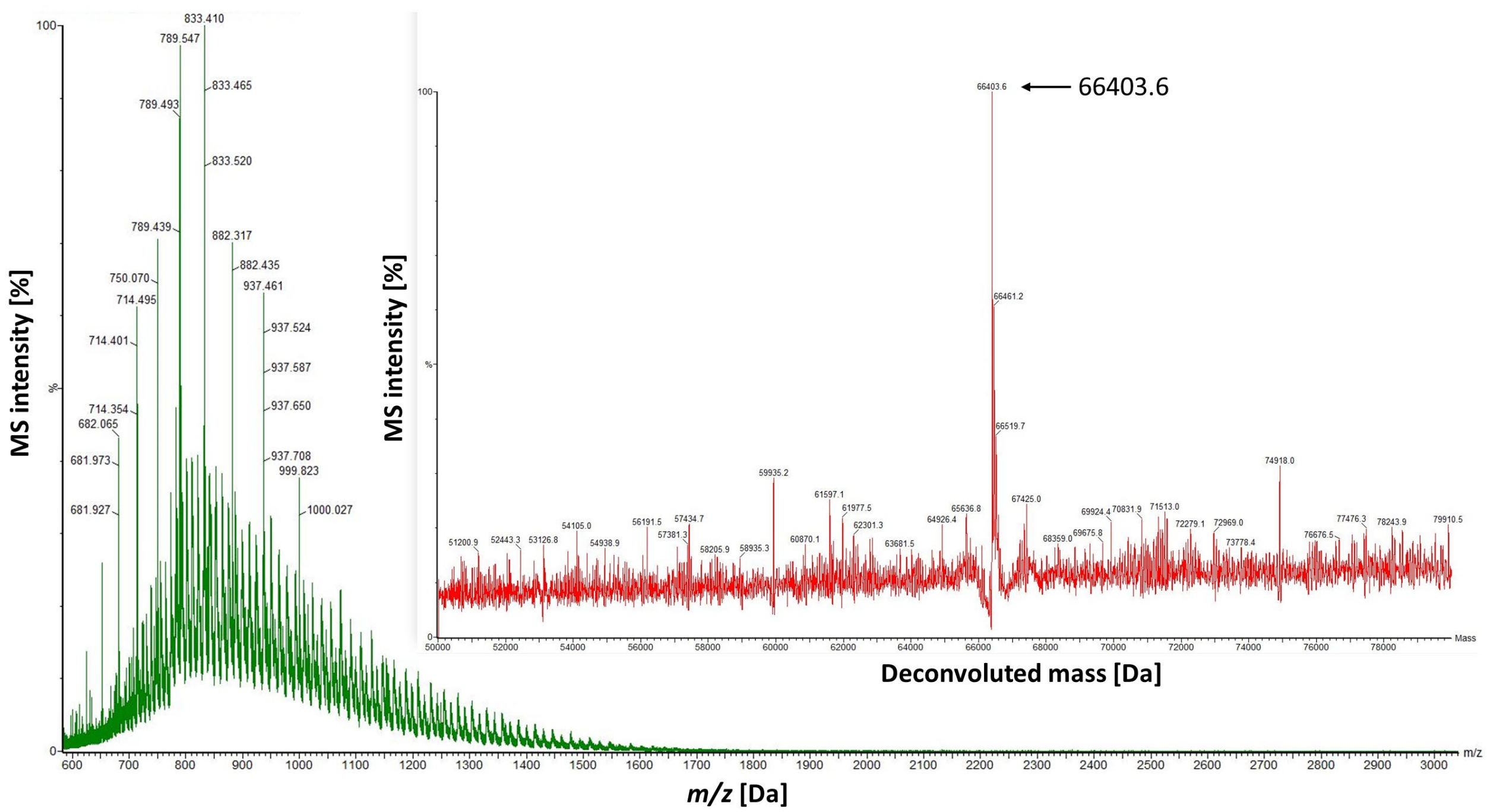

Fig. S10 MS spectrum of intact His-SUMO-RePT (sequence shown in **Table S1**) before and after deconvolution using Maxent.

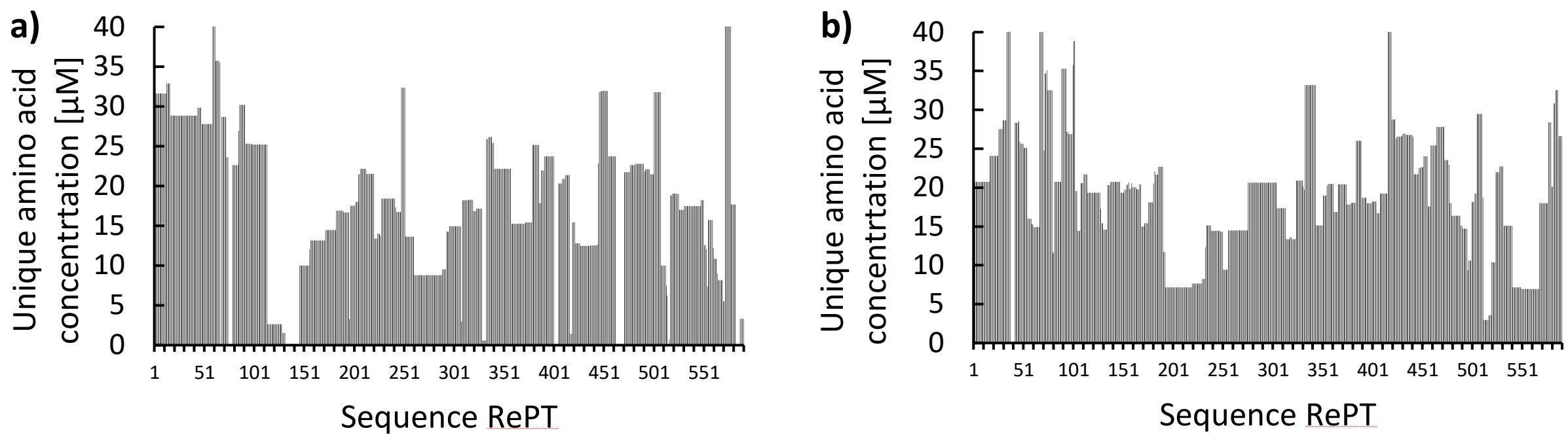

Fig. S11 Quantitative coverage of amino acids after peptide analysis of His-SUMO-RePT digested with porcine trypsin and *Bacillus licheniformis* protease (BLP). The average (unique) amino acid concentrations were 19 µM and 20 µM in RePT hydrolysates by porcine trypsin and BLP, respectively.

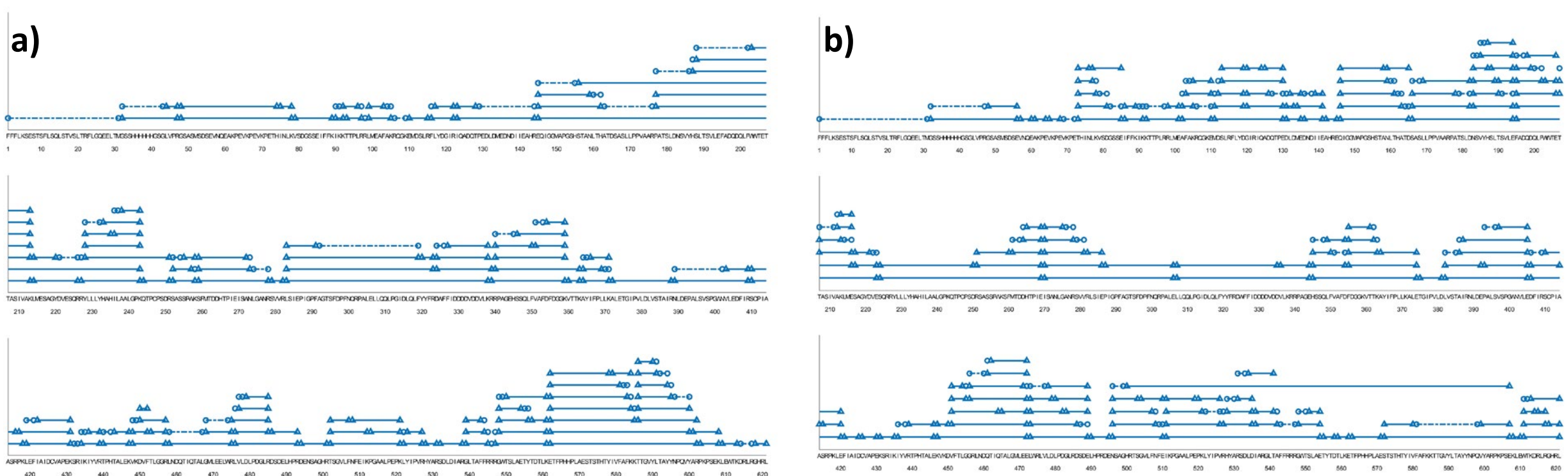

Fig. S12 Peptides released upon digestion of RePT hydrolyzed by **a)** porcine trypsin and **b)** BLP. The dotted lines indicate expected peptides that were not identified

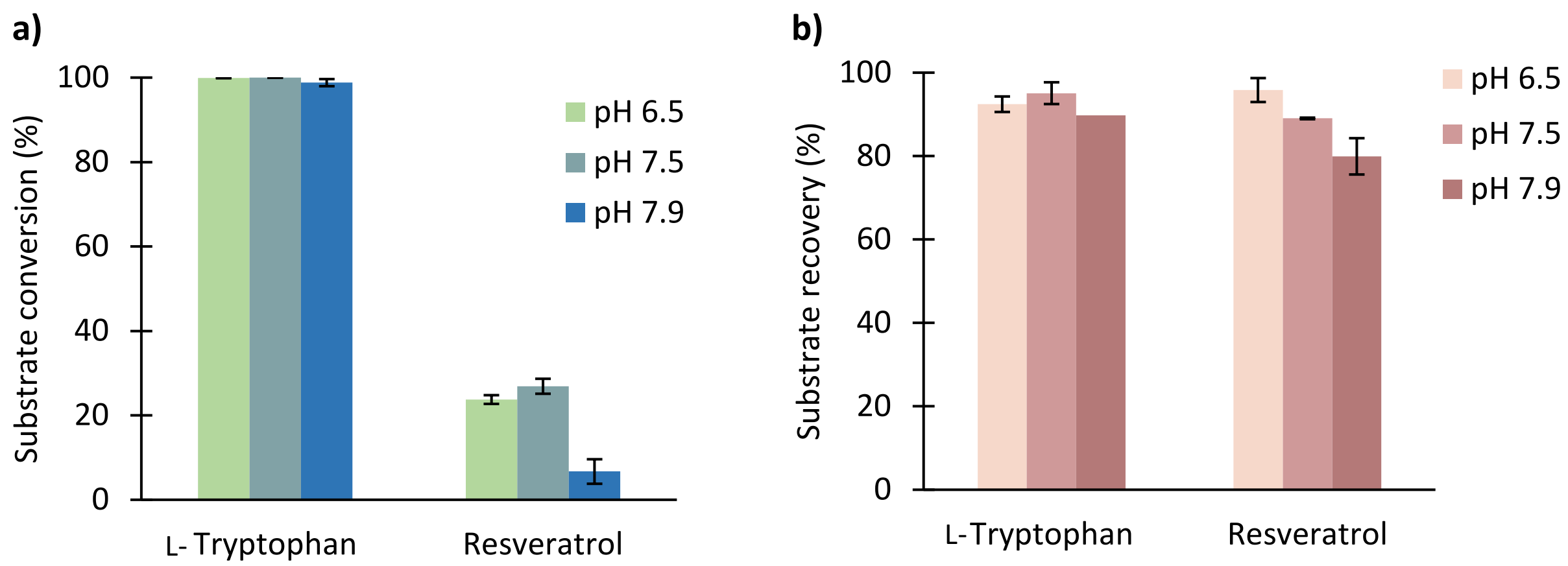

Fig. S13 a) Substrate conversion of L-tryptophan (**1**) and resveratrol (**8**) by RePT (1 mg/mL) in the presence of DMAPP at three pH values ranging from 6.5-7.9. The buffers used were 50 mM Bis-Tris/HCl (pH 6.5) and 50 mM Tris/HCl (pH 7.5 and 7.9) **b)** Substrate recovery after 24-hours incubation of **1** and **8** in the absence of RePT. The incubations were performed at a pH ranging from 6.5 to 7.9 in the presence of DMAPP. No clear pH optimum was observed when using **1**, but the highest conversion was observed at pH 7.5 when using **8**. The conversion of **8** was reduced to one-fourth at pH 7.9 compared to pH 7.5. This reduction could potentially be due to the lower stability of the substrate at high pH, as demonstrated by the lower substrate recovery at higher pH shown in **b)**. Due to the potential stability loss of other aromatic substrates at high pH as observed with **8**, pH 6.5 and 7.5 were then selected for further experiment to determine an optimum condition (**Fig. S11**). This experiment was performed without using TMCA as an internal standard.

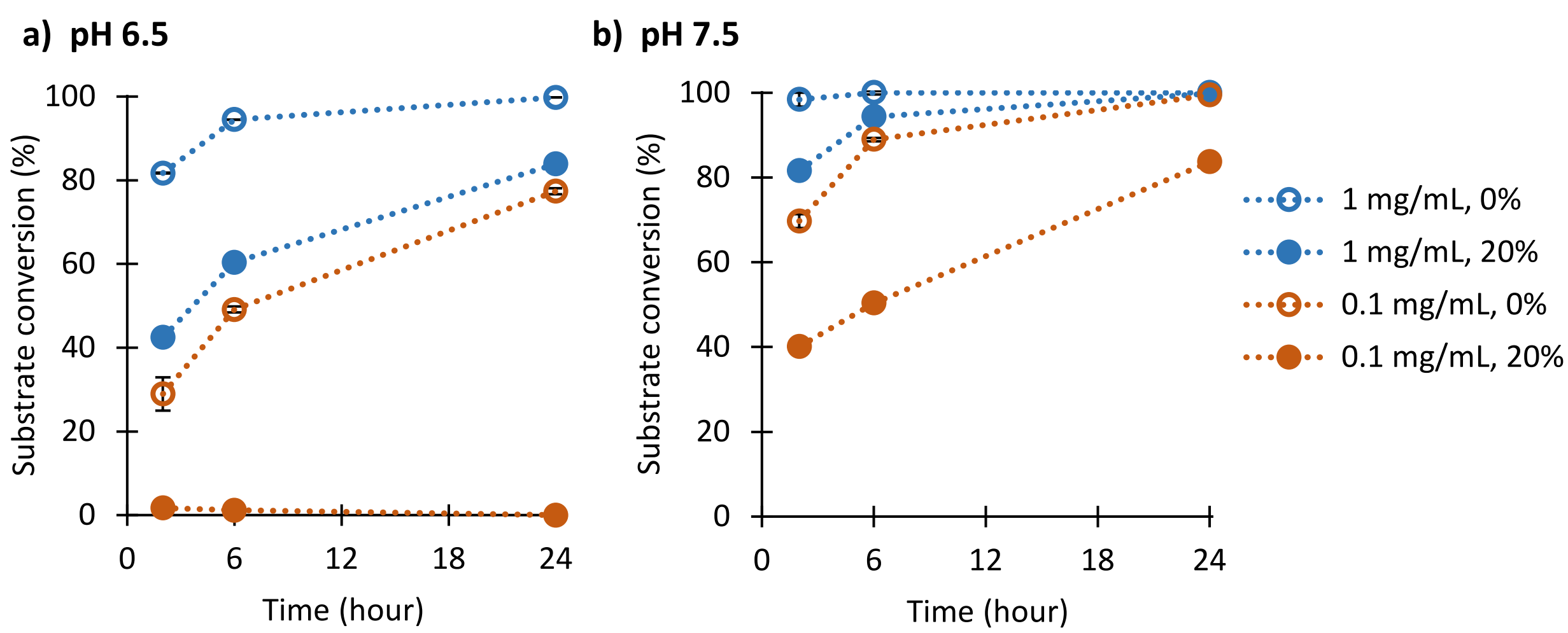

Fig. S14 Substrate conversion of **1** by RePT in the presence of DMAPP throughout 24-hours incubation at different reaction conditions: RePT concentration 1 or 0.1 mg/mL, 0 % or 20 % (*v/v*) methanol, and the buffer **a)** 50 mM Bis-Tris/HCl buffer, pH 6.5 or **b)** 50 mM Tris/HCl, pH 7.5. At a lower enzyme concentration (0.1 mg/mL) or with 20 % (*v/v*) organic solvent, the conversion of **1** at pH 6.5 was lowered and was completely absent when all three suboptimal conditions were combined. Therefore, pH 7.5 was selected as an optimum condition for the enzymatic reaction.

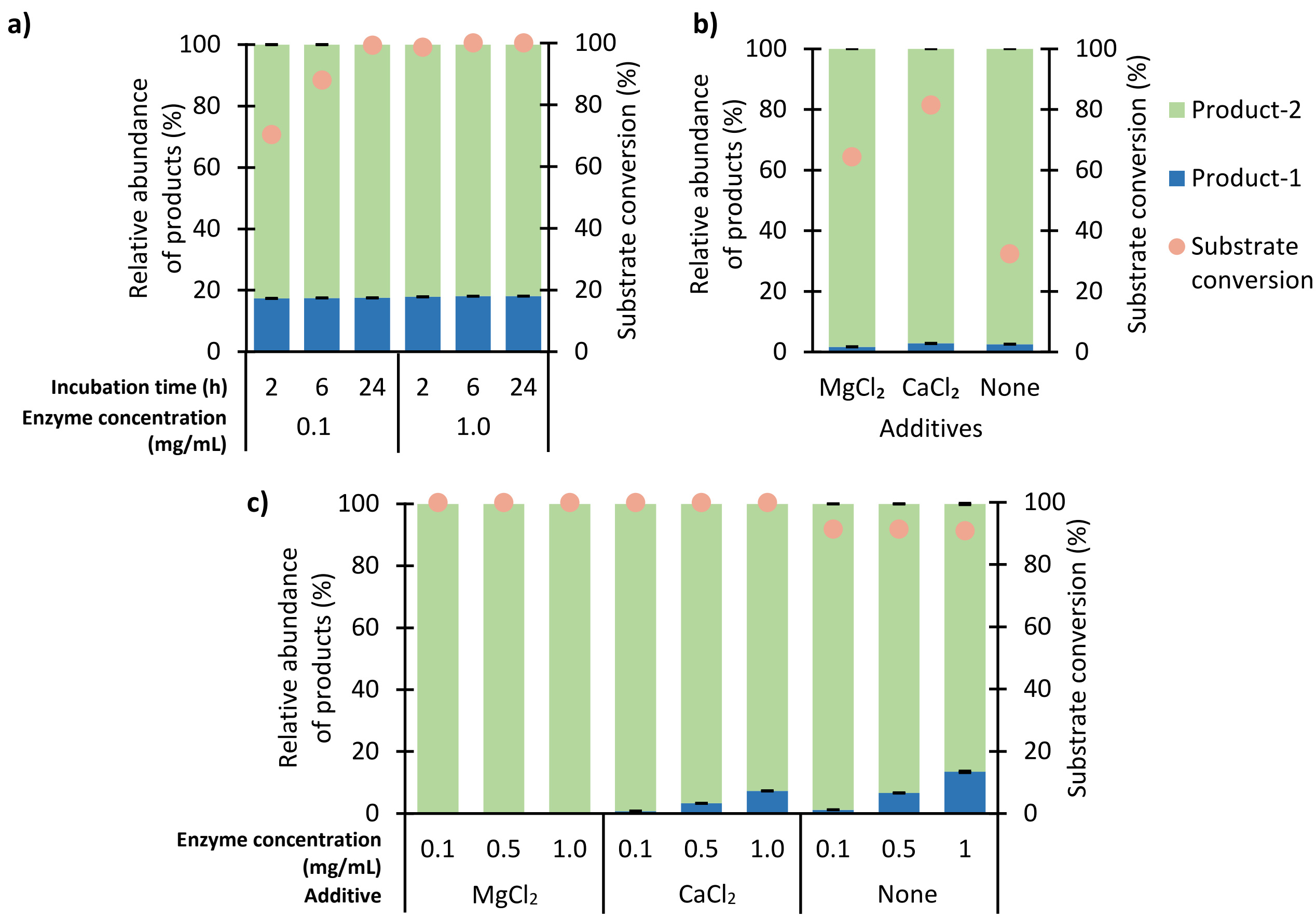

Fig. S15 Substrate conversion (%) and relative abundance of prenylated products (%) by RePT in the presence of DMAPP and **a) 1** when incubated for 2, 6, and 24 hours using 0.1 or 1.0 mg/mL RePT; **b) 8** when incubated for 24 hours using 1.0 mg/mL RePT with 5 mM $MgCl_2$, $CaCl_2$, or no additives; and **c)** L-tyrosine (**2**) when incubated for 24 hours using 0.1, 0.5, or 1.0 mg/mL RePT with 5 mM $MgCl_2$, $CaCl_2$, or no additives.

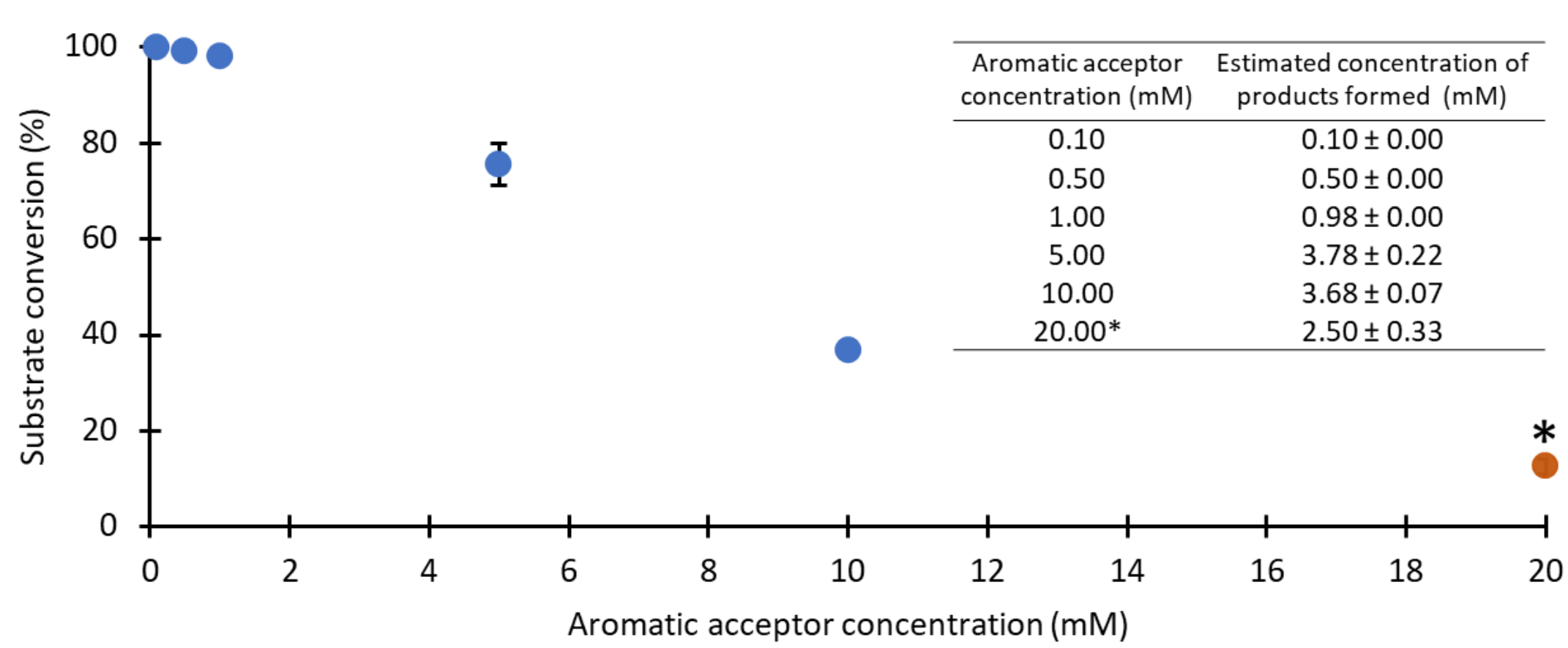

Aromatic acceptor concentration (mM)	Estimated concentration of products formed (mM)
0.10	0.10 ± 0.00
0.50	0.50 ± 0.00
1.00	0.98 ± 0.00
5.00	3.78 ± 0.22
10.00	3.68 ± 0.07
20.00*	2.50 ± 0.33

Fig. S16 Substrate conversion of **1** converted by RePT when incubated for 24 hours using different concentrations of **1** (0.1-20 mM). A concentration of 0.1 mg/mL RePT was used. The molar ratio of DMAPP to **1** was 2:1, except for the sample at 20 mM of **1** (the orange point with an asterisk), in which the ratio was 1:1. The estimated concentrations of the products formed are shown in the table.

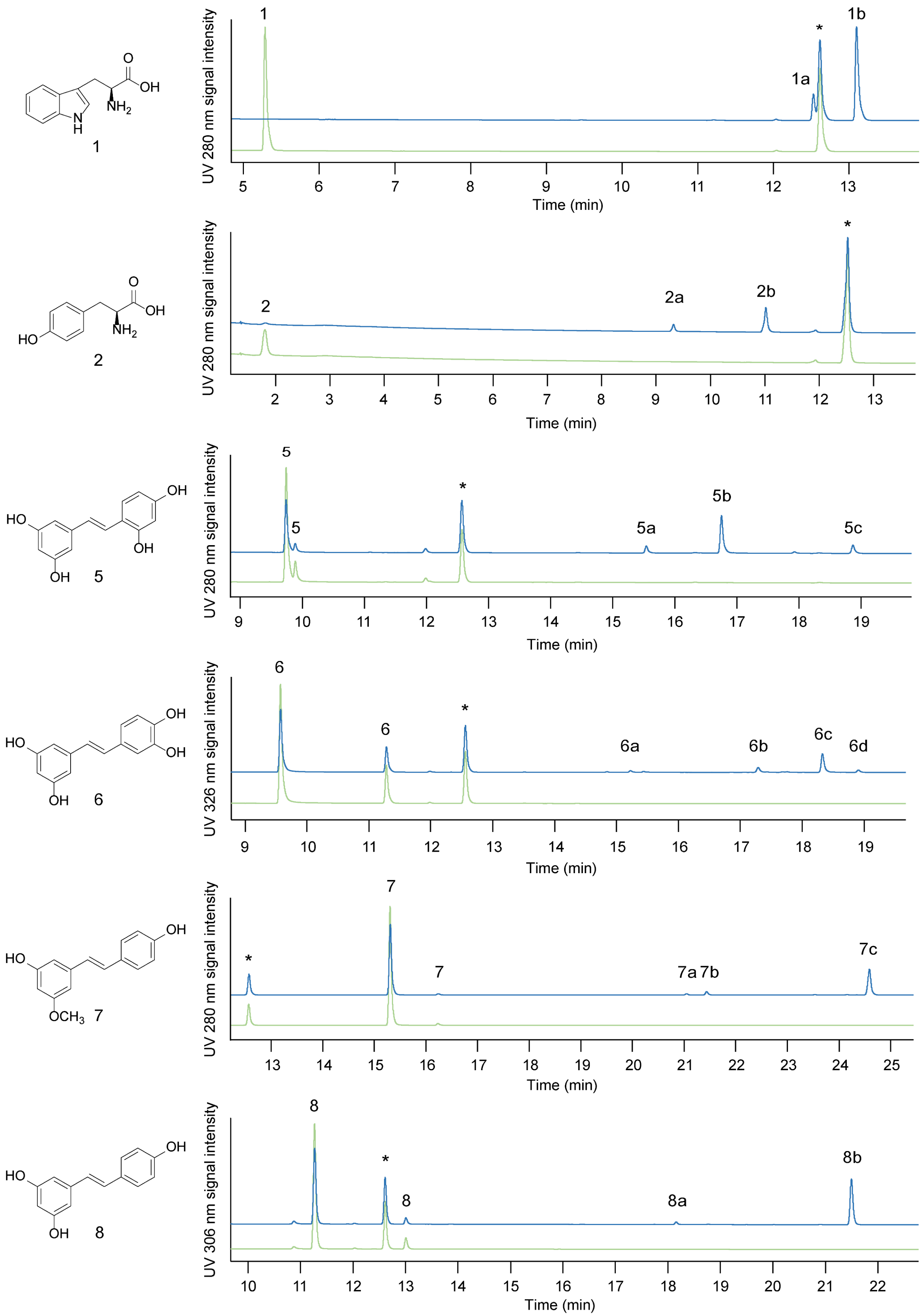

O
OH
NH2
N
H
1
UV 280 nm signal intensity
1a
*
1b
Time (min)
HO
2
2a
2b
5
5a
5b
5c
OH
6
6a
6b
6c
6d
UV 326 nm signal intensity
7
7a
7b
7c
OCH3
8
8a
8b
UV 306 nm signal intensity

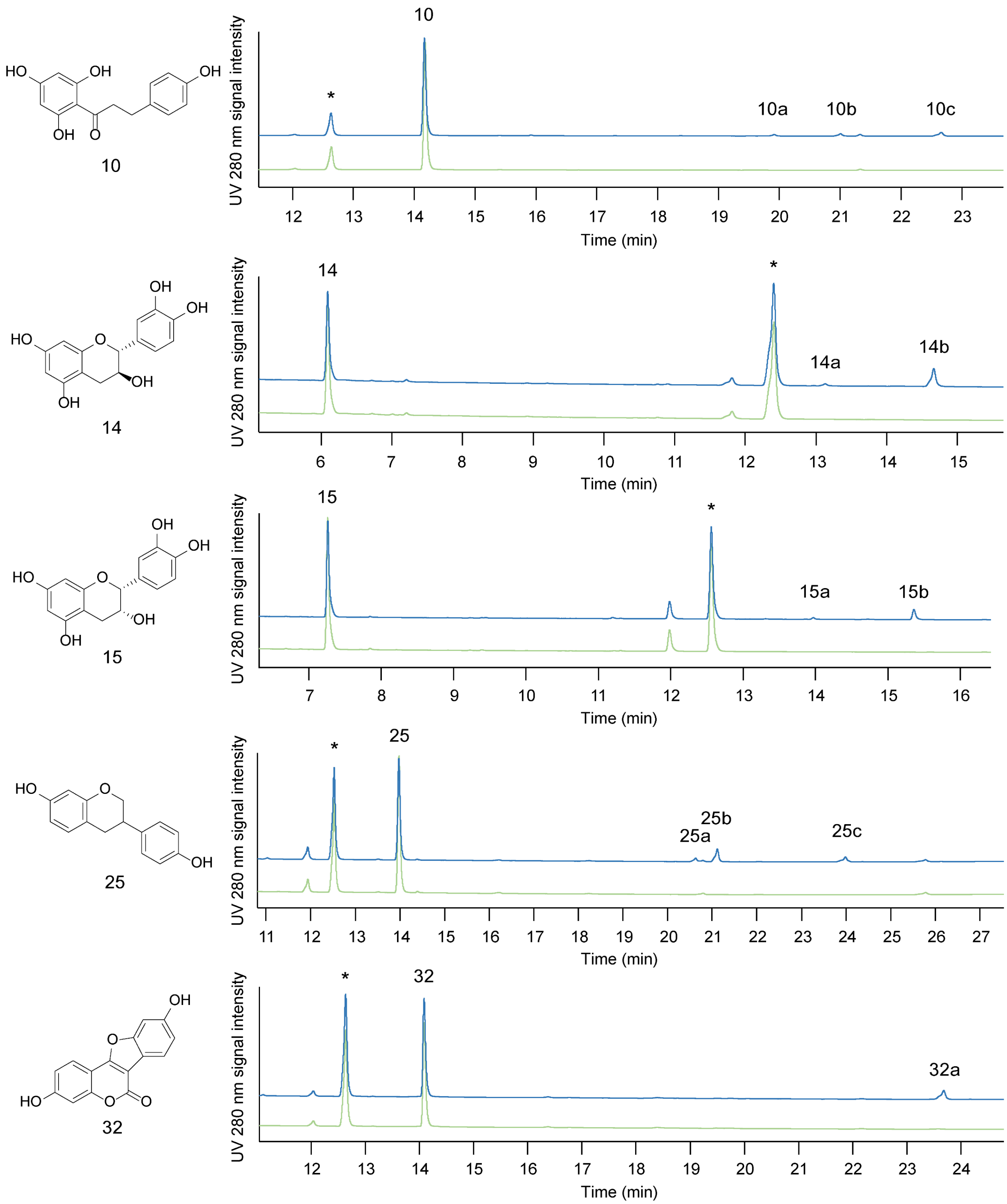

Fig. S17 UHPLC-UV chromatograms of enzymatic reactions of RePT towards well-accepted substrates in the presence of DMAPP (in blue) and mixtures in the absence of RePT and DMAPP (blanks, in green). An asterisk indicates the main peak of TMCA, the internal standard used in the analysis. Peak numbers refer to compound numbers, detailed data for each peak are reported in **Table S3**.

Table S3 Substrate conversion, UV and MS data, and the tentative identification of enzymatic products from RePT reactions in the presence of DMAPP and well-accepted substrates (higher than 10% conversion).

No.	Compound	Substrate conversion (%)	Rel. substrate or product abundance (%)[a,b]	Rt UV (min)	UV_{max}[c]	$[M-H]^-$	MS^2 NI (relative abundance)[d,e]	$[M+H]^+$	MS^2 PI (relative abundance)[d,e]	(Tentative) prenylation position
1	L-Tryptophan	100 ± 0.0	-	5.30	278	203	159 (100), 116 (40), 142 (15), 186 (11)	205	188 (100)	-
1a	L-Tryptophan-PR1		20	12.53	286	271	184 (100), 158 (30), 157 (14), 159 (11), 227 (6)	273	256 (100), **205 (7)**	n.i. / reverse *N*1 (NMR)
1b	L-Tryptophan-PR2		80	13.09	274	271	184 (100), 227 (64), 210 (50), 254 (13), 185 (5)	273	256 (100)	n.i. / normal *C*7 (NMR)
2	L-Tyrosine	91.7 ± 0.3	-	1.80	274	180	163 (100), 93 (14), 119 (13), 136 (11), 137 (5)	182	165 (100), 136 (27)	-
2a	L-Tyrosine-PR1		20	9.32	n.d.	248	204 (100), 231 (72), 187 (60), 161 (51), 174 (13), 175 (5)	250	233 (100), **194 (43)**, 204 (11)	n.i.
2b	L-Tyrosine-PR2		80	11.02	274	248	135 (100), 119 (32), 204 (27), 231 (27), **180 (22)**, 187 (20), 161 (20), **179 (7)**	250	165 (100), **182 (13)**, 204 (5), 233 (5)	n.i. / normal *O*4 (NMR)
5[f,g]	*trans*-Oxyresveratrol	54.5 ± 0.4	75	9.74	326, 302	243	225 (100), 199 (33), 201 (33), 181 (21), 185 (19), 159 (17), 175 (16), 228 (14), 215 (11), 157 (8), 173 (6)	245	227 (100), 226 (48), 135 (11), 199 (9), 161 (6), 203 (6), 209 (5)	-
	cis-Oxyresveratrol		25	9.88	318, 286	243	225 (100), 201 (32), 199 (30), 185 (22), 175 (20), 181 (19), 159 (18), 228 (14), 215 (11), 157 (7), 173 (5)	245	n.d.	-
5a	Oxyresveratrol-PR1		12	15.54	326, 286	311	293 (100), 187 (35), 267 (27), 123 (22), 175 (17), 249 (17), 135 (14), **243 (13)**, 296 (10), 269 (10), **242 (9)**, 225 (9), 283 (9), 185 (7), 201 (7), 253 (7), 241 (6), 227 (6)	313	n.d.	n.i.

No.	Compound	Substrate conversion (%)	Rel. substrate or product abundance (%)[a,b]	Rt UV (min)	UV_{max}[c]	$[M-H]^-$	MS^2 NI (relative abundance)[d,e]	$[M+H]^+$	MS^2 PI (relative abundance)[d,e]	(Tentative) prenylation position
5b	Oxyresveratrol-PR2		71	16.75	338, 302	311	293 (100), 187 (37), 267 (29), 123 (22), 249 (18), 175 (17), **243 (14)**, 135 (14), 296 (11), 269 (11), **242 (10)**, 225 (9), 283 (9), 185 (8), 201 (8), 253 (7), 241 (6), 227 (6), 294 (6)	313	239 (100), **257 (80)**, 294 (35), 295 (34), **245 (14)**, 227 (7), 256 (6), ***258 (6)***, 123 (5), 238 (5)	n.i.
5c	Oxyresveratrol-PR3		17	18.87	330	311	**242 (100)**, 241 (75), **243 (23)**, 293 (8)	313	n.d.	*O*-prenyl
6[f,g,h]	*trans*-Piceatannol	39.1 ± 1.6	79	9.71	326	243	255 (100), 201 (71), 199 (44), 175 (41), 200 (26), 243 (24), 215 (15), 159 (14), 173 (12), 185 (12), 228 (12), 214 (10), 181 (9), 198 (8), 197 (7), 157 (7), 226 (5)	245	n.d.	-
	cis-Piceatannol		21	11.35	262, 290	243	225 (100), 201 (64), 199 (44), 175 (41), 200 (26), 243 (21), 159 (13), 215 (13), 228 (12), 185 (12), 173 (11), 214 (10), 198 (9), 181 (9), 197 (7), 157 (7), 226 (6)	245	n.d.	-
6a	Piceatannol-PR1		1	15.23	334	311	**254 (100)**, 253 (33), 241 (29), **255 (21)**, 201 (18), 269 (17), 267 (15), ***256(11)***, 212 (10), **242 (9)**, 268 (8), 227 (8), **243 (7)**, 293 (7), 240 (6), 187 (6), 123 (6), 189 (6), 109 (6)	n.d.	n.d.	*C*-prenyl
6b	Piceatannol-PR2		14	17.29	330	311	n.d.	n.d.	n.d.	n.i.
6c	Piceatannol-PR3		83	18.33	286, 326	311	**242 (100)**, **243 (45)**, 241(22), 267 (12), 224 (10), 293(6)	313	n.d.	*O*-prenyl
6d	Piceatannol-PR4		3	18.91	314	311	**243 (100)**, **242 (47)**, 241 (10)	313	**245 (100)**, 257 (8)	*O*-prenyl

No.	Compound	Substrate conversion (%)	Rel. substrate or product abundance (%)[a,b]	Rt UV (min)	UV_{max}[c]	$[M-H]^-$	MS^2 NI (relative abundance)[d,e]	$[M+H]^+$	MS^2 PI (relative abundance)[d,e]	(Tentative) prenylation position
7[f,g]	*trans*-Pinostilbene	38.8 ± 0.5	94	15.31	306-318	241	225 (100), 226 (88)	243	149 (100), 225 (65), 133 (42), 211 (39), 121 (25), 119 (24), 147 (18), 197(16), 159 (13), 210 (12), 183 (11), 145 (11), 135 (11), 224 (9), 226 (9), 215 (8), 123 (8), 213 (8), 193 (7), 227 (7), 107 (7), 105 (7), 157 (6), 201 (6), 212 (6), 91 (6), 125 (6), 207 (5)	-
	cis-Pinostilbene		6	16.23	286	241	225 (100), 226 (86)	243	n.d.	-
7a	Pinostilbene-PR1		5	21.05	330	309	n.d.	311	n.d.	n.i.
7b	Pinostilbene-PR2		8	21.44	326	309	***254 (100)***, 293 (31), 294 (24), 255 (12), 309 (10), 310 (9), **253 (7)**, 251 (6)	311	n.d.	*C*-prenyl
7c	Pinostilbene-PR3		84	24.59	306-318	309	n.d.	311	**243 (100)**	*O*-prenyl
8[f,i]	*trans*-Resveratrol	36.7 ± 0.5	77	11.27	306	227	185 (100), 183 (42), 159 (34), 157 (28), 143 (14), 227 (14), 212 (5)	229	135 (100), 211 (54), 119 (21), 107 (19), 210 (16), 183 (12), 227 (7), 91 (6), 199 (6), 212 (6), 111 (5), 187 (5)	-
	cis-Resveratrol		23	13.01	290	227	185 (100), 183 (45), 159 (38), 157 (29), 143 (14), 227 (14)	229	n.d.	-
8a	Resveratrol-PR1		5	18.16	326	295	***240 (100)***, 251 (51), **239 (32)**, 253 (31), 225 (29), 295 (23), **226 (10)**, **227 (10)**	297	n.d.	*C*-prenyl

No.	Compound	Substrate conversion (%)	Rel. substrate or product abundance (%)[a,b]	Rt UV (min)	UV_{max}[c]	$[M-H]^-$	MS^2 NI (relative abundance)[d,e]	$[M+H]^+$	MS^2 PI (relative abundance)[d,e]	(Tentative) prenylation position
8b	Resveratrol-PR2		95	21.50	306	n.d.	n.d.	297	**229 (100)**, **241 (69)**, 191 (17), 223 (17)	*O*-prenyl/ *O4′*-prenyl (NMR)
10	Phloretin	21.9 ± 0.1	-	14.18	286	273	167 (100)	275	107 (100), 149 (28), 169 (28), 127 (11)	-
10a	Phloretin-PR1		17	19.92	n.d.	341	167 (100), 123 (9)	343	217 (100), 175 (78), 325 (7), 127 (6)	n.i.
10b	Phloretin-PR2		28	21.01	290	341	235 (100), 193 (5), 191 (5)	343	**287 (100)**	*C*-prenyl
10c	Phloretin-PR3		56	22.66	286	341	**272 (100)**, 166 (5),**273 (5)**	343	n.d.	*O*-prenyl
14	(+)-Catechin	23.3 ± 0.3	-	6.09	278	289	245 (100), 205 (36), 179 (13), 203 (9), 231 (6), 247 (6)	291	123 (100), 139 (98), 165 (50), 273 (27), 151 (23), 147 (9)	-
14a	(+)-Catechin-PR1		9	13.13	n.d.	357	**288 (100)**, **289 (48),** 313 (21), 165 (20), 179 (9), 137 (8)	359	**291 (100)**, 191 (22), 273 (19), 303 (16), 139 (15), 341 (12), 207 (5), 123 (5)	*O*-prenyl
14b	(+)-Catechin-PR2		90	14.67	n.d.	357	**288 (100)**, 150 (56), **289 (42)**, 313 (26), 166 (22), 137 (14), 179 (10), 165 (9), 226 (8), 273 (6), 270 (5)	359	**291 (100)**, 191 (21), 123 (18), 273 (17), 139 (13), 341 (6), 303 (5)	*O*-prenyl
15	(-)-Epicatechin	14.6 ± 0.7	-	7.26	278	289	245 (100), 205 (35), 179 (13), 203 (10), 231 (5), 247 (5)	291	123 (100), 139 (100), 165 (61), 273 (32), 151 (25), 147 (11)	-
15a	(-)-Epicatechin-PR1		13	13.97	n.d.	357	n.d.	359	n.d.	n.i.
15b	(-)-Epicatechin-PR2		87	15.36	278	357	150 (100), **288 (85)**, **289 (48)**, 166 (25), 137 (17), 313 (15), 165 (11), 226 (5), 179 (5)	359	**291 (100)**, 273 (16), 123 (15), 191 (12), 139 (9)	*O*-prenyl
25	(±)-Equol	25.1 ± 0.5	-	13.98	282	241	n.d.	243	123 (100), 107 (48), 134 (34), 133 (14), 135 (11), 137 (7), 132 (7), 108 (5)	-

No.	Compound	Substrate conversion (%)	Rel. substrate or product abundance (%)[a,b]	Rt UV (min)	UV_{max}[c]	[M−H]⁻	MS² NI (relative abundance)[d,e]	[M+H]⁺	MS² PI (relative abundance)[d,e]	(Tentative) prenylation position
25a	(±)-Equol-PR1		16	20.62	n.d.	n.d.	n.d.	311	n.d.	n.i.
25b	(±)-Equol-PR2		55	21.11	n.d.	n.d.	n.d.	311	n.d.	n.i.
25c	(±)-Equol-PR3		29	23.99	n.d.	n.d.	n.d.	311	**243 (100)**, 191 (59), 255 (51), 123 (36), 175 (30), 205 (24), 241 (23), 107 (13), 189 (12), 133 (7), 293 (6), 149 (6), 135 (5)	*O*-prenyl
32	Coumestrol	10.9 ± 0.4	-	14.08	<u>342</u>, 306	267	267 (100), 239 (21), 268 (21), 266 (19)	269	241 (100), 197 (33), 225 (26), 242 (10), 269 (6), 268 (5)	-
32a	Coumestrol-PR1		100	23.68	346	335	<u>**266 (100)**</u>	337	**269 (100)**	*O*-prenyl

[a] Relative abundance of substrate was calculated using the substrate blanks incubated at 37 °C for 24 hours. [b] Relative abundance of prenylated products, only prenylated products with relative product abundance ≥ 5% are shown, except for prenylated products of **6** for which the relative abundance of each product greatly changed when using different UV wavelengths for calculation. [c] The underlined UV-Vis wavelength corresponds to the main peak. [d] Only fragments with relative abundance ≥ 5% are reported. [e] Fragments with a neutral loss of 55 (in case of prenylated stilbenes), 56, 57, 68, or 69 u are displayed in ***italic blue bold text***, **blue bold text**, <u>**underlined blue bold text**</u>, **black bold text**, and <u>**underlined black bold text**</u>, respectively. [f] Stilbenes may occur as *cis*- and *trans*- isomers, showing two substrate peaks. Substrate conversion was calculated using the combination of a peak area of both isomers and shown as one value. [g] A correction factor for UV signal intensity between *cis*- and *trans*- isomers was unknown for this substrate, therefore semi-quantification was performed without correction. [h] UV signal intensity at 326 nm was used to semi-quantify **6 and 6a-d** instead of UV 280 nm to avoid an error due to a co-eluting background peak. [i] UV signal intensity at 306 nm was used to semi-quantify *cis*- and *trans*- resveratrol. The intensity was corrected for a difference in the molecular extinction coefficients between the two isomers using the ratio of 3.426: 1 according to a known correction factor from Trela & Waterhouse (1996). Abbreviations: n.d. = not detected; n.i. = annotation guideline was not applicable to identify prenylation position or fragmentation pattern not available.

Table S4 Substrate recovery (%) of prenyl acceptor substrates with > 10 % conversion by RePT after an incubation at 37 °C for 24 hours.

No.	Compound	Recovery (%)
1	L-Tryptophan	100.0 ± 0.0
2	L-Tyrosine	99.8 ± 0.1
5	Oxyresveratrol	90.3 ± 0.2[a]
6	Piceatannol	88.1 ± 1.8[a]
7	Pinostilbene	99.9 ± 0.5[a]
8	Resveratrol	102.2 ± 3.0
10	Phloretin	103.5 ± 0.1
14	(+)-Catechin	89.3 ± 0.2
15	(-)-Epicatechin	91.3 ± 0.5
25	(±)-Equol	100 ± 0.1
32	Coumestrol	76.7 ± 0.3

[a] The recovery was calculated by using the sum of the two isomers of the substrates without taking into account the difference in UV response between isomers.

Table S5 NMR data of **1a** and **1b** in 70 % (*v/v*) methanol-d4 in deuterium oxide, **2b** and **8b** in DMSO-d6 (δ in ppm, *J* in Hz).

Posi-tion	**1a**		**1b**		**2b**		**8b**	
	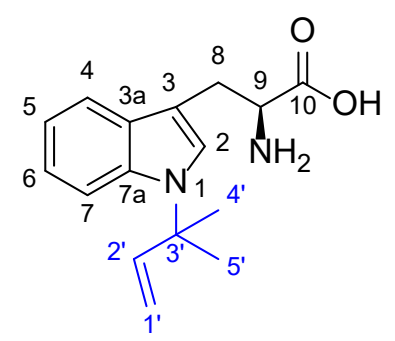		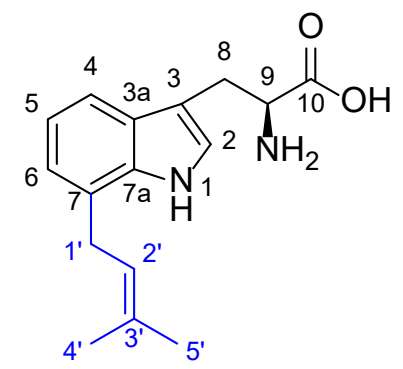		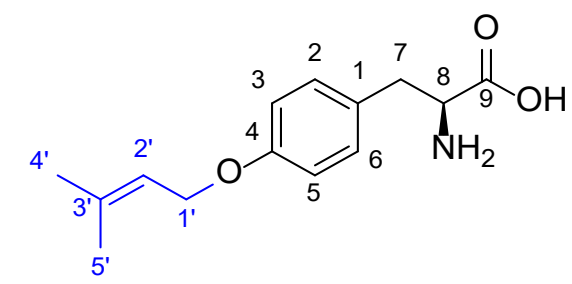		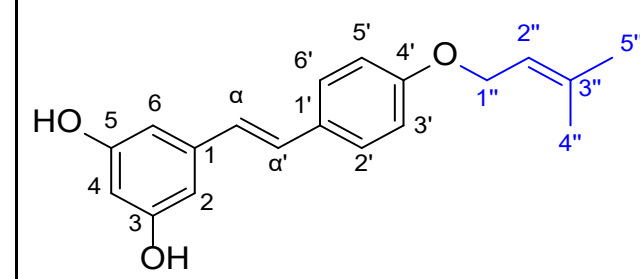	
	δ_H, mult., *J*[a]	δ_C	δ_H, mult., *J*	δ_C	δ_H, mult., *J*	δ_C	δ_H, mult., *J*	δ_C
1	-	-	-	-	-	129.6	-	139.6
2	7.43, s	126.5	7.28, s	125.5	7.15, d, 7.9	130.8	6.40, d, 2.0	104.9
3	-	108.0	-	109.7	6.84, d, 7.6	114.9	-	159.0
3a	-	130.4	-	128.4	-	-	-	-
4	7.70, dd, 2.0, 6.9	119.9	7.57, d, 7.8	117.4	-	157.6	6.13, t, 2.1	102.4
5	7.08, m, 6.8	120.3	7.05, t, 7.3	120.8	6.84, d, 7.6	114.9	-	159.0
6	7.10, m, 6.8	122.1	6.96, d, 7.1	122.1	7.15, d, 7.9	130.8	6.40, d, 2.0	104.9
7	7.51, d, 7.9	115.3	-	126.2	2.78, dd, 8.4, 14.3	36.5	-	-
	-	-	-	-	3.06, dd, 4.4, 14.3		-	-
7a	-	137.1	-	136.9	-	-	-	-
8	3.51, dd, 4.9, 15.3	28.1	3.23, dd, 8.7, 15.0	28.2	3.37, n.d.[c]	56.0[d]	-	-
	3.21, dd, 4.3, 8.5		3.52, dd, 4.3, 15.3		-	-	-	-
9	3.95, m, n.d.[b]	56.7	3.96, dd, 4.1, 8.8	56.7	-	-	-	-
10	-	174.8	-	-	-	-	-	-
1′	5.21, d, 10.7	114.3	3.57, d, 7.4	30.5	4.49, d, 6.3	64.6	-	158.6
	5.16, d, 17.6		-	-	-	-	-	-
2′	6.17, dd, 10.6, 17.7	145.4	5.43, t, 7.4	122.9	5.42, t, 6.8	120.6	7.50, d, 8.7	128.2
3′	-	60.4	-	134.7	-	137.3	6.92, d, 8.4	115.3
4′	1.76, s	28.2	1.76, s	18.1	1.70, s	18.5	-	130.0
5′	1.76, s	n.f.	1.76, s	26.1	1.74, s	25.9	6.92, d, 8.4	115.3
6′	-	-	-	-	-	-	7.50, d, 8.7	128.2
α	-	-	-	-	-	-	6.89, d, 16.4	127.1
α′	-	-	-	-	-	-	6.97, d, 16.4	128.0
1″	-	-	-	-	-	-	4.54, d, 6.7	64.8
2″	-	-	-	-	-	-	5.44, t, 6.6	120.4
3″	-	-	-	-	-	-	-	137.5
4″	-	-	-	-	-	-	1.72, s	18.5
5″	-	-	-	-	-	-	1.75, s	25.9
OH	-	-	-	-	-	-	9.20, s	-

[a] s: singlet, d: doublet, dd: doublet of doublets, t: triplet, m: multiplet; [b] overlapped with the peaks from **1b**; [c] overlapped with a residual solvent peak (H_2O); [d] Peak not observed in the ^{13}C spectrum, but HMBC and/or HSQC correlations confirmed the chemical shift. Abbreviations: n.d. = no data due to peak overlapping, n.f. = not found

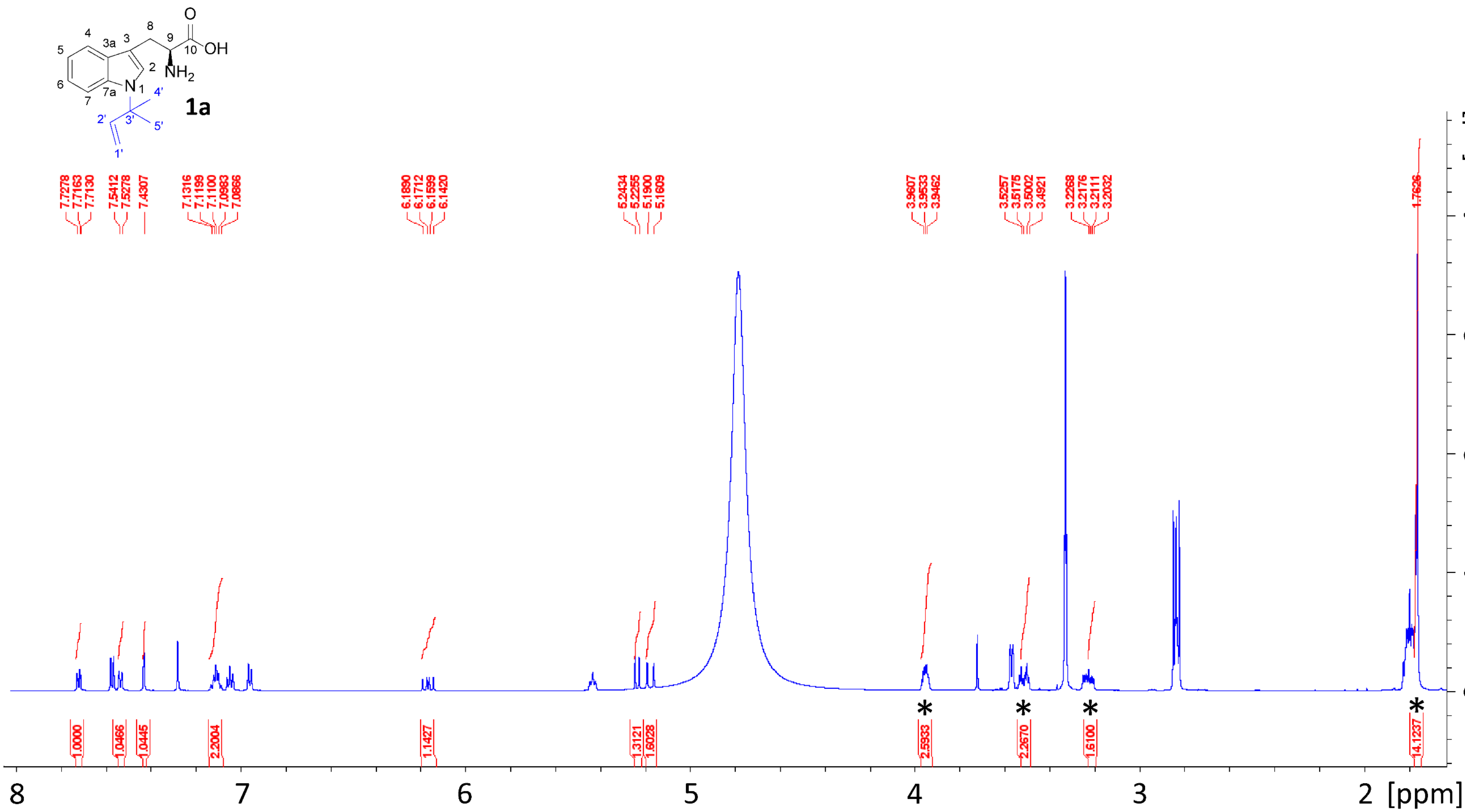

Fig. S18 ^{1}H-NMR spectrum (600 MHz) of **1a** recorded in a mixture of 70 % (*v/v*) methanol-d4 and 30 % (*v/v*) deuterium oxide. Peaks with an asterisk were integrated including signals from both **1a** and **1b**, with a peak area ratio of **1a**:**1b** = 1:1.4.

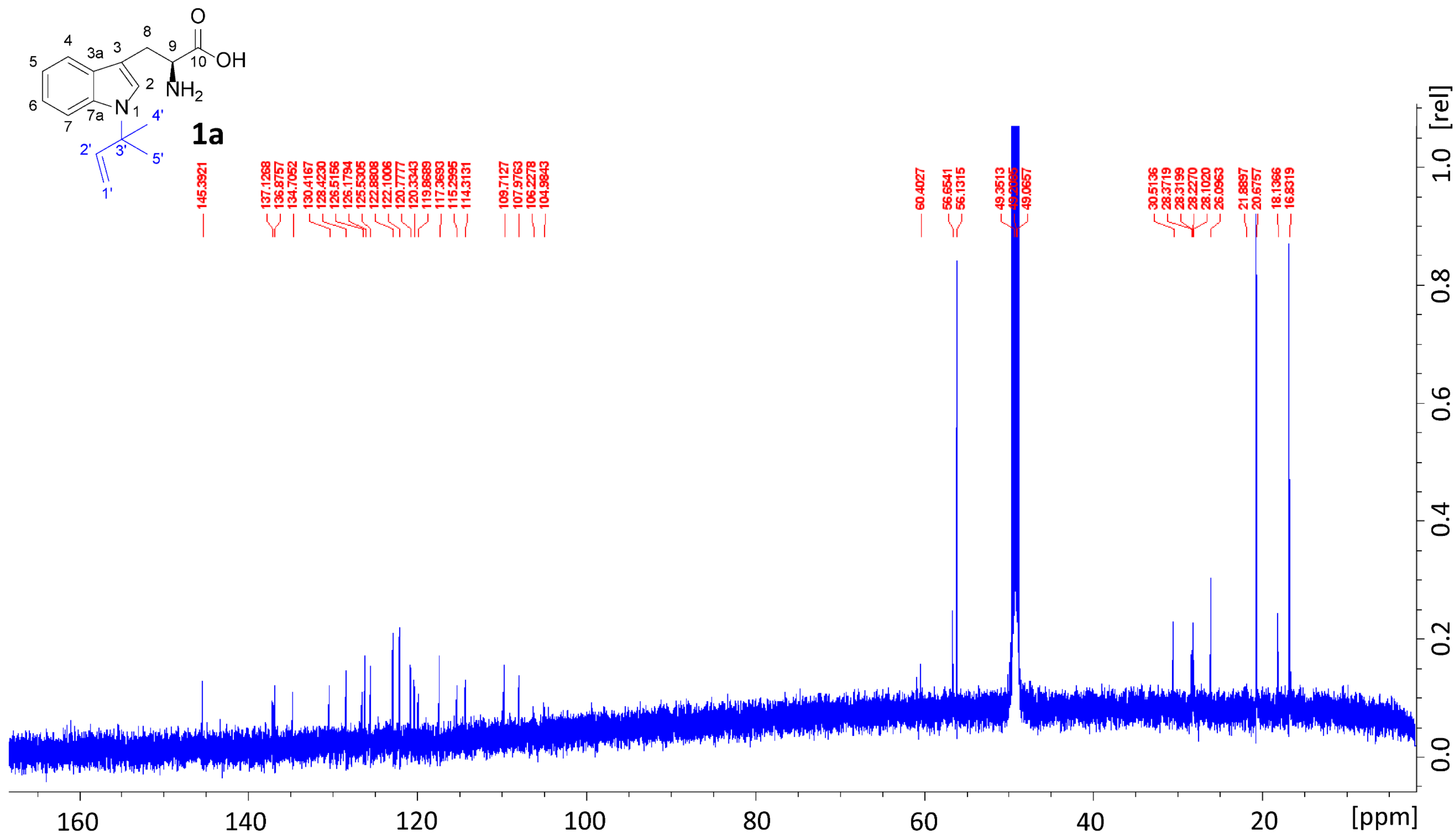

Fig. S19 ^{13}C spectrum (150 MHz) of **1a** recorded in a mixture of 70 % (*v/v*) methanol-d4 and 30 % (*v/v*) deuterium oxide.

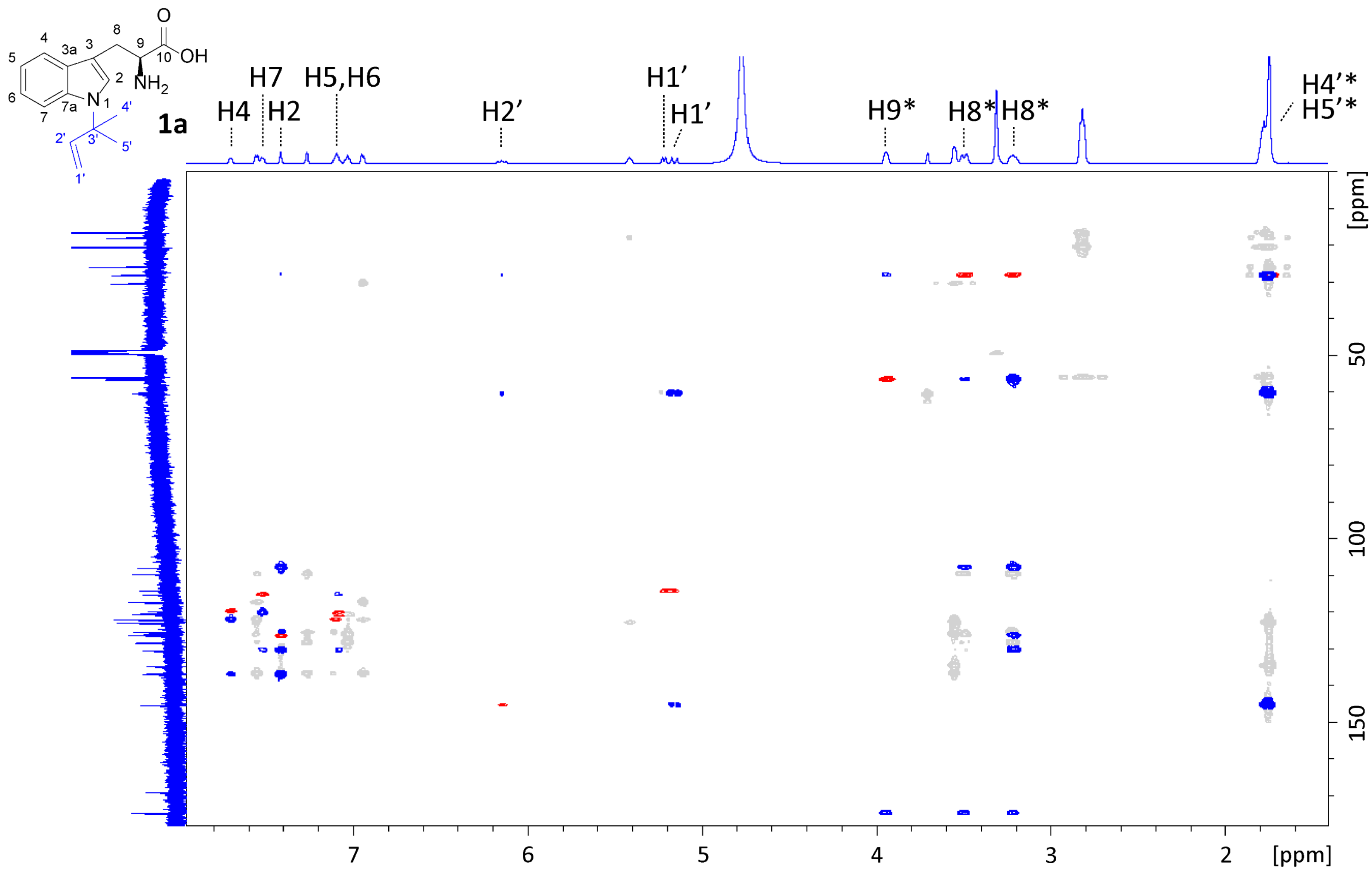

Fig. S20 ^{1}H(600 MHz)-^{13}C (150 MHz) spectra and HSQC (red) and HMBC (blue) correlations of **1a** recorded in a mixture of 70 % (*v/v*) methanol-d4 and 30 % (*v/v*) deuterium oxide. Peaks with an asterisk were integrated including signals from both **1a** and **1b**. Peaks of the impurities (correlations shown in grey) mostly came from the presence of **1b**, while a few peaks were from unidentified impurities. Due to the lack of peak splitting in the extrapolated ^{1}H spectrum, a separate ^{1}H spectrum (**Fig. S18**) was used to acquire accurate peak shapes for data interpretation.

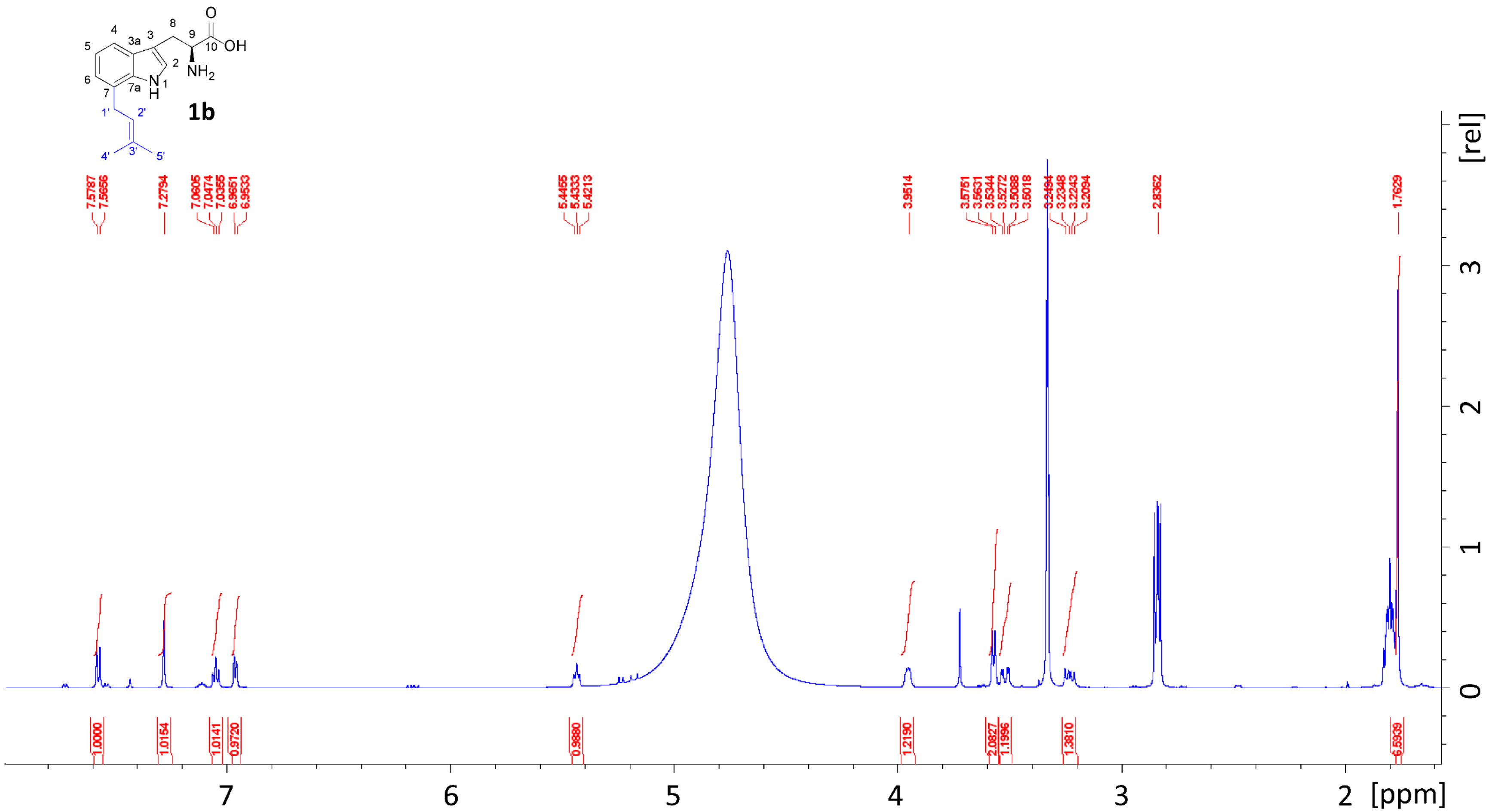

Fig. S21 ^{1}H-NMR spectrum (600 MHz) of **1b** recorded in a mixture of 70 % (*v/v*) methanol-d4 and 30 % (*v/v*) deuterium oxide.

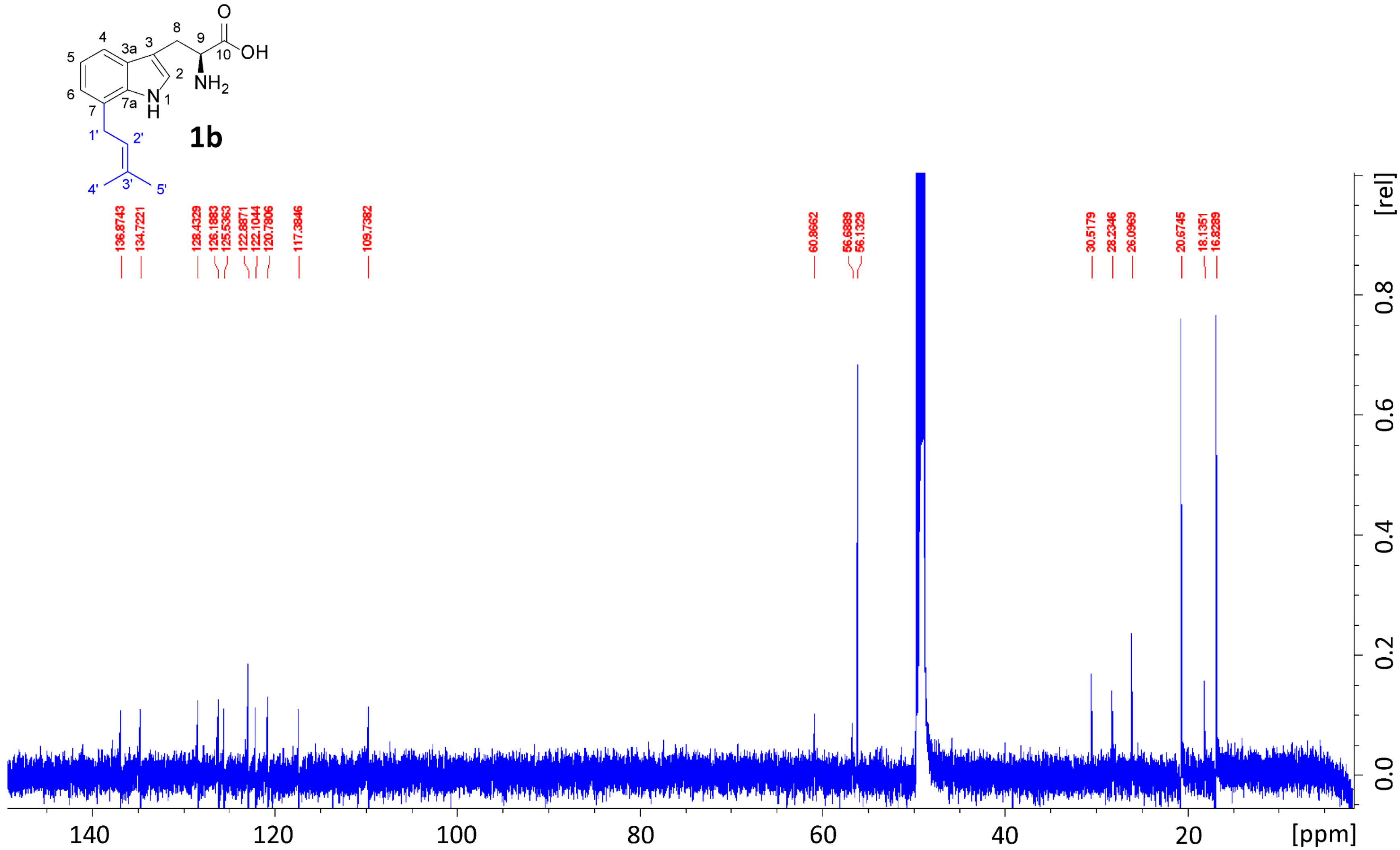

Fig. S22 ^{13}C spectrum (150 MHz) of **1b** recorded in a mixture of 70 % (*v/v*) methanol-d4 and 30 % (*v/v*) deuterium oxide.

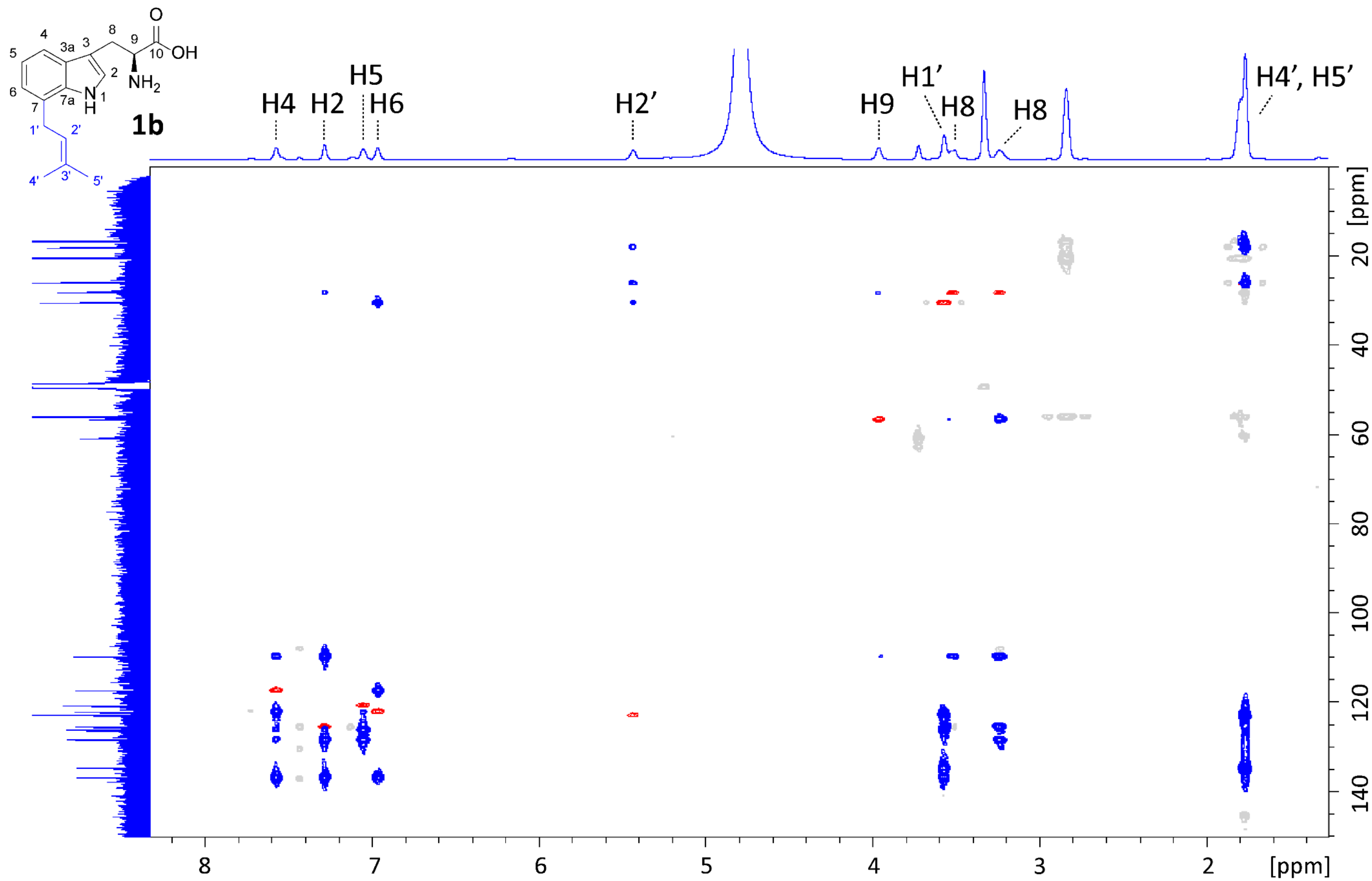

Fig. S23 ^{1}H(600 MHz)-^{13}C (150 MHz) spectra and HSQC (red) and HMBC (blue) correlations of **1b** recorded in a mixture of 70 % (*v/v*) methanol-d4 and 30 % (*v/v*) deuterium oxide. Peaks of the impurities (correlations shown in grey) mostly came from the trace presence of **1a**, while a few peaks were from unidentified impurities. Due to the lack of peak splitting in the extrapolated ^{1}H spectrum, a separate ^{1}H spectrum (**Fig. S21**) was used to acquire accurate peak shapes for data interpretation.

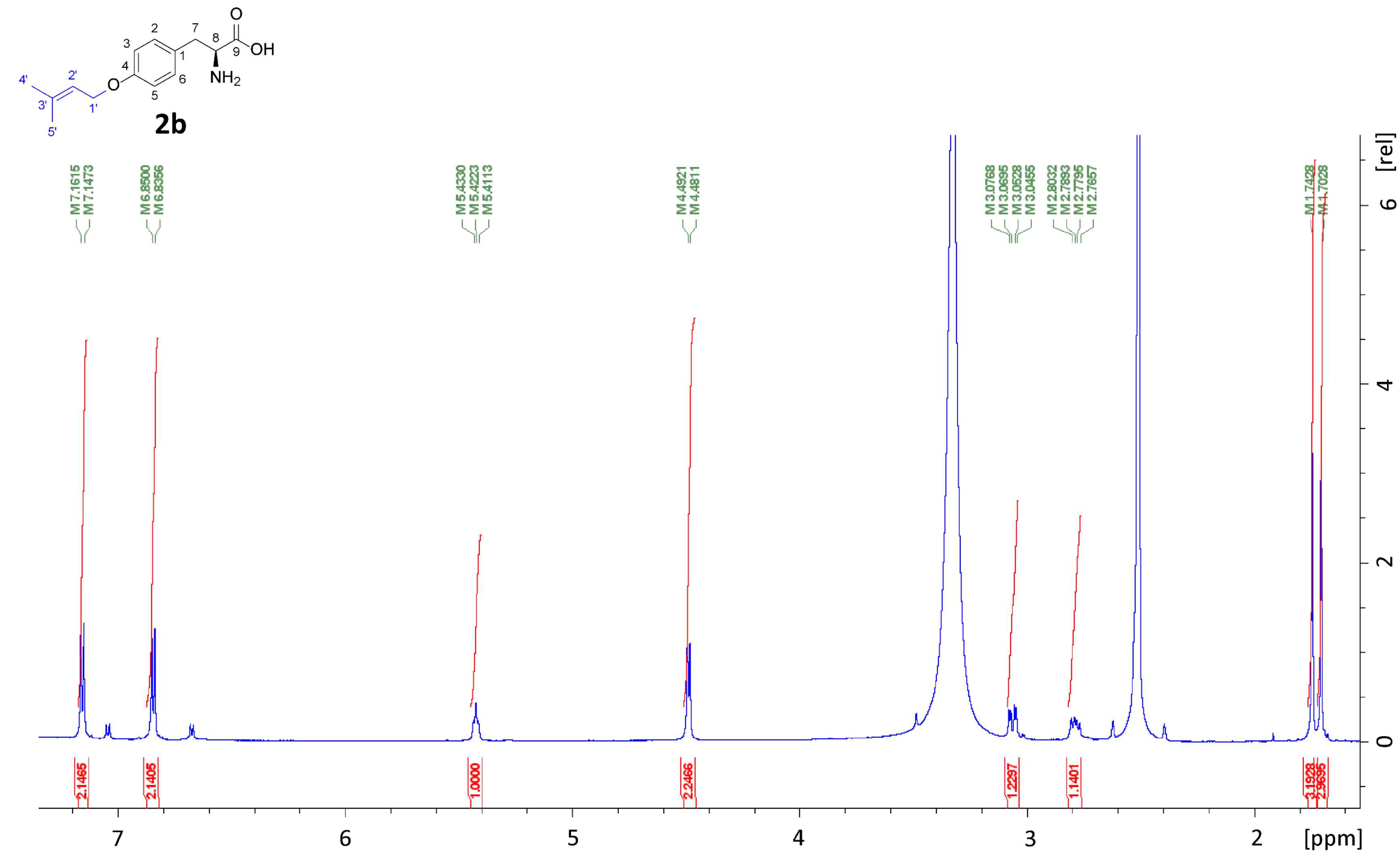

Fig. S24 ^{1}H-NMR spectrum (600 MHz) of **2b** recorded in DMSO-d6.

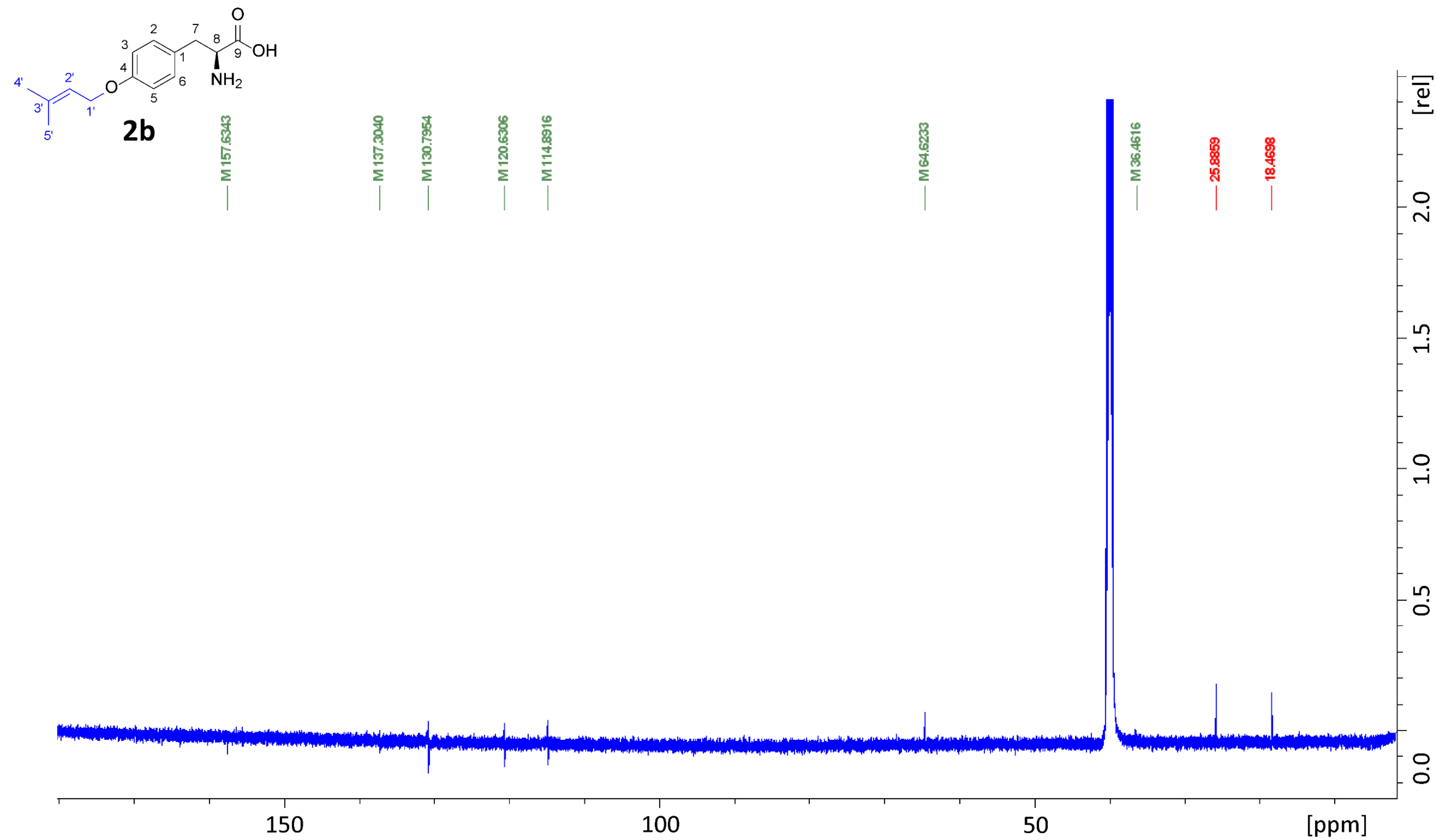

Fig. S25 ^{13}C spectrum (150 MHz) of **2b** recorded in DMSO-d6.

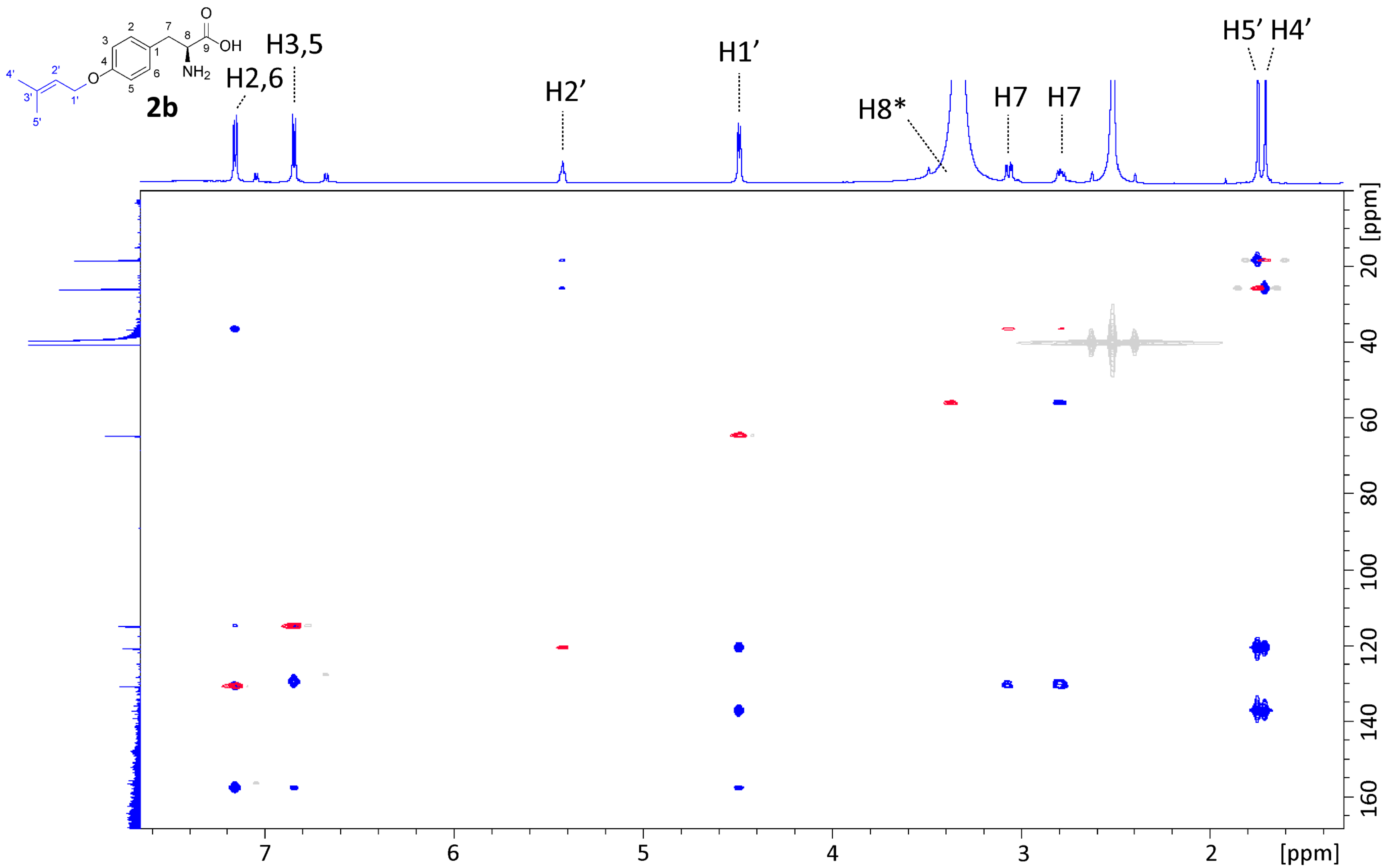

Fig. S26 ^{1}H(600 MHz)-^{13}C (150 MHz) spectra and HSQC (red) and HMBC (blue) correlations of **2b** recorded in DMSO-d6.

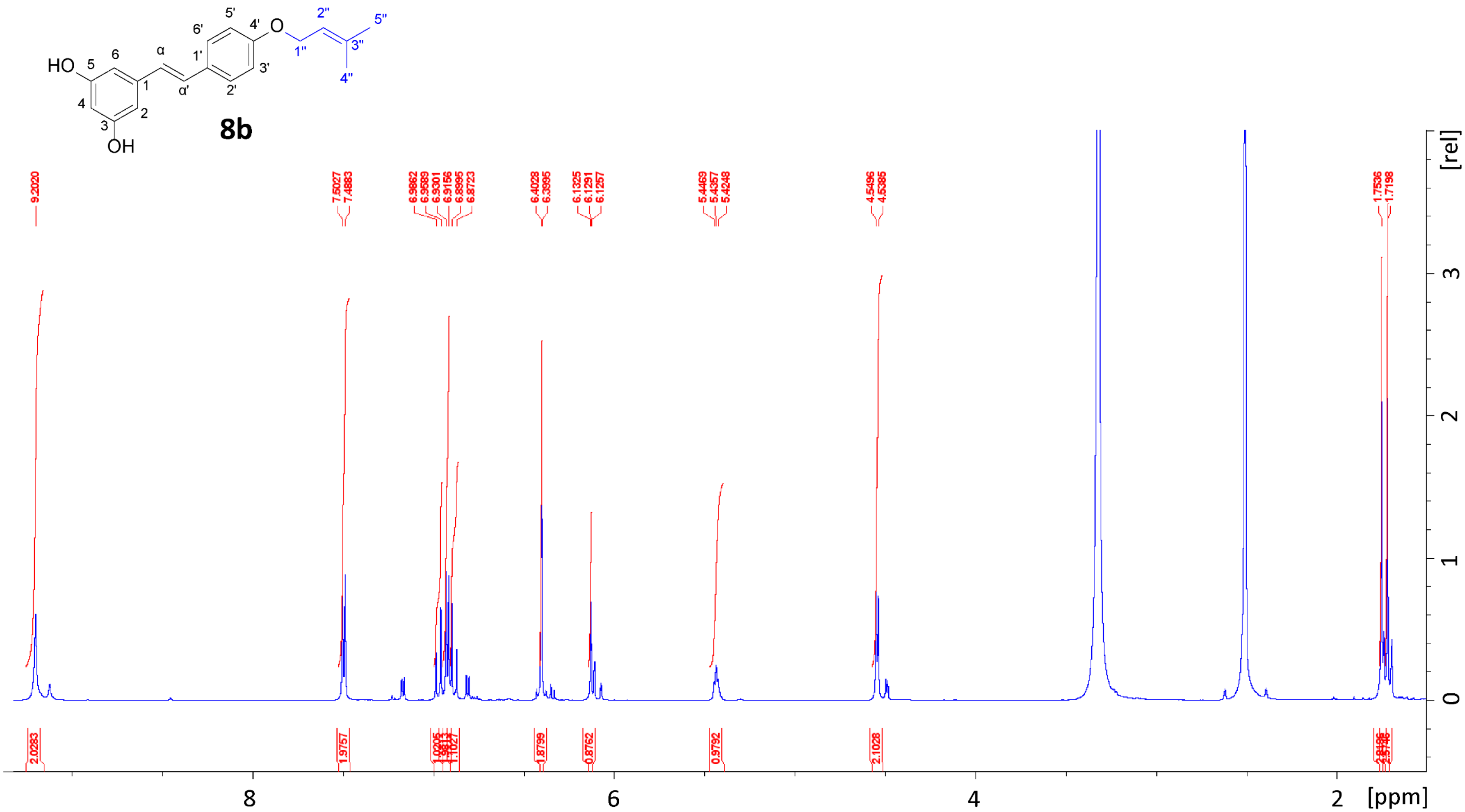

Fig. S27 ^{1}H-NMR spectrum (600 MHz) of **8b** recorded in DMSO-d6.

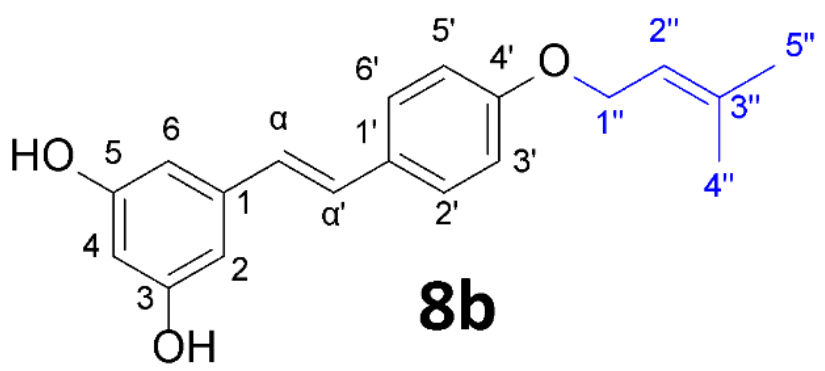

8b

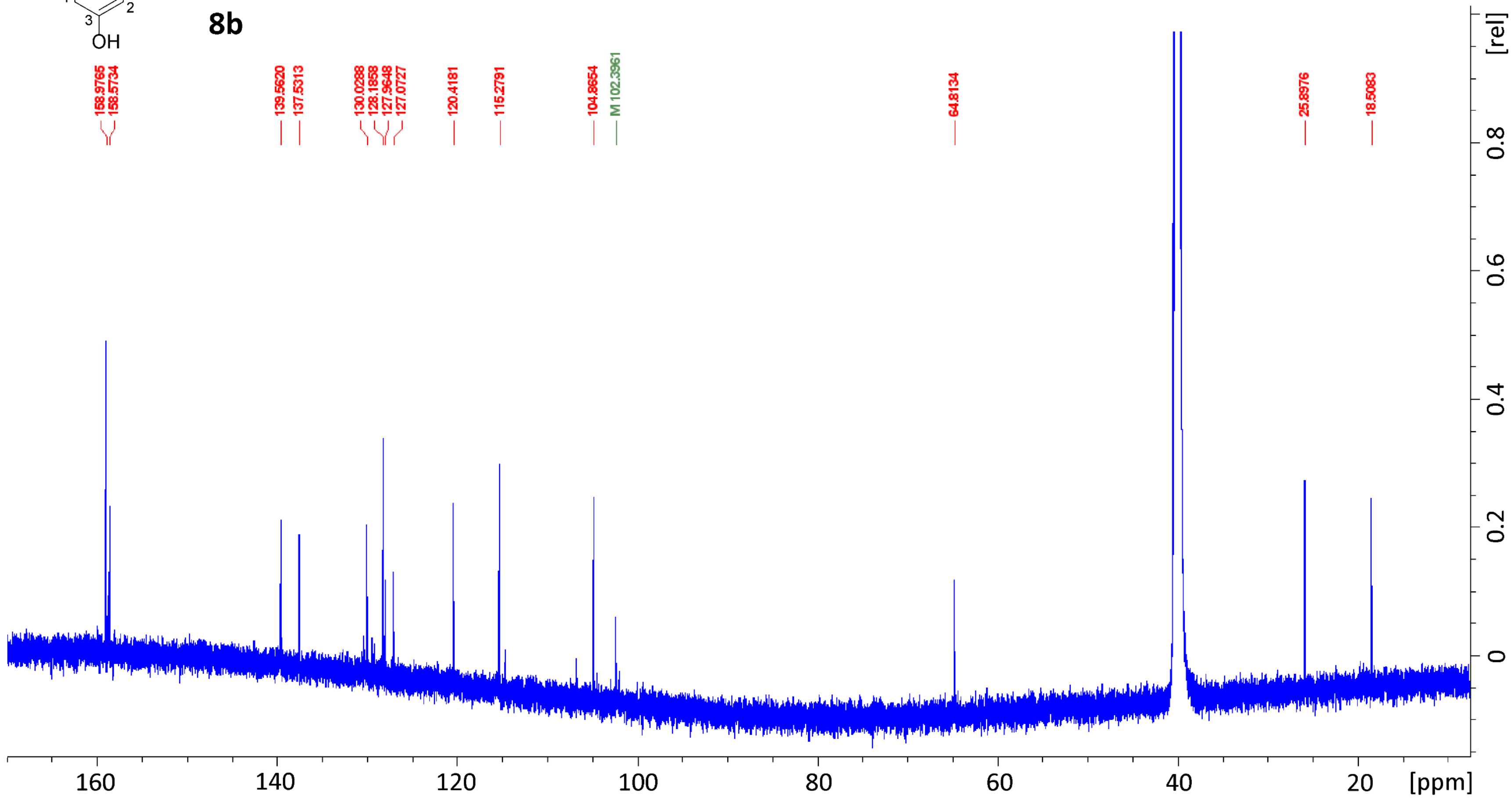

Fig. S28 ^{13}C spectrum (150 MHz) of **8b** recorded in DMSO-d6.

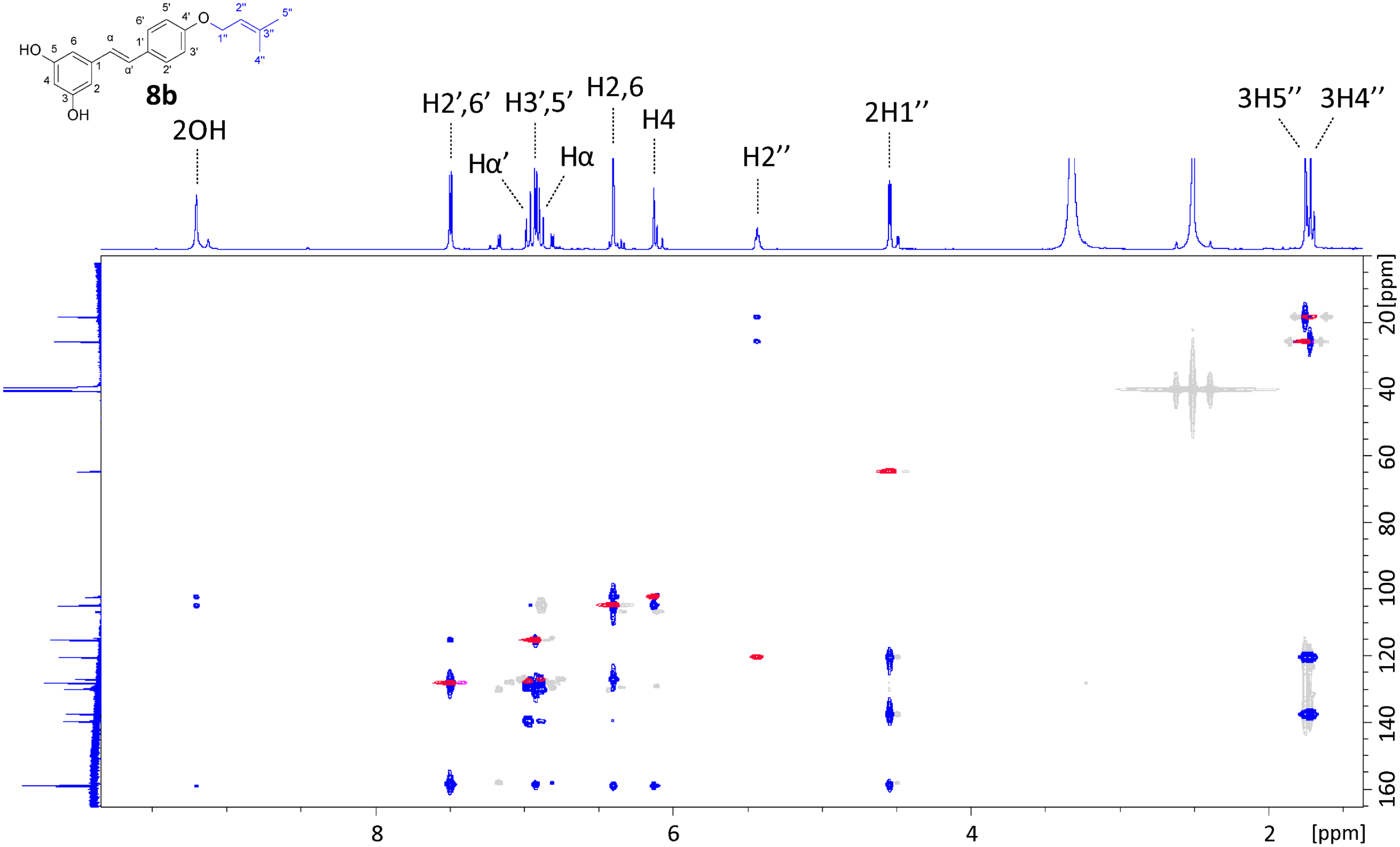

Fig. S29 ^{1}H(600 MHz)-^{13}C (150 MHz) spectra and HSQC (red) and HMBC (blue) correlations of **8b** recorded in DMSO-d6.

Table S6 ESI-FT-MS data of the purified prenylated products produced using RePT

Compound	Chemical formula	$[M+H]^+$		Deviation (ppm)
		Calculated	Observed	
*N*1-prenyl-L-tryptophan (**1a**)	$C_{16}H_{20}O_2N_2$	273.15975	273.16019	1.59
*C*7-prenyl-L-tryptophan (**1b**)	$C_{16}H_{20}O_2N_2$	273.15975	273.15991	0.57
*O*4-prenyl-L-tyrosine (**2b**)	$C_{14}H_{19}O_3N$	250.14377	250.14401	0.96
*O*4′-prenyl-resveratrol (**8b**)	$C_{19}H_{20}O_3$	297.14852	297.14871	0.64

Synthesis of dimethylallyl pyrophosphate tri-ammonium salt (DMAPP)

The synthesis of DMAPP was performed based on previously published protocols (Woodside et al. 1988; Chekan et al. 2020; Eggbauer et al. 2022) with several adaptations. Tris(tetrabutylammonium) hydrogen pyrophosphate 3.09 g (3.32 mmol) was dissolved in 1.5 mL acetonitrile (ULC/MS-grade). Prenyl bromide 202 µl (1.66 mmol) was added to the solution. The mixture was flushed with nitrogen and left to stir at room temperature for 5 h. The yellowish oil was then mixed with 1 mL of ion exchange buffer (2 g/L of NH_4HCO_3 in 2 % (*v/v*) isopropyl alcohol in water) and loaded onto a pre-treated ion-exchange column. The ion-exchange column was prepared by washing 40 mL dry volume of DOWEX AG 50W-X8 resin (H+ form, 100–200 mesh) (Bio-Rad Laboratories, Hercules, CA, USA) packed in a glass chromatography column with 170 mL of concentrated aq. ammonia. The column was further rinsed with 560 mL of water until the eluate reached pH 7. The column was equilibrated with 130 mL of ion-exchange buffer containing the reaction mixture. After the mixture was loaded, the reaction flask was rinsed with an additional 2 portions of 1 mL ion-exchange buffer, which were also loaded onto the column. The elution was performed with 80 mL of ion-exchange buffer (2 column volumes). Four fractions were collected and the presence of DMAPP was confirmed with thin-layer chromatography (TLC). Separation of DMAPP with TLC (R_f = 0.52) in each fraction was performed with 1-propanol:NH_4OH:water (8:5:2) on a silica gel plate. The reaction products were visualized by staining with $KMnO_4$ stain (200 mL water containing 1.5 g $KMnO_4$, 10 g K_2CO_3, and 1.25 mL of 10 % NaOH). The fractions containing DMAPP were pooled and lyophilized, yielding 718 mg of white powder.

The purity of the synthesized DMAPP was assessed by testing substrate conversion with RePT and L-tryptophan at the same reaction condition as described in the substrate specificity test. Substrate conversion was reduced from 100 % to 59 % when using the synthesized DMAPP obtained after ion exchange chromatography compared to the DMAPP standard. Additionally, the synthesized DMAPP was analyzed by HILIC-UHPLC-ESI-IT-MS^n. A UHPLC-MS system identical to the one described in the substrate specificity test was equipped with an Acquity UPLC BEH HILIC column (2.1 x 150 mm, 1.7 µm) (Waters, Milford, MA, USA). The column temperature was set at 25 °C. The flow rate used was 400 µL/min with the following eluents: (A) 25 mM NH_4HCO_3 in water and (B) 25 mM NH_4HCO_3 in 10 % (*v/v*) water in acetonitrile. The elution gradient was 1 min isocratic 100 %B; 22 min linear gradient 100-90 %B; 5 min linear gradient 90-80 %B; 1 min linear gradient 80-100 %B; 12 min isocratic 100 %B. Mass spectrometric analysis was performed with the same MS system and settings as described in the substrate specificity test, with the exception of using a mass acquisition range of *m/z* 140-1000. Additionally, dynamic mass exclusion settings were adjusted. Most intense ion was fragmented 4 times with a repeat duration of 15 s and then excluded for fragmentation for 15 s. The purity of DMAPP was determined, based on MS^1 peak area in negative ionization mode, to be 70 % (**Fig. S30a**).

Further purification of DMAPP was performed with Flash chromatography, using Büchi FlashPure EcoFlex diol 4 g cartridge connected to Büchi Pure C-850 FlashPrep (Flawil, Switzerland). The eluents (Buffer A and B) used were the same as those used for HILIC-UHPLC-ESI-IT-MS^n. The synthesized DMAPP was resolubilized with Buffer A. The elution was performed with a flow rate of 15 mL/min with the following program: 1 column volume (CV) isocratic 100 %B; 20 CV linear gradient 100-50 %B; 5 CV isocratic 50 %B; 1 CV linear gradient 50-100 %B; 10 CV isocratic 100 %B. As DMAPP and the impurities could not be monitored via detection by UV or ELSD in the FlashPrep system, the obtained fractions were analyzed by direction injection ESI-IT-MS^n. Fractions containing DMAPP ($[M-H]^-$ *m/z* 245 in MS NI, with MS^2 fragments *m/z* 227, 79, 159) (**Fig. S30b**) without impurities were pooled. Pooled fractions underwent solvent evaporation under reduced pressure and lyophilization, yielding 251 mg of the purified DMAPP. The purified DMAPP was assessed in an enzymatic reaction again, yielding a comparable substrate conversion to the reaction using DMAPP standard (**Fig. S30c**).

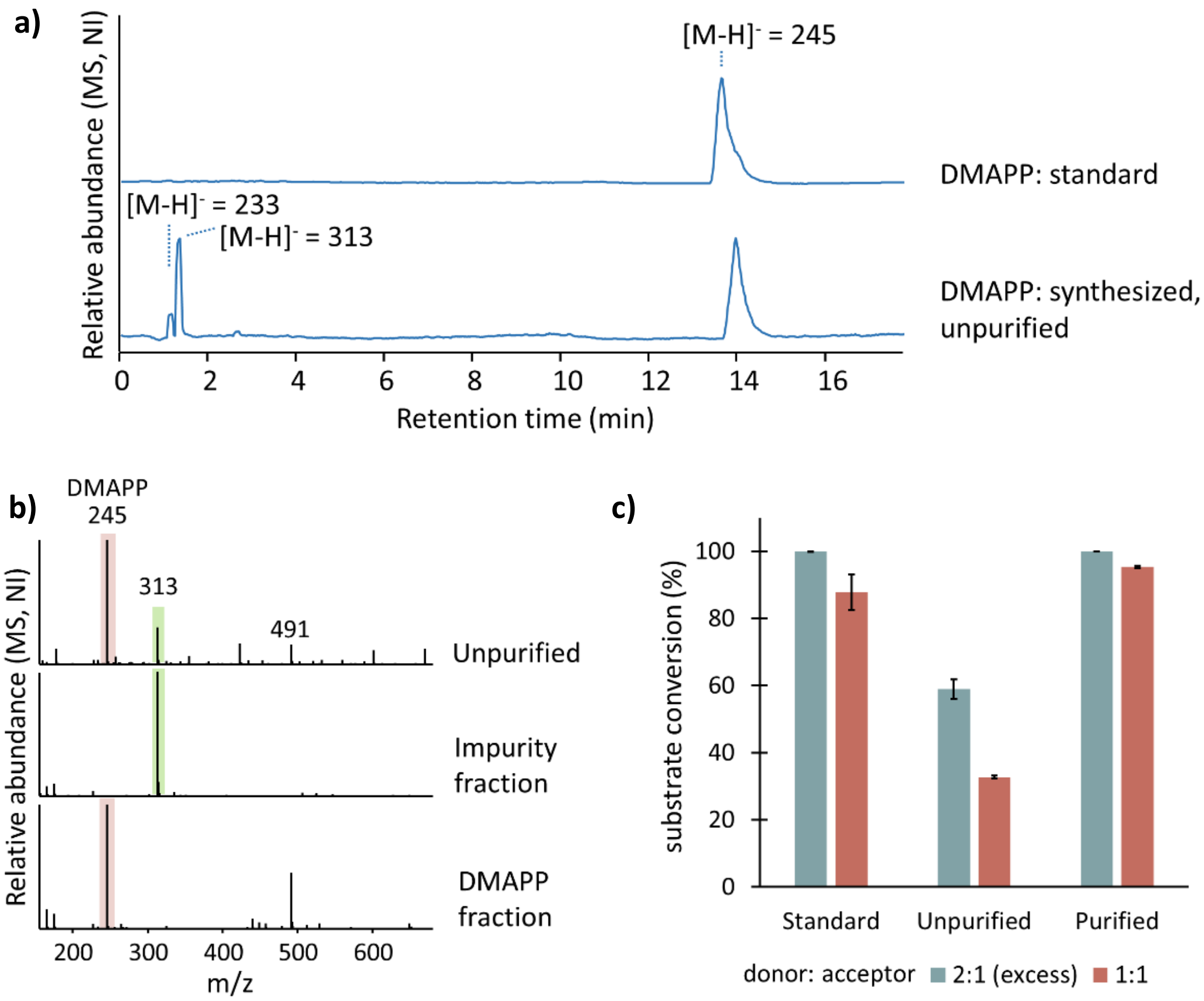

Fig. S30 a) HILIC-UHPLC-ESI-IT-MS NI chromatogram of DMAPP standard and synthesized, unpurified DMAPP obtained after ion-exchange and lyophilization, injected with a concentration of 1 mg/mL; **b)** MS NI spectra of unpurified DMAPP, a fraction containing the main impurity, and a fraction containing DMAPP, obtained from a direct injection to ESI-IT-MS^n. The corresponding *m/z* of 245, 313, and 491 were $[M-H]^-$ of DMAPP, an unidentified impurity, and in-source dimer ($[2M-H]^-$) of DMAPP; **c)** substrate conversion (%) of L-tryptophan in the presence of RePT and different DMAPPs. Two ratios of the concentration of DMAPP:L-tryptophan were used (2:1 and 1:1).

Upscaled production of prenylated compounds

*N*1-prenyl-L-tryptophan (**1a**) and *C*7-prenyl-L-tryptophan (**1b**)

The reaction was performed in 100 mL of 50 mM Tris/HCl pH 7.5 buffer containing 1 mM substrate, 1.9 mM DMAPP, and 0.1 mg/mL RePT. The reaction mixture was incubated in an oven at 37 °C for 24 h. Due to protein aggregation formed when using rotation or shaking to stir the reaction mixture, the mixture were left incubated without agitation. The enzyme was then removed by filtration using an ultra-centrifugal filter with 30 kDa molecular weight cut-off. The permeate was lyophilized, resuspended in 15 % (*v/v*) acetonitrile containing 1 % (*v/v*) formic acid, and purified using flash chromatography. The solution was loaded onto a 12 g FlashPure ID C18 40 µm connected to a Pure C-850 FlashPrep system (Büchi, Flawil, Switzerland). The flow rate used was 30 mL/min with the following eluents: (A) water containing 1 % (*v/v*) formic acid and (B) acetonitrile containing 1 % (*v/v*) formic acid. The elution program was as follows: 2 CV isocratic at 15 %B; 11 CV linear gradient of 15-55 %B; 1 CV linear gradient of 55-100 %B; 0.5 CV linear gradient of 100-15 %B; 1 CV isocratic at 15 %B. The presence of prenylated product was monitored with UV 280 nm detection. The composition of the collected fractions was confirmed with RP-UHPLC-PDA-ESI-IT-MS^n using the same method as described in substrate specificity test. Fractions containing the compound of interest were pooled,

underwent solvent evaporation using rotary evaporation under reduced pressure, and were further dried under a nitrogen stream overnight at 35 °C. The sample was lyophilized and stored at −20 °C for further investigation.

*O*4-prenyl-L-tyrosine (**2b**) and *O*4′-prenyl-resveratrol (**8b**)

The reactions were performed in 35 mL of 50 mM Tris/HCl pH 7.5 buffer containing 5 mM $CaCl_2$, 1 mM substrate, 1.8 mM DMAPP, and 0.1 mg/mL RePT for producing **2b** and 2 mg RePT/mL for **8b**. The reaction mixtures were incubated in an oven at 37 °C for 24 h without agitation. The enzymatic products were purified by using solid-phase extraction with Sep-Pak C18 6 cc Vac cartridge with 500 mg sorbent column (Waters , Milford, MA, USA) connected to an SPE vacuum manifold system (Waters, Milford, MA, USA). The column was conditioned with 2 x 6 mL of acetonitrile and equilibrated with 5 x 6 mL 0.1 % (*v/v*) formic acid in water (**2b**) or 0.1 % (*v/v*) formic in 20 % (*v/v*) acetonitrile (**8b**). Samples were loaded on the column and the elution was performed with a series of 0-40 % acetonitrile containing 0.1% formic acid (**2b**) or 20-60 % acetonitrile containing 0.1% formic acid (**8b**). The composition of the collected fractions was confirmed with RP-UHPLC-PDA-ESI-IT-MS^n. Fractions containing the compound of interest were pooled, and were further dried under a nitrogen stream overnight at 35 °C. The sample was lyophilized and stored at −20 °C for further investigation.

References

Burkhardt I, Ye Z, Janevska S, Tudzynski B, Dickschat JS (2019) Biochemical and mechanistic characterization of the fungal reverse *N*-1-dimethylallyltryptophan synthase $DMATS1_{Ff}$. ACS Chem Biol 14:2922–2931. https://doi.org/10.1021/acschembio.9b00828

Chekan JR, Mckinnie SMK, Noel JP, Moore BS (2020) Algal neurotoxin biosynthesis repurposes the terpene cyclase structural fold into an *N*-prenyltransferase. Proc Natl Acad Sci U S A 117:12799–12805. https://doi.org/10.1073/pnas.2001325117

Chen R, Gao B, Liu X, Ruan F, Zhang Y, Lou J, Feng K, Wunsch C, Li SM, Dai J, Sun F (2017) Molecular insights into the enzyme promiscuity of an aromatic prenyltransferase. Nat Chem Biol 13:226–234. https://doi.org/10.1038/nchembio.2263

Eaton SA, Ronnebaum TA, Roose BW, Christianson DW (2022) Structural basis of substrate promiscuity and catalysis by the reverse prenyltransferase *N*-dimethylallyl-L-tryptophan synthase from *Fusarium fujikuroi*. Biochemistry 61:2025–2035. https://doi.org/10.1021/acs.biochem.2c00350

Eggbauer B, Schrittwieser JH, Kerschbaumer B, Macheroux P, Kroutil W (2022) Regioselective biocatalytic *C*4-prenylation of unprotected tryptophan derivatives. ChemBioChem 23. https://doi.org/10.1002/cbic.202200311

Fan A, Li SM (2016) Saturation mutagenesis on Arg244 of the tryptophan *C*4-prenyltransferase FgaPT2 leads to enhanced catalytic ability and different preferences for tryptophan-containing cyclic dipeptides. Appl Microbiol Biotechnol 100:5389–5399. https://doi.org/10.1007/s00253-016-7365-3

Metzger U, Schall C, Zocher G, Unsöld I, Stec E, Li SM, Heide L, Stehle T (2009) The structure of dimethylallyl tryptophan synthase reveals a common architecture of aromatic prenyltransferases in fungi and bacteria. Proc Natl Acad Sci U S A 106:14309–14314. https://doi.org/10.1073/pnas.0904897106

Trela BC, Waterhouse AL (1996) Resveratrol: Isomeric molar absorptivities and stability. J Agric Food Chem 44:1253–1257. https://doi.org/https://doi.org/10.1021/jf9504576

Woodside AB, Huang Z, Poulter CD (1988) Trisammonium geranyl diphosphate. Org Synth 66:211. https://doi.org/10.15227/orgsyn.066.0211
